# Quantitative changes in 3D contact predict changes in enhancer-promoter regulation upon cohesin depletion

**DOI:** 10.1038/s41467-026-77582-1

**Published:** 2026-09-29

**Authors:** Philine Guckelberger, Benjamin R. Doughty, Suhas S. P. Rao, Glen Munson, Yingxuan Tan, Xiangmeng Shawn Cai, Charles P. Fulco, Joseph Nasser, Drew T. Bergman, Judhajeet Ray, Evelyn Jagoda, Andreas R. Gschwind, Maya U. Sheth, Chad J. Munger, Anthony S. Tan, Kristy S. Mualim, Saul Godinez Pulido, Namita Mitra, David Weisz, Muhammad Saad Shamim, Neva C. Durand, Ragini Mahajan, Ruqayya Khan, Lars M. Steinmetz, Masato T. Kanemaki, Eric S. Lander, Erez Lieberman Aiden, Jesse M. Engreitz

**Affiliations:** 1 Department of Genome Regulation, Max Planck Institute for Molecular Genetics, Berlin, Germany; 2 Department of Biology, Chemistry and Pharmacy, Freie Universität Berlin, Berlin, Germany; 3 Broad Institute of MIT and Harvard, Cambridge, MA USA; 4 Department of Genetics, Stanford University School of Medicine, Stanford, CA USA; 5 Division of Hematology/Oncology, Department of Medicine, University of California San Francisco, San Francisco, CA USA; 6 The Center for Genome Architecture, Department of Molecular and Human Genetics, Baylor College of Medicine, Houston, TX USA; 7 Department of Biochemistry and Molecular Biology, University of Texas Medical Branch, Galveston, TX USA; 8 Department of Structural Biology, Stanford University, Stanford, CA USA; 9 The Novo Nordisk Foundation Center for Genomic Mechanisms of Disease, Broad Institute of MIT and Harvard, Cambridge, MA USA; 10 Basic Sciences and Engineering Initiative, Betty Irene Moore Children’s Heart Center, Lucile Packard Children’s Hospital, Stanford, CA USA; 11 Department of Computer Science, Stanford University, Stanford, CA USA; 12 Department of Bioengineering, Stanford University, Stanford, CA USA; 13 Department of Plant Biology, Carnegie Institute of Science, Stanford, CA USA; 14 Center for Theoretical Biological Physics, Rice University, Houston, TX USA; 15 Department of BioSciences, Rice University, Houston, TX USA; 16 Medical Student Training Program, Baylor College of Medicine, Houston, TX USA; 17 Stanford Genome Technology Center, Palo Alto, CA USA; 18 European Molecular Biology Laboratory (EMBL), Genome Biology Unit, Heidelberg, Germany; 19 Department of Chromosome Science, National Institute of Genetics, Research Organization of Information and Systems (ROIS), Yata 1111, Shizuoka, Mishima, Japan; 20 Graduate Institute for Advanced Studies, SOKENDAI, Yata 1111, Shizuoka, Mishima, Japan; 21 Department of Biological Science, The University of Tokyo, Bunkyo-ku, Tokyo, Japan; 22 Department of Systems Biology, Harvard Medical School, Boston, MA US; 23 Department of Biology, MIT, Cambridge, MA USA; 24 Gene Regulation Observatory, Broad Institute of MIT and Harvard, Cambridge, MA USA; 25 Stanford Cardiovascular Institute, Stanford University, Stanford, CA USA

**Keywords:** Functional genomics, Chromatin, Gene regulation

## Abstract

Enhancers are drivers of gene regulation thought to act via 3D interactions with promoters. However, genome-wide cohesin depletion causes only limited changes in gene expression, despite widespread loss of contact domains, raising questions about how 3D contacts and cohesin influence enhancer function. Here, we introduce CRISPRi of regulatory elements upon degron operation (CRUDO), to quantitatively assess how changes in contact frequency affect enhancer-driven gene regulation. We analyzed 1039 candidate enhancers near five cohesin-dependent genes and identified 30 significant enhancer-gene pairs, 16 with cohesin-dependent effects. While all cohesin-dependent enhancers were distal (>50 Kb), not all distal enhancers were sensitive to cohesin. Instead, changes in enhancer effects correlated with changes in enhancer-promoter 3D contact frequency, which appeared to depend on both genomic distance and other locus-specific factors. Across the genome, most predicted enhancer-gene regulatory interactions do not show substantial contact changes upon cohesin depletion, explaining why only a small subset of genes are cohesin-sensitive. Together, our results illuminate how 3D contacts tune enhancer effects on endogenous gene expression.

## Abstract (web summary)

By depleting cohesin, the authors show that changes in 3D enhancer-promoter contacts alter enhancer regulatory effects. But only a subset of enhancer-promoter pairs shows weakened 3D contacts, explaining why only some genes change in expression.

## Introduction

In mammalian genomes, spatiotemporal gene expression is largely driven by enhancers. However, how enhancers affect gene expression is not yet fully understood.

The current predominant model of how enhancers regulate genes involves physical contact in 3D — the classic “looping” model^1^. This model is supported by observations that enhancer-promoter contacts correlate with gene expression across cell types^2–7^, and that repositioning enhancers in the genome can simultaneously alter chromatin interactions and transcription^8–14^. Additionally, tethering factors that force the formation of chromatin loops can enhance gene expression^15–18^ and enhancer-gene regulatory interactions can be predicted by combining enhancer activity and enhancer-promoter 3D contact frequency, as demonstrated by the Activity-by-Contact (ABC) model^19^. Together, these observations suggest that 3D contacts contribute to enhancer function.

Recent findings challenge the long-standing view that 3D enhancer-promoter contacts are universally required for transcription. Studies have revealed that key factors involved in the formation of such 3D loops and contacts include architectural proteins such as CTCF and cohesin, which orchestrate loop extrusion^20–23^. However, the depletion of these architectural proteins has minimal impacts on gene expression, despite the loss of loops and contact domains^24–27^. These observations, as well as other recent studies^28,29^, have raised questions regarding the looping model and the role of cohesin and CTCF in mediating these physical interactions, prompting alternative hypotheses. One proposed idea is that enhancer-promoter physical interactions may occur through other mechanisms, such as transcription factor affinity, compartmentalization, or via other factors^30–32^. Others suggest that while cohesin and CTCF are necessary for establishing initial contacts, they may not be essential for their maintenance^33^. Alternatively, it has been proposed that the requirement of cohesin/CTCF for mediating contacts may be specific to certain enhancer-promoter pairs, such as long-range or inducible physical interactions^12,18,34–37^. However, because most of these insights derive from synthetic systems or perturbations at a handful of developmental model loci, they may not fully capture the diversity of enhancer-promoter configurations found genome-wide^38,39^, leaving open how broadly these principles apply in an endogenous context. As a result, two fundamental questions persist: Are enhancer-promoter contacts a driver or merely a consequence of transcriptional activity^1,40^? What specific roles does cohesin play in establishing and maintaining these interactions^39,41,42^?

One approach to resolve these questions would be to examine how altering 3D contacts, such as through depletion of cohesin, changes the regulatory effects of individual enhancers on their target promoters. However, such experiments have been challenging because genes are usually regulated by multiple enhancers, and each individual enhancer may only have a small effect on gene expression.

Here, we directly test how 3D enhancer-promoter contacts influence gene regulation across many endogenous loci by developing CRUDO (CRISPRi of regulatory elements upon degron operation), a highly sensitive strategy for perturbing regulatory elements while controlling contact frequency. By combining high-throughput enhancer perturbations with acute, auxin-inducible depletion of cohesin, we systematically measured the regulatory effects of all candidate enhancers for five genes in their endogenous genomic loci before and after cohesin loss.

We find that changing 3D contact frequency quantitatively tunes the effects of enhancers on gene expression, and that a subset of distal enhancers (e.g., > 50Kb) are far more dependent on cohesin to facilitate both their 3D contacts and regulatory effects. Such contacts appear to depend not only on genomic distance and cohesin, but also on cohesin independent effects that drive contacts between active enhancers and promoters consistent with a model of compartmentalization. By exploring maps of enhancer-promoter regulatory interactions from the ABC model and from previous perturbation experiments, we find that most genes are not regulated by strong cohesin-sensitive enhancers and consequently would not be expected to require cohesin for their expression. These data demonstrate that enhancer-promoter contact frequency quantitatively tunes enhancer-promoter regulation, that cohesin depletion selectively alters the regulatory effects of a subset of distal enhancers, and that such enhancers are rare in human gene regulation. Together, these observations could explain why cohesin depletion immediately alters the expression of only a small number of genes, and illuminate how the 3D architecture of the genome tunes gene expression.

## Results

### Predicting effects of cohesin depletion with the activity-by-contact model

To study how cohesin-mediated 3D contacts affect enhancer regulation, we first constructed a regulatory map of enhancers in a human colorectal cancer HCT-116 cell line engineered to express RAD21 (a major subunit of the cohesin complex) fused to an auxin-inducible degron^25,43^. We previously used this cell line to show that 3D loops and domains disappear after 6 h of depletion of cohesin, and found that only 146 genes change by more than 1.75-fold in expression levels^25^. These limited changes in gene expression, despite widespread contact loss, raised the question of whether predicted enhancers, which have distinct chromatin features and regulatory potential compared to generic genomic regions, also experience similar reductions in contact or whether their interactions might be maintained through other mechanisms or compensatory activity^30–33,36^. To investigate this, we applied the ABC model to examine changes in predicted enhancer-gene regulatory interactions in the presence and absence of cohesin, leveraging DNase-seq and condition-matched H3K27ac ChIP-seq to estimate enhancer activity and matched ENCODE Hi-C data to quantify 3D contact frequency.

**Fig. 1:**
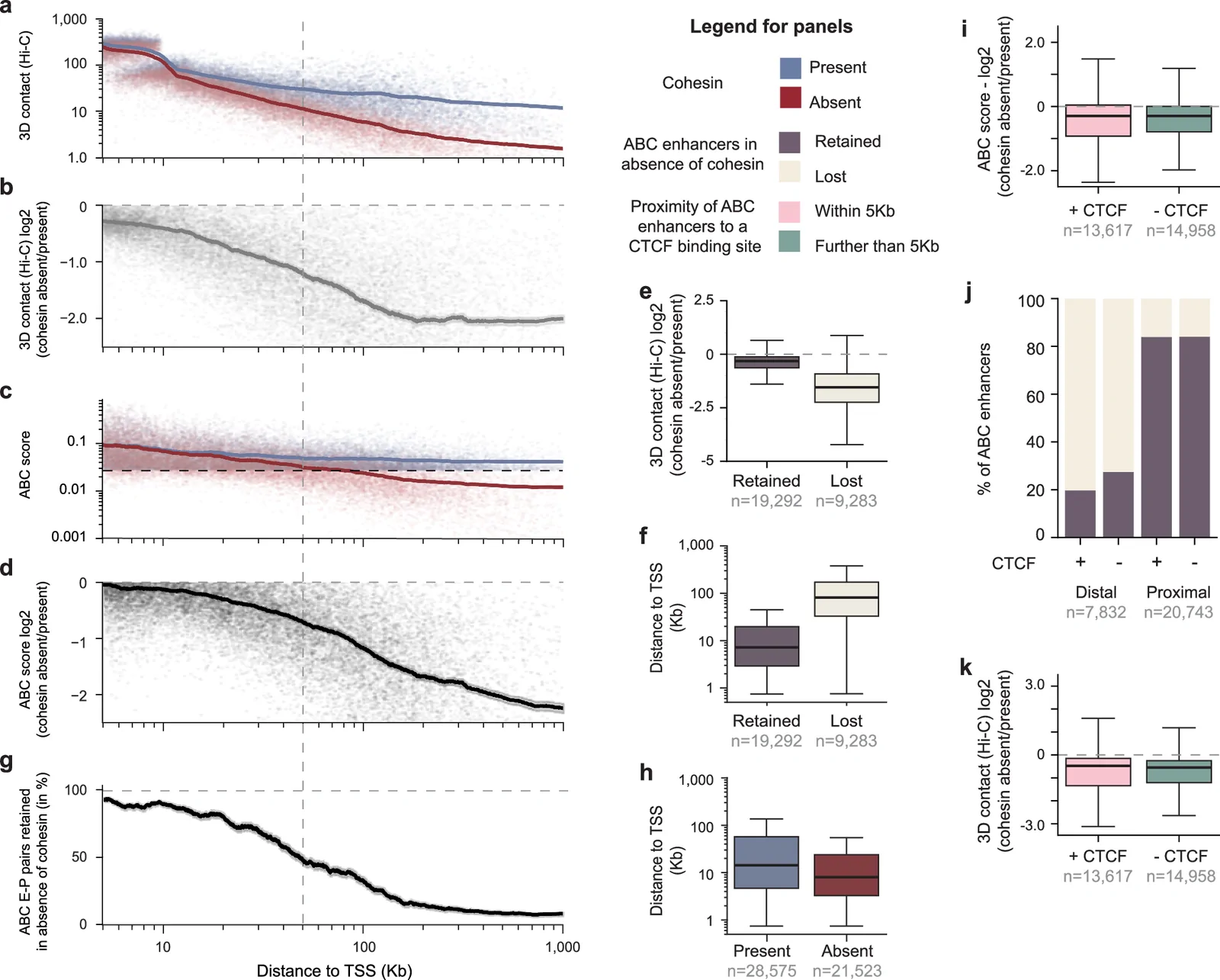
ABC predicts changes in enhancer effects upon cohesin removal. **a** Enhancer-promoter 3D contact frequency in the presence (no auxin) or absence (6 h auxin) of cohesin, as a function of distance from enhancer to target promoter, for all ABC-predicted enhancer-gene pairs in the cohesin present condition. Hi–C values are SCALE-normalized counts at 5 Kb resolution, depth 1.1 (present) and 1.5 (absent) billion contacts (manuscript in preparation). Scatter points represent individual enhancer-gene pairs (*n* = 28,575); the line represents their rolling average smoothed over 1000-point windows; shading represents its 95% c.i. Horizontal dashed line at 50 Kb. **b** As in **a** showing the log_2_ fold-change in contact frequency between the absence and presence of cohesin as a function of distance. **c** As in **a** showing ABC score as a function of distance; dashed line marks the ABC score threshold (0.027). **d**, As in **a** showing the log_2_ fold-change in ABC score as a function of distance. **e** Change in 3D contact frequency between conditions, for interactions retained (*n* = 19,292) or lost (*n* = 9283) in the absence of cohesin. Boxes represent the median and IQR (25th-75th percentiles); whiskers extend to the most extreme points within 1.5x IQR. **f** As in **e** showing the distances from enhancer to target promoter for retained (*n* = 19,292) or lost (*n* = 9283) interactions. **g** As in **a** percentage of interactions (cohesin present) that pass the ABC score threshold (0.027) in the absence of cohesin (retained enhancers, *n* = 19,292), as a function of distance. **h** Distribution of distances from enhancer to target promoter for all interactions in the presence or absence of cohesin; boxes as in (**e**). **i** Log2 fold-change in ABC score between conditions, for interactions near CTCF sites (+CTCF, *n* = 13,617) or not near CTCF sites (−CTCF, *n* = 14,958; CTCF ChIP-seq in HCT116); boxes as in (**e**). **j** Percentage of distal and proximal ABC enhancer interactions, near or not near CTCF sites, retained or lost in the absence of cohesin. **k** Log_2_ fold-change in 3D contact frequency between conditions for interactions +CTCF (*n* = 13,617) or −CTCF (*n* = 14,958); boxes as in (**e**).

To understand the effects of cohesin depletion on enhancer-promoter physical and regulatory interactions, we first summarized the changes in 3D contact frequency between promoters and ABC predicted enhancers as measured by new, higher-resolution ENCODE Hi-C data (An atlas of nuclear architecture resolves element-to-element chromatin loops in over 60 human tissues, in preparation). Removing cohesin leads to an overall drop in contact frequency between promoters and ABC-predicted distal enhancers—with stronger decreases in 3D contact for element-promoter pairs located at longer distances (Fig. 1a). For example, element-promoter pairs located over 50 Kb apart decreased in contact frequency by 62.2% on average, whereas pairs located less than 50 Kb apart decreased in contact frequency by 18.6% on average (Fig. 1b). This is consistent with a model where cohesin-mediated loop extrusion promotes the formation of longer-range 3D interactions, but does not have a strong impact on the frequency of shorter-range interactions, where polymer dynamics appear to be sufficient for frequent 3D contacts^44,45^. Notably, the degree of reduction in 3D contacts was variable among distal enhancers and did not reach a baseline expected from distance-dependent decay alone, highlighting that a single power-law does not adequately capture contact frequency across genomic scales (Supplementary Fig. 1a). This suggests that there may be additional mechanisms, such as compartmentalization effects, that maintain enhancer-promoter contacts even upon removal of cohesin^30,31,33^.

In subsequent analyses, we consider all DNase I hypersensitive site (DHS) peaks as candidate regulatory elements. Among these, we define enhancers as non-promoter elements that are either predicted by the ABC model or experimentally validated to regulate a target gene. We further classify elements as distal (>50 Kb from the TSS) or proximal (<50 Kb), based on the observation that contact frequency changes by ~2-fold at this threshold (Fig. 1b).

To explore how these changes in 3D contacts might affect enhancer-promoter regulatory interactions, we compared ABC predictions in the presence and absence of cohesin (Supplementary Data 1). We note that the ABC model assumes that the effect of an enhancer on target gene expression is proportional to the product of intrinsic enhancer activity and enhancer-promoter 3D contact frequency. Overall, enhancer activity, as estimated by H3K27ac, remained largely stable between conditions, while 3D contacts changed more substantially (Supplementary Fig. 1b). Accordingly, changes in ABC scores closely reflected changes in enhancer-promoter contact frequencies (Supplementary Fig. 1c), suggesting that cohesin depletion primarily impacts predicted regulatory interactions by disrupting 3D contacts.

In the presence of cohesin, ABC predicted 28,575 enhancer-gene regulatory interactions, including at least one ABC enhancer for 10,393 genes (see “Methods”, Supplementary Fig. 1d). Following cohesin depletion, the pronounced decrease in contact frequency causes many enhancers to fall below the ABC significance threshold and consequently predicted interactions fall to 21,523, and linked genes to 9489 (Fig. 1c–e and Supplementary Fig. 1e,f). For example, 84.0% of enhancer-promoter pairs located over 50 Kb apart were lost upon depletion of cohesin, compared to only 23.7% of enhancer-promoter pairs located less than 50 Kb apart (Fig. 1f, g see “Methods”). As a result, the predicted enhancer-promoter landscape shifted toward shorter-range interactions, with the 90th percentile interaction distance dropping from 156.3 Kb to 69.2 Kb (Fig. 1h).

Importantly, the pronounced decrease in ABC scores affected enhancers both adjacent to CTCF sites (within 5 Kb of a CTCF ChIP-seq peak, see “Methods”) and those located elsewhere (Fig. 1i, j). This indicates that 3D contact loss occurs not only at CTCF mediated loops, stripes, and domain boundaries but also throughout contact domains, impacting enhancer-promoter pairs irrespective of CTCF proximity (Fig. 1k).

We replicated these analyses with the newer and more accurate ENCODE-rE2G model^46^, which predicted the same relationship where distal enhancers are preferentially lost in the absence of cohesin (Supplementary Fig. 2, Supplementary Data 1).

Together, these analyses show that, in addition to the loss of loops and contact domains^25^, loss of cohesin can lead to a strong decrease in quantitative contact frequency for candidate enhancer-promoter pairs located at long distances. Accordingly, the ABC model predicts that a subset of distal enhancers is strongly dependent on cohesin for their regulatory effects on gene expression, whereas enhancers located close to their target genes are not. Such an effect would be consistent with recent observations that recruiting synthetic transcription factors to either distal or proximal locations showed differential cohesin sensitivity^18^. We next set out to test this directly for endogenous enhancers in the genome using CRISPR perturbations.

### CRUDO measures the effect of 3D contacts on enhancer-promoter regulation

**Fig. 2:**
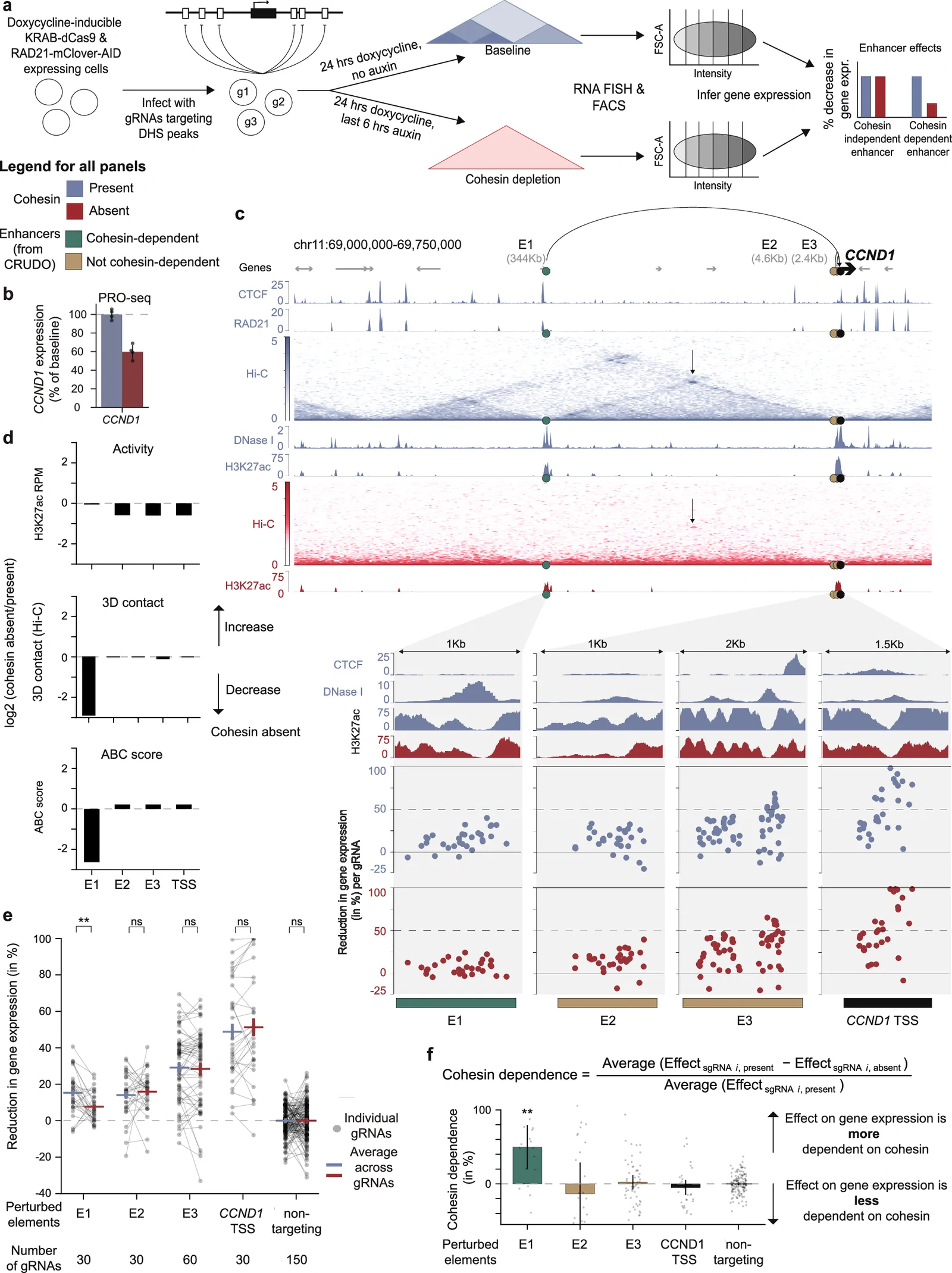
CRUDO-FF measures endogenous enhancer effects under controlled changes in quantitative contact frequency. **a** Schematic of the CRUDO-FF method. Cells express RAD21 tagged on both alleles with mClover and an auxin-inducible degron (AID), and dCas9-KRAB from a doxycycline-inducible promoter, and are transduced with a pool of gRNAs targeting DHS peaks around the gene of interest. In the cohesin present condition, cells receive doxycycline for 24 h to activate CRISPRi; in the cohesin absent condition, auxin is added for the final 6 h to degrade RAD21. Cells are labeled by RNA FISH, sorted by fluorescence into 6 bins, and gRNAs in each bin are sequenced to quantify each gRNA’s effect on expression per condition. Bar plot shows exemplary results: a cohesin-independent enhancer (equal effect) and a cohesin-dependent enhancer (stronger effect with cohesin present). **b** *CCND1* transcript levels in the presence or absence of cohesin, measured by PRO-seq. Data are mean values; error bars represent the 95% c.i. across 4 biological replicates. **c** *CCND1* locus showing DNase I hypersensitivity, CTCF ChIP-seq, RAD21 ChIP-seq, Hi-C at 5 Kb resolution (SCALE-normalized counts, Hi-C depth = 1.1 (no auxin) and 1.5 (6 h auxin) billion contacts, manuscript in preparation), and H3K27ac ChIP-seq in the presence or absence of cohesin. Coordinates (hg38): chr7:121,100,000-121,800,000. Zoom-ins: Bars at the bottom mark elements targeted in the pilot CRUDO-FF screen (enhancers E1-3 and the CCND1 TSS). Dots show CRUDO-FF effects on *CCND1* expression for individual gRNAs per element (% reduction, normalized to the mean of all non-targeting gRNAs within each condition). **d** Log_2_ fold-change in H3K27ac (top), 3D contact frequency (middle), and ABC score (bottom) in the absence versus presence of cohesin. **e** CRUDO-FF effect sizes on *CCND1* expression for individual gRNAs in the presence or absence of cohesin. Horizontal lines and error bars: mean (+/− 95% c.i.) for each element. ns: *P*_*BenjaminiHochberg*_ > 0.05, **: *P*_*BenjaminiHochberg*_ < 0.01 (gRNA-based paired two-sided 2-sample *t*-test; exact *p*-values in Supplementary Data 2b). **f** Cohesin dependence for each element: the change in its effect on gene expression between cohesin present and absent conditions. Bar: mean across gRNAs for each element. Error bars: 95% c.i.; statistical test and exact *p*-values as in (**e**); **P < 0.01.

To experimentally test the role of 3D contact frequency in enhancer regulation, we introduced CRISPRi of Regulatory elements Upon Degron Operation (CRUDO)—a highly sensitive method to test, for many enhancer-promoter pairs in parallel, whether their regulatory interactions depend on a particular protein of interest. In this approach, we apply CRISPRi pooled screens in a degron-engineered cell line to perturb putative enhancers in their endogenous context in the genome and measure the effects on gene expression in the presence and absence of the tagged protein (Fig. 2a). To read out effects on gene expression, we implemented CRUDO with either RNA fluorescence in situ hybridization and cell sorting (FlowFISH^19^), or single-cell targeted Perturb-seq (DC-TAP-seq^47,48^) assays (see below, Supplementary Data 2).

To apply CRUDO to study the effects of cohesin-mediated 3D contacts, we further engineered the previously generated HCT-116 RAD21 degron cell line^25,43^ to additionally express a dCas9-KRAB fusion (CRISPR interference, CRISPRi) linked to a blue fluorescent protein (BFP) under a doxycycline-inducible promoter. We confirmed that this doubly engineered cell line retained the previously observed gene expression changes upon depletion of cohesin (Supplementary Fig. 3a), and confirmed the efficacy of the dCas9-KRAB repression using previously validated guide RNAs (gRNAs, Supplementary Fig. 3b, c, and Supplementary Data 3). We conducted CRUDO screens in this cell line by first transducing cells with a lentiviral library of gRNAs such that each cell received only one gRNA. We next induced dCas9-KRAB mediated enhancer repression by adding doxycycline for 24 h and, in a second condition, we additionally depleted cohesin by adding auxin for the last 6 h of the enhancer perturbation. This allowed us to measure changes in the strength of regulatory effects in the presence and absence of cohesin according to simulations considering mRNA half-lives and the rate of induced repression (see “Methods”, Supplementary Fig. 4). We harvested cells from each condition and measured the effects on gene expression using either FlowFISH or DC-TAP-seq (see “Methods”). For FlowFISH screens, we used RNA FISH to label the mRNA of interest, sorted cells into six bins ranging from low to high target gene expression, used next-generation sequencing to measure the frequency of each gRNA in each bin, and used maximum likelihood estimation to infer the effects of each gRNA on gene expression compared to negative control (non-targeting) gRNAs in the same pool of cells (Fig. 2a). A key advantage of this CRUDO-FlowFISH (CRUDO-FF) approach is that we obtain precise measurements of the effect size of each candidate element on gene expression, by examining many thousands of single cells per gRNA, multiple gRNAs (2-164) per element, and hundreds to thousands of negative control gRNAs in a single pooled experiment.

We hypothesized that cohesin loss disrupts enhancer-promoter interactions. For genes whose expression decreases upon cohesin depletion, we reasoned that this reflects disruption of cohesin-dependent enhancers that require cohesin-mediated 3D contact to regulate transcription. Therefore, we expected that perturbing such enhancers would show strong effects on gene expression when cohesin is present, but reduced effects when cohesin is absent, because their regulatory interaction with the promoter is already weakened upon cohesin loss. In contrast, perturbing enhancers whose 3D contact does not depend on cohesin should result in similar effects in both conditions (Fig. 2a).

To validate our approach, we first conducted a small CRUDO experiment to examine candidate enhancers for *CCND1*—a cohesin-sensitive gene whose transcription decreases by 33.4% upon depletion of cohesin, as measured by precision run-on sequencing (PRO-seq, Fig. 2b). We designed 30 gRNAs each for the promoter of *CCND1* and three of the ten predicted ABC enhancers. All elements showed comparable levels of H3K27ac in the presence and absence of cohesin, indicating the elements do not change in their intrinsic activity (Fig. 2c, d). While enhancer E1 showed a decrease in 3D contact with the *CCND1* promoter in the absence of cohesin, the other two enhancers did not, resulting in only E1 being predicted by ABC to decrease in its effect size (Fig. 2d). We performed CRUDO-FF, and found that perturbations to all 3 targeted enhancers significantly reduced gene expression relative to negative control gRNAs both in the presence and absence of cohesin (Fig. 2c, e).

We next assessed the quantitative impact of cohesin on each enhancer-gene regulatory interaction. We defined “cohesin dependence” as the degree to which the strength of the regulatory effect of an enhancer-gene pair decreases upon removal of cohesin, quantified as the ratio of lost effect to the effect in the presence of cohesin (Fig. 2f).

For example, if perturbation to an enhancer leads to a 50% decrease in gene expression in the presence of cohesin and a 0% decrease in gene expression in the absence of cohesin, its effect is fully dependent on cohesin (“cohesin dependence” = 100%). In contrast, if perturbation to an enhancer leads to a 50% decrease in gene expression in both conditions, its effect is independent of cohesin (cohesin dependence = 0%). We note that this calculation assumes that enhancers should have a consistent relative effect on gene expression across the two conditions (that is, enhancer activity combines multiplicatively with the promoter to determine gene expression), as previously observed in reporter assays^49,50^. We tested for statistical significance by assessing the consistency of the effects across all gRNAs targeting a given element and defined significantly “cohesin dependent” pairs based on a two-sample paired t-test (*P*_*BenjaminiHochberg*_ < 0.05).

In this *CCND1* pilot experiment, the promoter and two proximal enhancers were not cohesin dependent: they showed similar decreases in gene expression between the cohesin present and absent conditions (promoter: 48.8% and 51.2%; enhancers: 14.1% and 16%, 29.1% and 28.4%, cohesin present and absent, respectively; cohesin dependence = −4.9%, −13.3%, and 2.3%, respectively, Fig. 2c, e, f). In contrast, the third enhancer (E1) had a significantly smaller effect on gene expression in the absence of cohesin (cohesin present: 15.3%, cohesin absent: 7.7%, *P*_*BenjaminiHochberg*_ = 0.009; cohesin dependence = 49.7% (Fig. 2c,e,f)). Notably, these effects were consistent with the ABC predictions (Fig. 2d), because only E1 changes in its contact frequency with the *CCND1* promoter in the absence of cohesin (Fig. 2c,d). Thus, of the three tested distal enhancers, one (E1) appears cohesin dependent and the others (E2 and E3) are not (Fig. 2f).

These data demonstrate our ability to measure perturbation-induced changes in gene expression both in the presence and absence of cohesin and identify cases of cohesin-dependent and -independent enhancer-gene regulatory interactions. Thus, CRUDO provides an approach to quantitatively measure the effect of enhancer-promoter contact frequency on enhancer function in the native genomic context.

### Mapping cohesin-dependent enhancer-gene regulatory interactions

**Fig. 3:**
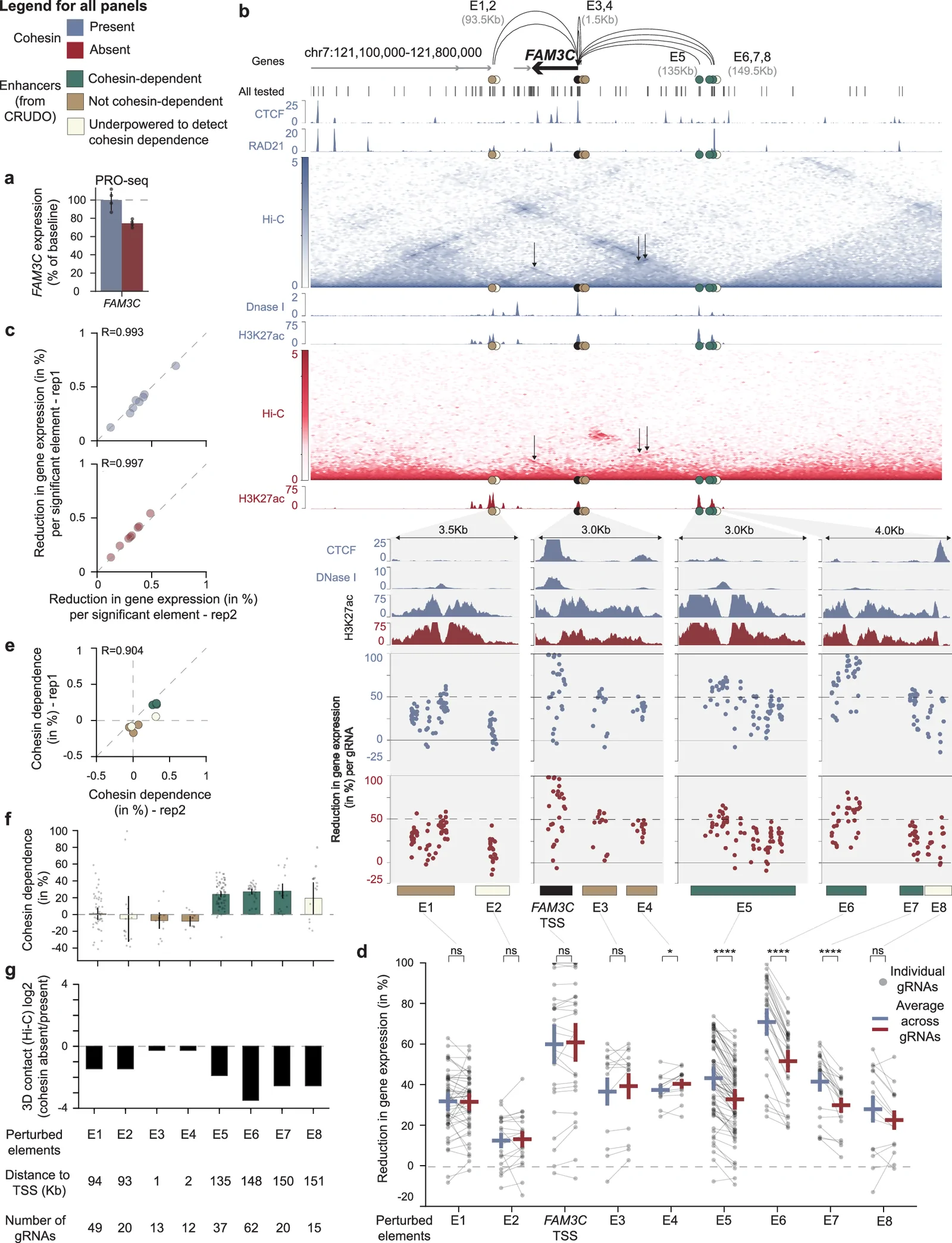
Regulatory interactions of distal enhancers decrease in the absence of cohesion. **a** *FAM3C* transcript levels in the presence (6 h of DMSO treatment) or absence (6 h of auxin treatment) of cohesin, as measured by PRO-seq. Data are mean values; error bars: 95% c.i. across 4 biological replicates. **b** *FAM3C* locus showing DNase I hypersensitivity, CTCF ChIP-seq, RAD21 ChIP-seq, Hi-C at 5 Kb resolution (SCALE-normalized counts, Hi-C depth = 1.1 (no auxin) and 1.5 (6 h auxin) billion contacts, manuscript in preparation), and H3K27ac ChIP-seq in the presence and/or absence of cohesin. Bars at top mark all elements tested in the CRUDO-FF experiment. Zoom-ins show all elements with a significant effect on *FAM3C* expression in the cohesin present condition, with bars at bottom marking these elements. Dots show CRUDO-FF effects on *FAM3C* expression for individual gRNAs per element (% reduction, normalized to the mean of all non-targeting gRNAs within each condition). **c** Correlation of element-level effects on gene expression between two biological replicate experiments, in the cohesin present (top) and absent (bottom) conditions. Dots: the eight enhancers with significant effects in the cohesin present condition (*n* = 8 elements). **d** CRUDO-FF effect sizes on *FAM3C* expression for individual gRNAs in the presence or absence of cohesin. Horizontal lines and error bars: mean (+/− 95% c.i.) for each element. Statistics were computed across individual gRNAs targeting each element (n per element in Supplementary Data 2c), from measurements averaged over 2 biological replicate experiments. ns: *P*_*BenjaminiHochberg*_ > 0.05, *: *P*_*BenjaminiHochberg*_ < 0.05, ****: *P*_*BenjaminiHochberg*_ < 0.0001 (gRNA-based paired two-sided 2-sample t-test; exact *p*-values in Supplementary Data 2c). **e** As in **c** showing correlation of cohesin dependence between two biological replicate experiments (*n* = 8 elements). **f** Cohesin dependence for each element: the change in its effect on gene expression between cohesin present and absent conditions. Bar: mean across gRNAs for each element. Error bars: 95% c.i.; derived across individual gRNAs per element as in **d**. **g**, Log_2_ fold-change in 3D contact frequencies between the absence and presence of cohesin; Hi-C as in (**b**).

We next applied CRUDO to comprehensively characterize all enhancers for five cohesin-sensitive genes—*CCND1*, *FAM3C*, *KITLG*, *SSFA2*, and *MYC*—selected on the basis of their high expression levels (enabling good detection, Supplementary Fig. 5) and the presence of ABC-predicted enhancers that exhibited diverse changes in 3D contact frequency in the absence of cohesin (Fig. 3, Supplementary Fig. 6–9). For each gene, we used CRUDO to target each element within 1 Mb and measure effects on gene expression using FlowFISH in the presence or absence of cohesin.

For example, we examined *FAM3C*, a gene whose transcription decreases by 25.6% upon depletion of cohesin (Fig. 3a). Of 96 tested elements in the locus, we identified 8 that significantly decreased the expression of *FAM3C* in the baseline (cohesin present) condition and that showed a chromatin signature of a classical enhancer (marked by H3K27ac, see Methods). These 8 enhancers were located between 1-151 Kb from the *FAM3C* promoter (Fig. 3b). The effect sizes ranged from 12-71%, and were highly correlated across two biological replicate experiments (Pearson’s *R* = 0.99, Fig. 3c). We classified 3 of these enhancers as “cohesin-dependent,” meaning their regulatory effects on *FAM3C* were significantly reduced in the absence of cohesin (P_Benjamini-Hochberg_ < 0.05, Fig. 3b, d–f). We classified 3 enhancers as “not cohesin-dependent,” defined as no significant decrease in their effects, despite having >90% power to detect a 50% decrease in cohesin dependence or a significant increase in regulatory effects in the absence of cohesin. The remaining 2 enhancers were classified as “underpowered to detect cohesin dependence,” because we lacked sufficient statistical power (<90%) to confirm a 50% cohesin dependence. Because these categories are defined in part by statistical power, we note that some groups of elements near each other in the genome contained a mix of elements classified as ‘cohesin-dependent’ and as ‘underpowered’. On average, the 3 cohesin-dependent enhancers showed a 82.3% decrease in enhancer-promoter contact frequency in the absence of cohesin, compared to only 32.2% for the enhancers that were not significantly cohesin-dependent (Fig. 3g). Interestingly, all cohesin-dependent enhancers were located at least 135Kb from the *FAM3C* promoter, while the cohesin-independent enhancers were positioned between 1Kb and 94.1Kb from the promoter, and the two additional enhancers classified as underpowered were positioned between 93Kb and 151Kb (Fig. 3b, f, g).

**Fig. 4:**
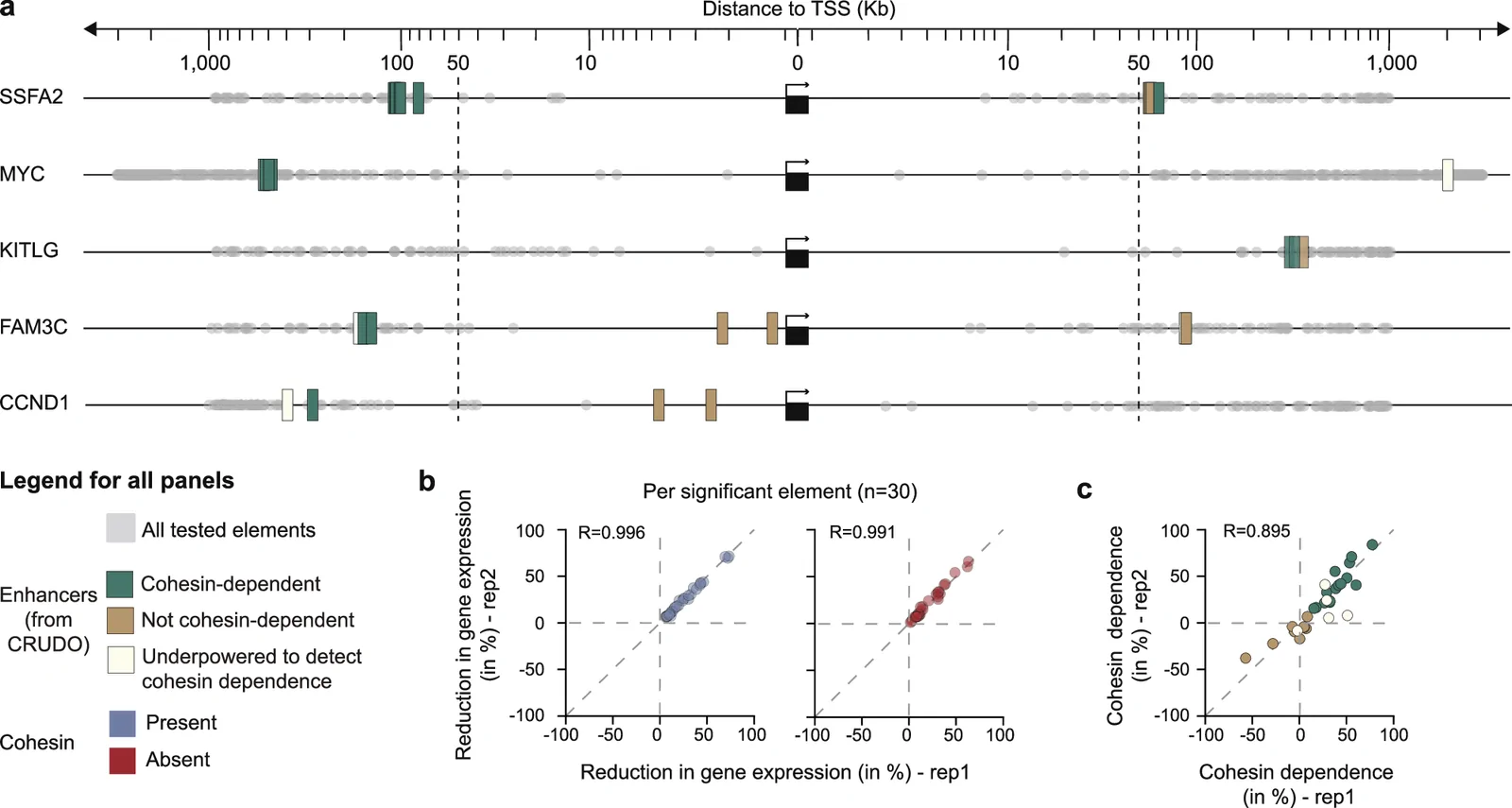
Cohesin dependence is a reproducible feature of a subset of enhancers. **a** Summary of enhancers identified by CRUDO-FF across 5 genes, showing the locations of enhancers (30 total) and their cohesin dependence relative to the target gene TSS. Teal: Cohesin-dependent enhancers (*n* = 16). Taupe: Not cohesin-dependent enhancers (*n* = 9). Ivory: Underpowered to detect cohesin dependence enhancers (*n* = 5). Light gray: All tested elements (*n* = 1039). **b** Scatterplot shows correlation of element-level effects between two biological replicate experiments in the cohesin present and absent conditions (*n* = 30 elements). **c** Scatterplot shows correlation of cohesin dependence as estimated from two separate biological replicate experiments (*n* = 30 elements).

Considering all five genes, we tested a total of 1,039 putative element-gene pairs (96-575 per gene, Fig. 4a). We identified 30 regulatory enhancer-gene pairs and quantified their precise effect sizes in both cohesin-present and cohesin-absent conditions (Pearson’s R = 0.99 for biological replicates, Fig. 4b) as well as their degree of cohesin dependence (Pearson’s R = 0.9 for biological replicates, Fig. 4c). Of these 30 pairs, 21 exhibited a nominal (>5%) decrease in regulatory effects in the absence of cohesin, while 9 did not. After significance testing, we categorized 16 element-gene pairs as “cohesin-dependent,” 9 as “not cohesin-dependent,” and 5 as “underpowered to detect cohesin dependence” (Fig. 4a). To validate these findings, we performed a second experiment using a different assay (CRUDO-DC-TAP-seq, Methods, Supplementary Data 2), and found that enhancer effects and cohesin dependence were correlated between FlowFISH and DC-TAP-seq (Pearson’s R = 0.65 for effects at baseline; Pearson’s R = 0.62 for cohesin dependence; Supplementary Fig. 10). We note that CRISPRi-based perturbation has been previously shown to identify functional enhancers^51–54^ rather than report physical proximity to promoters, and in our dataset, enhancer effect size did not correlate with either linear distance to the TSS or baseline 3D contact frequency (Supplementary Fig.11a).

### Cohesin dependence correlates with changes in 3D contact

**Fig. 5:**
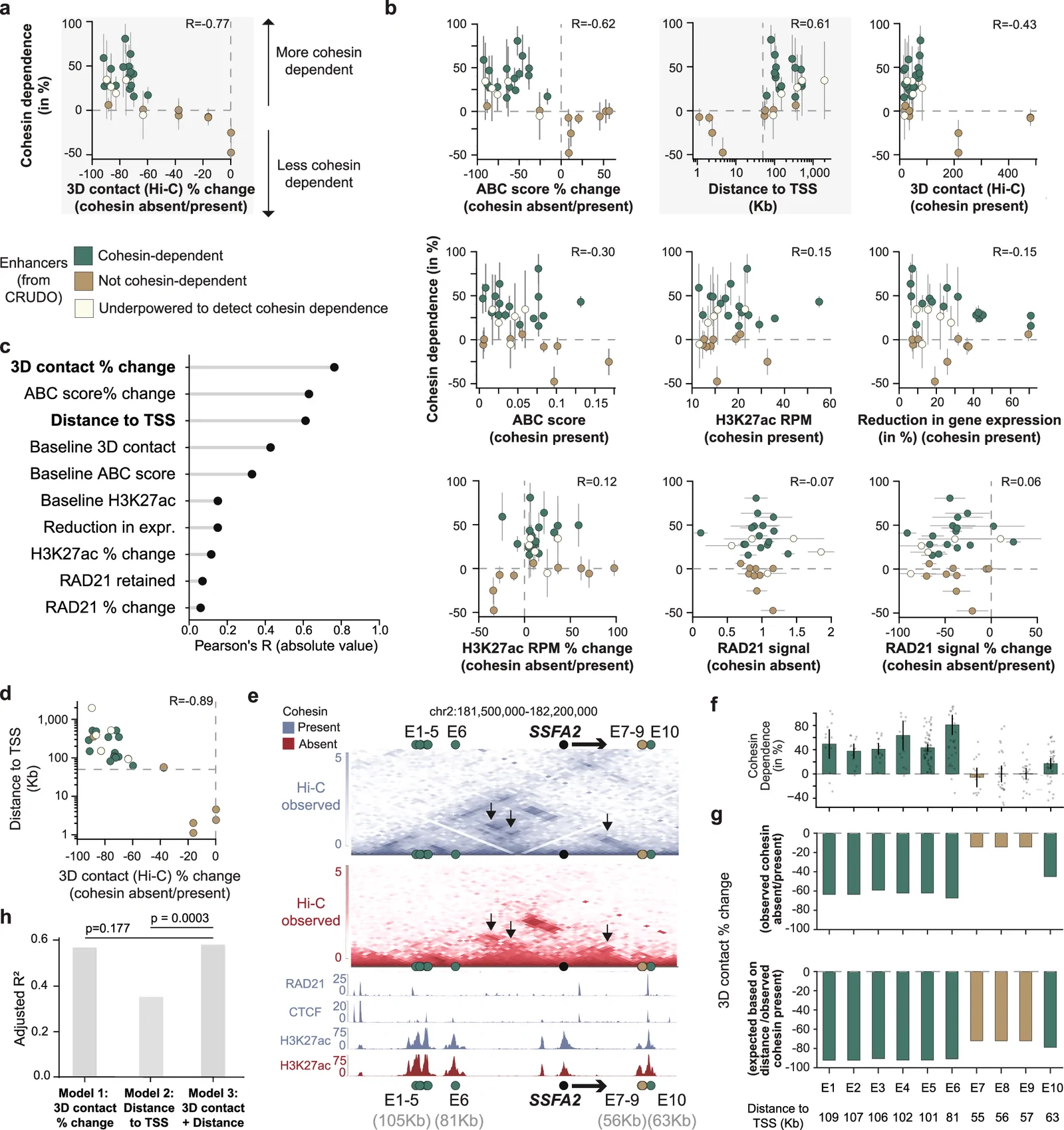
Enhancer-promoter contact frequency modulates enhancer effects. **a** Cohesin dependence versus percent change in 3D contacts between the cohesin absent and present conditions, across all enhancers (*n* = 30 elements). Points: mean cohesin dependence across gRNAs; error bars: 95% c.i. across individual gRNAs (*n* = 10-69 gRNAs per element). Hi-C values throughout are SCALE-normalized counts at 5 Kb resolution, depth 1.1 (present) and 1.5 (absent) billion contacts. **b** Cohesin dependence versus each of nine genomic features across all enhancers (*n* = 30 elements), each as mean +/−95% c.i. across individual gRNAs (*n* = 10–69 gRNAs per element). Features (x-axis labels) are ABC score, H3K27ac ChIP-seq signal, reduction in gene expression, and 3D contact, all in the cohesin present condition; genomic distance from enhancer to target gene TSS (Kb, log_10_); RAD21 signal in the absence of cohesin; and the percent change (absent versus present) in ABC score, H3K27ac signal, and RAD21 signal. **c** Absolute Pearson’s R for the correlation between cohesin dependence and each feature in (**b**), across all enhancers (*n* = 30 elements). **d** Genomic distance from enhancer to target gene TSS (Kb, log_10_) versus percent change in 3D contacts between the absence and presence of cohesin. **e** *SSFA2* locus showing Hi-C maps (as in a) in the presence and absence of cohesin with CTCF ChIP-seq, RAD21 ChIP-seq H3K27ac ChIP-seq (cohesin present) and H3K27ac ChIP-seq in the absence of cohesin. CRUDO-enhancers are marked at their genomic positions: cohesin-dependent (*n* = 7), not cohesin-dependent (*n* = 3); TSS also indicated. **f** Cohesin dependence for each element: the change in its effect on gene expression between cohesin present and absent conditions. Bar: mean across gRNAs for each element. Error bars: 95% c.i.; computed across individual gRNAs per element, from per-gRNA effects averaged over 2 biological replicate experiments (precise n per element in Supplementary Data 2c). **g** Percent change in 3D contact frequencies between the absence and presence of cohesin (upper) versus the expected distance-dependent change, calculated as the percent change between SCALE-normalized expected and observed contacts in the cohesin present condition (lower). **h** Adjusted R² values from OLS models and *p*-values from ANOVA-based comparisons of nested models: contact-only, distance-only, and contact + distance.

We used this set of 30 endogenous enhancer-gene pairs, in 5 genomic loci, to test whether changes in 3D contact lead to changes in enhancer effects. Indeed, among the 16 enhancer-gene pairs that were significantly cohesin dependent, all 16 showed a > 2-fold decrease in 3D contact upon removal of cohesin, versus only two of nine pairs that were not cohesin-dependent (odds ratio with Haldane-Anscombe correction: 37, Fisher exact *P* = 0.000075). Conversely, of the 23 enhancer-gene pairs that changed in 3D contact by more than 2-fold, 16 were significantly cohesin-dependent, versus none of the seven pairs that changed in 3D contact by less than 2-fold (Fig. 5a).

This relationship was quantitative. Changes in enhancer effect on gene expression as measured by CRUDO) were correlated with the changes in enhancer-promoter 3D contact frequencies (as measured by Hi-C, Pearson’s R = −0.77, Fig. 5a and Supplementary Fig. 11b, c), corresponding to 74% of the explainable variance in cohesin dependence based on replicate concordance (R = 0.895, Fig. 4c). This correlation held for elements regardless of their proximity to a CTCF binding site (Supplementary Fig. 11d), and was stronger than correlations with other genomic features such as baseline H3K27ac, baseline 3D contacts, baseline effect sizes, and changes in H3K27ac (Pearson’s R = 0.12–0.43, Fig. 5a–c). We observed a similar correlation with change in the ABC score (Pearson’s R = −0.62), indicating that accounting for potential changes in enhancer activity (as approximated by H3K27ac signals) did not explain additional variation in cohesin dependence (Fig. 5b, c). Cohesin dependence also did not correlate with differences in retention of cohesin binding after degron activation, based on RAD21 ChIP-seq data (Pearson’s R = 0.07, Fig. 5b, c and Supplementary Fig. 11e–g). Thus, changing 3D contact frequencies upon depletion of cohesin quantitatively tunes the regulatory effects of enhancers on gene expression, consistent with a contact-based model for enhancer function.

### Cohesin dependence correlates better with changes in 3D contact than with genomic distance

Previous studies using exogenous loci, artificial systems, or indirect Hi-C analyses have reported that distal enhancers are more sensitive to cohesin than proximal enhancers^11,12,18,34^. Our CRISPR data at 30 endogenous enhancers are consistent with these results, but also show that only a subset of distal enhancers are sensitive: while all of the cohesin-dependent enhancers were located farther than 50 Kb from their target promoter, not all distal enhancers were cohesin-dependent (Fig. 5b).

To understand why some distal enhancers are not cohesin-dependent, we examined their changes in 3D contact frequencies. Indeed, distal enhancers showed stronger reductions in 3D contacts than proximal enhancers, but to varying degrees (Fig. 5a, Supplementary Fig. 12a–c). Across all 30 enhancers, cohesin dependence correlated more strongly with changes in 3D contacts than with genomic distance (absolute Pearson’s R = 0.77 vs 0.61, Fig. 5a–c). This difference was even more pronounced when we excluded four very proximal enhancer-promoter pairs (<5Kb) (Supplementary Fig. 12d–h). Inspection of individual loci suggested that certain distal enhancers retained some degree of contact above what would be expected based on a simple distance relationship. For example, in the *SSFA2* locus, enhancers E7–9 were located 55–57 Kb away from the *SSFA2* promoter but were all cohesin-independent (Fig. 5e, f). Interestingly, these enhancers showed minimal reduction in 3D contact with the *SSFA2* promoter upon cohesin removal, despite an expected −70% decrease in contact for loci at this genomic distance (Fig. 5e, g). Hi–C maps showed that these enhancers maintained a focal loop with the promoter even after depletion of cohesin (Fig. 5e and Supplementary Fig. 12i), although the loop was quantitatively reduced in strength. More generally, upon depletion of cohesin, most enhancers, whether cohesin-dependent or independent, exhibited higher observed contact frequencies versus expected based on genomic distance (Fig. 5g). Specifically, 19 of the 30 enhancers maintained >2-fold higher contact than expected based on linear distance. These elevated contacts appeared to correspond to focal loops between active regions with high levels of H3K27ac (Figs. 3b, 5e and Supplementary Fig. 6b, 7b, 8b, 9b, 12h), matching previous observations that homotypic interactions between active elements are maintained and sometimes strengthened in the absence of cohesin^25,30,33^.

To quantify the importance of this effect across all 30 enhancers, we constructed simple linear models to correlate cohesin dependence with a combination of genomic distance and change in 3D contacts. Incorporating changes in 3D contact frequency significantly improved the distance-only model (R = 0.79 vs 0.61 respectively; F-test *p* = 0.0003), whereas adding linear distance to the 3D contact model yielded no significant improvement (R = 0.79 vs 0.77 respectively; F-test *p* = 0.177) This highlights that it is changes in 3D contacts—not genomic distance alone—that predict gene expression changes following cohesin loss (Fig. 5h, Supplementary Fig. 12j).

These data suggest that cohesin dependence varies with changes in 3D contacts, which in turn depend both on genomic distance and other factors, which could include the chromatin state of enhancers and promoters (see Discussion).

### Cohesin-dependent genes are primarily regulated by distal enhancers

Our data indicate a quantitative relationship between enhancer-promoter contact frequency and enhancer effects on gene expression, and that distal enhancers are more likely to rely on cohesin to mediate these interactions. Accordingly, we next investigated whether the sensitivity of genes to cohesin is related to features of their enhancer landscapes—specifically, to how many, how far away, and/or how strong a gene’s enhancers might be.

**Fig. 6:**
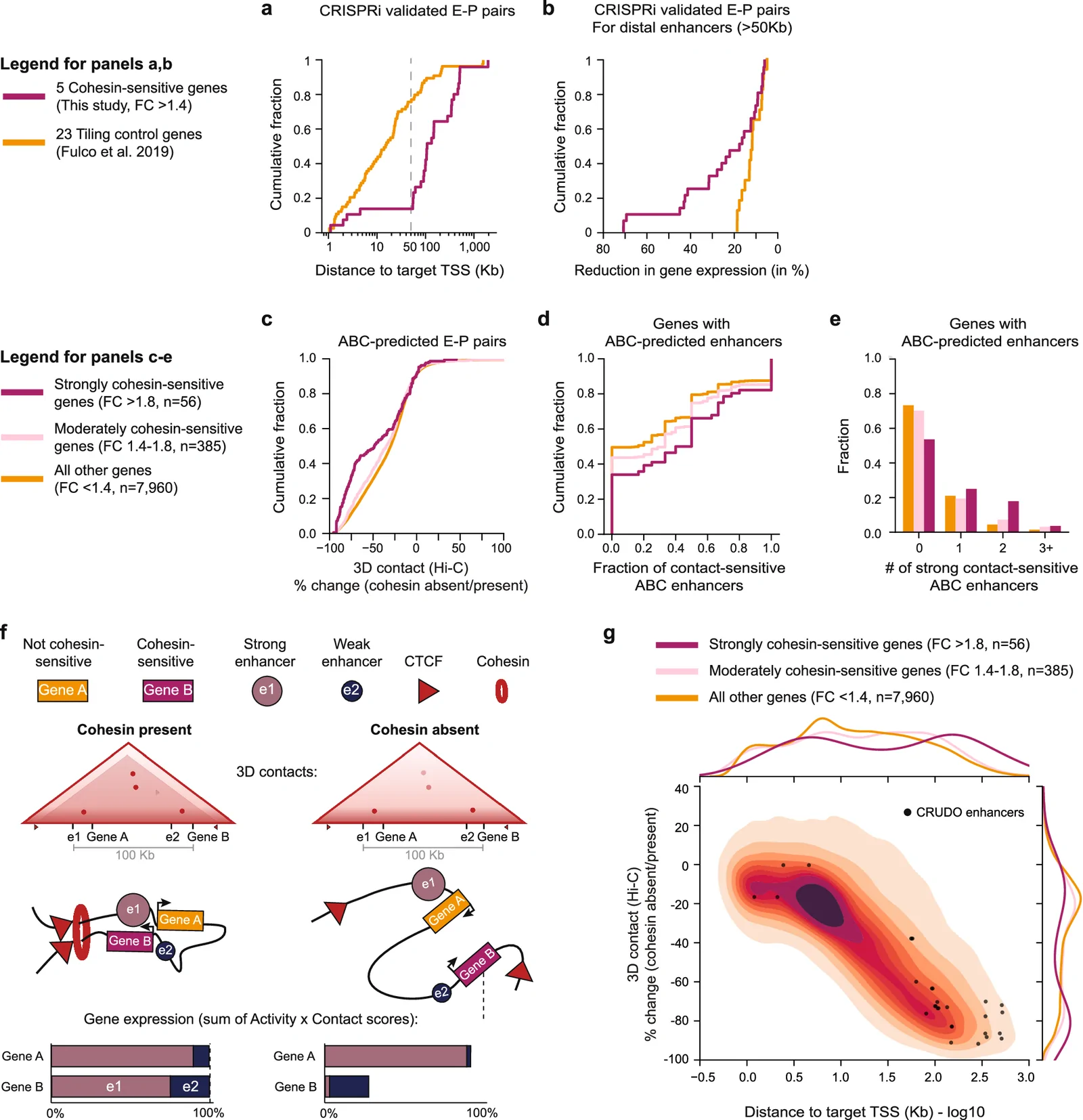
Cohesin-dependent genes rely on cohesin-dependent enhancers. Genes are classified by PRO-seq reduction (cohesin absent versus present, TPM > 5): strongly cohesin-sensitive (>1.8-fold), moderately cohesin-sensitive (1.4–1.8-fold), or all other. Contact-sensitive enhancers: >50% decrease in 3D contact upon cohesin depletion. Hi-C: SCALE-normalized counts, 5 Kb resolution, depth 1.1 (no auxin) and 1.5 (6 h auxin) billion contacts. **a** Cumulative distribution of genomic distance from enhancer to target gene TSS for each significant pair from CRISPRi tiling of 5 cohesin-dependent genes (HCT116; *n* = 30, this study) and 23 “Tiling control” genes in K562 cells, selected regardless of cohesin dependence (*n* = 71; Fulco et al.^19^). **b** Cumulative distribution of effect size for each distal enhancer (>50 Kb from TSS) among CRISPRi-validated enhancer–promoter pairs in the CRUDO-FF dataset (*n* = 30) and the Tiling control dataset(*n* = 71^19^). **c** Cumulative distribution of the change in 3D contact frequency (absent versus present) for each ABC-predicted enhancer-promoter pair in HCT116 cells, by gene category: strongly (*n* = 213), moderately (*n* = 1103), and all other (*n* = 20,893). **d** Among genes with at least one predicted ABC enhancer, cumulative distribution of the fraction of predicted enhancers that are contact-sensitive, by gene category: strongly (*n* = 56), moderately (*n* = 385), and all other (*n* = 7960). **e** Among the same genes and categories as d, the number of ABC-predicted strong contact-sensitive enhancers (>15% effect size) per gene (0–2, or ≥3). **f** Proposed model: enhancer-promoter contact frequency tunes enhancer effects, and a gene’s cohesin sensitivity depends on the strengths and locations of its enhancers. With cohesin (left), loop extrusion raises contacts among e1, e2, Gene A, and Gene B. Without cohesin (right), distal contacts (e1-Gene B, e2-Gene A) are reduced while proximal contacts (e1-Gene A, e2-Gene B) are maintained. Gene A is less sensitive (strong proximal enhancer), Gene B more sensitive (strong distal enhancer); the Gene B pattern is genome-wide rare (<30% of genes). **g** Joint density of log₁₀ enhancer-to-TSS distance versus percent change in 3D contacts (absent versus present) for all ABC enhancer–gene predictions (*n* = 26,894). Marginal densities show the three gene categories. The top and bottom 1% of values are excluded.

We compared the enhancers for the five cohesin-sensitive genes (as measured by CRISPRi-FlowFISH in the cohesin present condition) to a previous study in which we performed similar comprehensive tiling experiments on 23 “tiling control genes” that were not selected based on information about their sensitivity to cohesin depletion^19^. On average, the 5 cohesin-dependent genes we studied here were regulated by 6.2 enhancers, whereas the 23 tiling control genes had an average of 3.1 enhancers (Supplementary Fig. 13a). We observed distinct differences in the characteristics of the identified enhancers for cohesin-dependent genes compared to the control group: 87.1% (27/31) of the enhancers for cohesin-dependent genes were located distally (>50 Kb), whereas only 23.9% (17/71) of enhancers were distal in the tiling control gene set (Fig. 6a, and Supplementary Fig. 13b, c). Moreover, when analyzing the effect sizes of the distal enhancers, we found that none of the distal enhancers regulating genes in the tiling control gene set exhibited more than a 20% effect size, while nearly half (13/27) of the distal enhancers regulating cohesin-dependent genes had effects exceeding 20% (Fig. 6b and Supplementary Fig. 13b, c). These findings indicate that cohesin-dependent genes tend to be regulated by more enhancers, and that those enhancers are more distal with stronger effect sizes. We observed similar results when comparing the 5 cohesin-dependent genes to a larger set of 240 control genes^46^ studied using CRISPRi enhancer perturbation experiments with alternative designs that did not involve comprehensive tiling around individual genes (“Other control genes”,^48,51,54–61^, Supplementary Fig. 13d, e).

To determine whether these differences hold for genes beyond the five that we experimentally studied here, we compared properties of enhancers predicted by the ABC model between genes that are strongly cohesin dependent (>1.8-fold decrease in transcription upon depletion of cohesin, *n* = 56), genes that are moderately cohesin dependent (fold change between 1.4 and 1.8 decrease in transcription upon depletion of cohesin, *n* = 385) and genes that are not (<1.4-fold decrease, *n* = 7960). We identified a total of 7025 (out of 22,209) enhancers predicted to be cohesin dependent, based on their 3D enhancer-promoter contact decreasing by >2-fold upon cohesin depletion. Cohesin-dependent genes were indeed significantly enriched for cohesin-dependent enhancers, with 48.8% of their enhancers showing >2-fold contact loss, compared to 31.2% for other genes (Mann–Whitney *U* test *P *= 6.6 × 10⁻^7^; Fig. 6c). Half of cohesin-dependent genes are predicted to be regulated by mostly (>50%) cohesin-dependent enhancers (compared to just 33.4% of other genes; odds ratio = 2, Fisher’s exact *P* = 0.01; Fig. 6d), and 46.4% had at least one cohesin-dependent enhancer predicted to have a > 15% effect size (compared to just 26.7% of other genes; odds ratio = 2.4, Fisher’s exact *P* = 0.002; Fig. 6e, Supplementary Fig. 14). The downregulated genes span a wide range of expression levels (TPM 5–510, Supplementary Fig. 15a), indicating that the patterns observed at our five experimentally studied genes (all in the top 20th percentile, TPM > 60) likely extend to genes with lower expression as well (Supplementary Fig. 15b). We observed similar patterns when using enhancer–promoter distance (>50 kb) as an independent spatial proxy for 3D contact dependence, highlighting that the relationships identified in our experimentally tested genes extend genome-wide (Supplementary Fig. 15c–e).

Together, these findings reveal that most genes have few strong enhancers whose contact is dependent on cohesin. In contrast, cohesin-dependent genes have distinct enhancer landscapes: they tend to have more total enhancers, more distal enhancers whose contact is cohesin-dependent, and more strong distal enhancers compared to other genes (Fig. 6f).

## Discussion

The role of 3D enhancer-promoter contacts in gene regulation is the topic of ongoing debate^62–64^. The needed experiment—to manipulate the 3D contacts of enhancers in their endogenous locations in the genome to accurately measure effects on enhancer-promoter regulation—has proven challenging, and has been attempted at only a handful of enhancers, with mixed and subtle effects^9,13,15–18,36^. Here, we develop CRUDO to systematically test the role of 3D contacts on gene regulation at thousands of candidate enhancer-promoter pairs—producing the largest dataset to date describing the responses of many enhancer-promoter regulatory interactions to changes in 3D contacts in their endogenous genomic locations.

Our data demonstrate that reducing 3D contacts between an enhancer and target promoter almost always leads to a detectable reduction in enhancer effects on gene expression. Specifically, of the 23 regulatory enhancer-promoter pairs with reductions in 3D contact upon removal of cohesin (by >50%, Fig. 5a), 15 element-gene pairs were classified as cohesin-dependent” (16/23, 69.6%), two as “not cohesin-dependent” (2/23, 8.7%), and five as “underpowered to detect cohesin dependence” (5/23, 21.7%). These changes in regulatory effects were often small in magnitude (5–24% relative to baseline gene expression), were validated using two readouts (FlowFISH and DC-TAP-seq, Supplementary Fig. 10), and were only possible to detect due to the high sensitivity of our CRUDO approach.

Together with previous observations, our results suggest a model for how 3D contacts, influenced by both cohesin-dependent and—independent mechanisms, tune enhancer function. The pattern of 3D contacts in the genome is thought to be modulated by a combination of polymer effects, compartmentalization effects, and cohesin-mediated loop extrusion^20,22,65^. Upon removal of cohesin, CTCF-mediated loops and domains disappear, compartmentalization is maintained and perhaps strengthened^25^, and 3D contacts change across the genome^25,26^. However, the magnitude of these changes appears to depend on multiple factors. At shorter distances, 3D contacts are largely maintained (<5% changes, Fig. 6g), presumably by more random, non-extrusive movement of the DNA polymer. At longer distances, 3D contacts between certain enhancers and promoters often change more dramatically (*e.g*., by up to 89.8% Fig. 3g), for example, due to the interplay between cohesin and CTCF^8,13,66,67^. However, 3D contact in the absence of cohesin is not a simple function of distance (Fig. 6g), and can also be influenced by other locus-specific effects such as cohesin-independent compartmentalization mechanisms^25,30,33^. Our data show that these differences in 3D contacts lead to quantitative changes in the effects of individual enhancers on their target genes—with changes in gene expression correlated with the change in 3D contact (Fig. 5a). As such, proximal enhancers (*e.g*., 1–5 Kb) do not change appreciably in 3D contact frequency nor effect on gene expression, whereas a subset of distal enhancers (*e.g*., > 50 Kb) show larger changes.

Our data extend previous studies of individual enhancer-promoter pairs at engineered loci or using synthetic activators, which have emphasized the role of genomic distance in determining cohesin dependence^11,12,18^. Our data are consistent with these observations but also show that cohesin dependence is not determined by genomic distance per se, but rather by the degree of change in enhancer-promoter contact frequency. While distal enhancers are more likely to be sensitive to cohesin, the degree of sensitivity varies. This stronger correlation with 3D contacts may have been missed by some previous studies that have examined this question using recruitment of a synthetic activator^18^ or insertion of enhancers are varying distances^11,12^. In some cases, these studies did not measure 3D contact frequencies after recruitment or insertion. In others, they did and also observed increased enhancer–promoter contacts after enhancer insertion^12^, likely reflecting compartmentalization effects. By directly quantifying contact changes and enhancer effects at native loci, our study clarifies that 3D contact changes, rather than distance per se, drive cohesin dependence. Notably, a recent study also supports this conclusion, showing that the regulatory activity of an enhancer varies depending on its genomic insertion site, and is not simply a function of linear distance^68^. This may result from homotypic interactions that occur between distal enhancers and promoters or promoter-proximal elements^32,36^.

Our data help to resolve questions as to why the removal of architectural proteins like cohesin or CTCF leads to only subtle changes in gene expression^24–26,35^—such as, for example, only 146 genes changing by more than 1.75-fold upon removal of cohesin in HCT-116 cells^25^. Previous studies have attributed these subtle changes in gene expression to a variety of different factors, including the presence of long-range loops^12,18,34,69^. In contrast, other studies propose alternative models, such as the idea that CTCF is required for establishing, but not maintaining, gene expression^33^, or that CTCF binding at promoters contributes directly to transcriptional regulation^17^. Additionally, some have suggested that enhancer-promoter interactions can occur through cohesin-independent mechanisms, such as transcription factor cooperativity, compartmentalization, or other bridging factors^30–32^. To reconcile these different explanations, our data point to three observations that refine how we understand enhancer-promoter regulatory interactions: (i) Rather than a global requirement for cohesin at all enhancers, we find that cohesin quantitatively tunes 3D contacts and enhancer effects on gene expression, with much stronger effects for a subset of longer-range enhancers. (ii) Enhancers often have small effects on gene expression (median effect size in combined CRISPR perturbation datasets = 11.6-12.1%^19,46^). (iii) Only a small number of genes have strong enhancers whose 3D contact is dependent on cohesin. Based on the ABC model and perturbation results, less than 20% of genes are predicted to have a moderately strong, cohesin-dependent enhancer (>15% effect on gene expression with >2-fold decrease in contact, Fig. 6e). Together, due to the stronger influence of cohesin at distal enhancers and the distribution of enhancer locations in the genome, only a small number of genes would be predicted to have cohesin-dependent enhancers, and therefore the changes in gene expression due to acute removal of cohesin are expected to be small for most genes (Fig. 6f). Nevertheless, small changes to gene expression can have functional consequences, and we expect that many of these cohesin-dependent effects could have functional roles in the cell which then lead to further and larger downstream changes in gene expression, as previously proposed to occur during macrophage activation^70^. For example, Cyclin D1 (*CCND1*), one of the cohesin-dependent genes we study here, is a key cell cycle regulator involved in the G1 to S transition^71^. Reduced *CCND1* expression has been shown to induce G0/1 arrest and a slower progression through S-phase^72^, while increased *CCND1* expression—frequently observed in cancer—is associated with an accelerated transition through the cell cycle^73^, highlighting the importance of tightly controlled *CCND1* expression levels. Our findings also help resolve discrepancies by emphasizing the cell type-specific nature of cohesin dependency: the genes impacted by cohesin depletion may vary depending on which enhancers in a given cell type rely on cohesin to form 3D contacts.

While we found that quantitative changes in 3D contacts and regulatory effects of enhancers were correlated (Pearson’s R = −0.77, Fig. 5a), the relationship was far from perfect, and we were unable to address questions such as whether regulatory effects vary linearly^19,74^ or nonlinearly^11^ with respect to 3D contact. This may be due to other technical or biological factors that highlight directions for future work. (i) While we have carefully controlled the timing of cohesin removal using an auxin-inducible degron, the temporal resolution of CRUDO-FF is still limited by the half-life of target mRNAs and induction time of dCas9-KRAB. Due to these factors, it is possible that our measurements of cohesin dependence underestimate the true effects (*i.e*., due to mature mRNA levels not representing instantaneous effects on transcription) or conflate the effects of direct, *cis* effects with indirect, *trans* effects (*i.e*., due to downstream effects of changing *cis* target gene expression during the 24 h of CRISPRi repression). (ii) Estimates of 3D contact frequencies depend on the resolution at which Hi-C data is collected and analyzed^74,75^, and so collecting Hi-C data at even greater depth may improve our analysis. (iii) We find that changing 3D contact frequencies, as measured by Hi-C, changes enhancer regulation, but these observations are possibly compatible with various models for defining functional enhancer “contacts”, including ones where enhancer and promoter DNA may be separated by dozens of nanometers or where functional “contacts” involve larger clusters of transcriptional factors and cofactors^28,70,76,77^. (iv) We note that the 5 genes studied here are all among the top 20% of highly expressed genes, with this selection due to technical requirements of detection using RNA FlowFISH (Supplementary Fig. 5). We expect that our findings extend to genes with lower expression, because genes of many expression levels are cohesin dependent (Supplementary Fig. 15a) and because these genes with lower expression also are predicted to have cohesin-dependent enhancers (Supplementary Fig. 15b). (v) Additional biological changes that occur with cohesin depletion could affect the relationship between pairwise enhancer-promoter contacts and their regulatory effects, including for example changes in enhancer-enhancer contacts that can impact regulation^46^ or changes in the sensitivity of promoters to distal enhancers^49,50^ (Supplementary Note 1). Future work with additional temporally or spatially resolved assays will help to address these questions and further refine our understanding of the functional relationship between genome architecture and enhancer effects on gene expression.

In summary, we developed an approach to systematically investigate how cohesin-mediated 3D contacts influence gene expression, revealing general rules of gene regulation that will aid in studying how cohesin and distal enhancers coordinate cellular differentiation and signaling pathways in health and disease. Our approach can be adapted to examine the role of other architectural or transcriptional regulatory proteins in enhancer regulation, which we expect will further our ability to differentiate between chromatin architecture as a packaging tool and its function in regulating gene expression.

## Methods

### Generating enhancer predictions

We used the ABC model^19^ to predict enhancer-gene pairs in the presence and absence of cohesin (Supplementary Data 1a, b) from measurements of chromatin accessibility (DNase-seq), histone modifications (H3K27ac ChIP-seq), and chromatin conformation (Hi-C) in untreated and 6 h auxin treated HCT-116 RAD21-mAID-mClover^25^ as previously described. In brief, we use MACS2 with a *p*-value cutoff of 0.1 to call peaks from baseline (cohesin present) chromatin accessibility data (ENCFF166PCV and ENCFF619LVU, Supplementary Data 4), defining the elements for which predictions are generated in both cohesin present and absent conditions. We resize the peaks to 500 bp centered around the highest signal of the peak, include peaks around the TSS of each gene, and exclude blacklisted regions. For the predictions, we consider all element-gene pairs where the element is within 5 Mb of the gene TSS. Next, we compute element activity by quantifying chromatin accessibility and H3K27ac ChIP-seq signal (GSM2809617 for cohesin present and GSM2809618 for cohesin absent conditions, Supplementary Data 4), both quantile-normalized to their respective observed distributions, within each candidate element. We then calculate the geometric mean of these two assays. Then, we extract element-promoter contact frequency from chromatin conformation data in the presence (ENCSR958BEA, Supplementary Data 4) or absence (ENCSR087JOM, Supplementary Data 4) of cohesin. Finally, for each element-gene pair, we calculated an ABC score that is the product of activity and contact, normalized by the product of activity and contact for all other elements within 5 Mb of that gene. For the analyses, we included all enhancer-gene pairs with an ABC score of >=0.027 (this threshold was selected to achieve a 70% recall rate based on previous K562 CRISPRi data, Supplementary data 1a,b).

ENCODE-rE2Gs a logistic regression pipeline built on top of ABC and uses the same datasets (Supplementary Data 4). For our analyses, we applied a threshold of 0.298, selected to achieve a 70% recall rate based on the same prior K562 CRISPRi data^19^ (Supplementary Data 1c,d).

Code for generating ABC and ENCODE rE2G predictions is available at (https://github.com/EngreitzLab/ENCODE_rE2G/tree/v1.0.0).

### Filtering enhancer predictions

We note that ABC produces predictions of enhancer-gene regulatory interactions for expressed genes only^19,46,78^. This is because ABC is built to predict the relative effect of an enhancer on gene expression, a quantity that is not defined if the gene is not expressed. We considered further filtering based on TPM expression levels and removal of housekeeping genes, but because the results were largely unchanged, we decided not to apply these filters to the enhancer-gene predictions in downstream analyses unless otherwise specified.

We did, however, remove any elements overlapping promoters. This resulted in 28,575 enhancer-gene pairs involving 10,393 genes with at least one ABC-predicted enhancer in the presence of cohesin and 21,523 enhancer-gene pairs involving 9489 genes with at least one ABC-predicted enhancer in the absence of cohesin. Applying the same filter to the ENCODE-rE2G predictions yielded 34,947 enhancer-gene pairs for 8698 genes in the presence and 25,813 pairs for 8134 genes in the absence of cohesin.

To assess the regulatory stability of enhancer-gene pairs, we determined whether each pair identified in the presence of cohesin was still predicted in its absence. In the ABC model, 19,292 enhancer-gene pairs were retained, with an average distance of 23.0 Kb to the TSS, while 9283 pairs were lost, showing a greater average TSS distance of 176.9 Kb. In the ENCODE-rE2G model, 23,648 pairs were retained (average distance: 28.7 Kb), and 11,299 were lost (average distance: 160.3 Kb).

For the ABC predictions in the cohesin-present condition, we further evaluated whether each predicted enhancer overlapped or was within 5 Kb of a CTCF binding site, based on ChIP-seq data ENCFF072BUT, Supplementary Data 4). We found that 13,617 predicted enhancers were within 5 Kb of a CTCF site, while 14,958 were not.

Finally, to stratify ABC predictions based on gene sensitivity to cohesin, we grouped predicted enhancer-gene pairs according to gene expression levels in the PRO-seq data (DESeq2 file from GSE106886) from the cohesin-present condition. For this study, we converted PRO-seq RPKM values from GSE106886 to TPM using the formula TPM = (RPKM / ∑RPKM) × 10⁶. We filtered for genes with TPM > 5 to ensure reliable expression-based classification. Genes were classified as downregulated as strongly cohesin-sensitive with a fold change >1.8 or moderately cohesin-sensitive with a fold change 1.4-1.8 and an adjusted *p*-value < 0.05 upon cohesin depletion. All others were categorized as non-downregulated.

Code for analyzing enhancer-gene predictions is available at https://github.com/philinegu/cohesin_CRUDO_workflow^79^.

### CRUDO target selection

To address how the quantitative contact frequency of an enhancer and its target promoter influence target gene expression levels, we examined pre-existing data on gene expression changes in the absence of cohesin^25^. We analyzed bulk RNA-seq and PRO-seq obtained from untreated and 6-h auxin treated HCT-116 RAD21-mAID-mClover. We identified ~150 candidate genes with an −0.5 log_2_ fold-change in PRO-Seq (GSE106886, Supplementary Data 4), respectively, and a basal expression of 20 transcripts per million (TPM). From these genes, we chose 5 candidate genes with diverse regulatory landscapes based on ABC predicted enhancers spanning a range of linear genomic distances.

### gRNA library design

We designed our gRNA libraries to target the transcription start sites of our genes of interest, candidate enhancers, and non-targeting negative controls. (gRNA sequences are deposited in the IGVF Data Portal under the accession numbers in Supplementary Data 3a). Candidate enhancers are 500 bp regions centered around the DNase-seq peak of ABC-predicted enhancers (CCND1 pilot CRUDO-FF screen) or within 1 Mb of that gene (CRUDO-FF screens, 5 Mb for MYC). TSSs are 500 bp centered around the DNase peak defined by PRO-seq as the TSS. We designed our gRNAs to be tiled across each element by making a list of every possible target site with an NGG PAM sequence. We then used a previously described algorithm^80^ to rank the target sites by calculating a specificity score based on potential off-target effects. We removed target sites with a score <20 and included up to 30 guides per element for our final pilot gRNA library. We included 400 non-targeting negative controls^52^. For cloning purposes, every target sequence had a ‘G’ base added to the 5’ end of the sequence, unless already present.

### CRUDO vector generation

We adapted the CROP-opti vector (Addgene, #106280) and the sgOpti vector (Addgene, #85681) to express a Blasticidin resistance cassette in place of the original Puromycin resistance cassette (CROP-opti-Blast and sgOpti-Blast). To remove the Puromycin resistance cassette we digested the vector with BsiWI (New England Biolabs) and MluI (New England Biolabs) and subsequently purified the linearized vector with 0.5X AMPure XP beads (Beckman Coulter, #A63881). We PCR-amplified the Blasticidin resistance gene from the lenti-dCas-VP64_Blast vector (Addgene, #61425), adding homology arms for Gibson Assembly. We purified the PCR-product with 1X AMPure XP beads and used Gibson Master mix (New England Biolabs) assemble the previously prepared vector backbone and purified Blasticidin PCR-amplified insert following Gibson assembly recommendations. We chemically transformed the assembled product into competent cells (New England Biolabs, #C2987H) and grew the bacteria on LB plates at 30 °C overnight. For DC-TAP-seq, we cloned a vector (based on sgOpti-Blast) that would allow us to capture guide transcripts directly with 10x beads. We added the cs1 direct capture sequence^81^ into the gRNA scaffold by digesting out the standard scaffold in sgOpti-Blast with NsiI-HF and EcoRI-HF, and then used Gibson assembly to add back in the modified scaffold. We selected single colonies to inoculate in liquid culture overnight, prepared the DNA (Machery Nagel, NucleoBond Xtra Midi EF) and screened them by enzyme digestion and Sanger sequencing (plasmid map for CROP-opti-Blast: (https://data.igvf.org/documents/4b4305ce-5844-4b84-a3c7-263a1040b050/), plasmid map for sgOpti-Blast-cs1: (https://data.igvf.org/documents/6f05cff9-a97a-45f7-9503-ebd5c44873b0/).

### gRNA library cloning

We ordered a PCR-tagged custom oligo pool (Agilent) corresponding to the gRNA library design. We PCR-amplified the gRNA pool to add homology arms for Gibson Assembly. We linearized the CROP-opti-Blast vector by digesting with BsmBI (New England Biolabs) and purified the gRNA pool and vector backbone with 1.5X and 0.7X AMPure XP beads, respectively.

For the Gibson assembly we used 500 ng of the purified vector backbone and 70 ng of the purified gRNA library with 15 μl of 2X Gibson Mix and subsequently purified the assembled product with 0.7X AMPure XP beads eluting in 15 μl H2O. We used electroporation to transform the full 15 μl of assembled product into 25 μl of electrocompetent bacteria (Endura, #60242). We plated an aliquot of the transformed cells on an LB plate in a serial dilution to estimate the number of transformed colonies. We expanded the remaining transformed cells in liquid culture to grow at 30 °C overnight. We purified the overnight cultures using an Endotoxin-Free Plasmid Midi Kit (Machery Nagel, NucleoBond Xtra Midi EF). We sequenced gRNAs to verify the library complexity.

### Cell culture

We cultivated HEK293T cells (Takara, #632180) in DMEM media (Thermo Fisher Scientific, #11995065) with 10% HIFBS (Thermo Fisher Scientific, #10082147) added. We maintained a confluency of 20-80% by splitting the cells every two days. We maintained HCT-116 cells in McCoy’s 5 A media (Thermo Fisher Scientific, #16600082) supplemented with 2mM L-glutamine (Thermo Fisher Scientific, 25030081), 100U/ml Penicillin with 100 μg/ml Streptomycin (Thermo Fisher Scientific, #15140122), and 10% HIFBS at 20-80% confluency by splitting every two days. All cells were stored in an incubator at 37 °C and 5% CO2.

### Lentivirus production & transduction

We plated 2.5 million HEK293T cells on a 10 cm dish (VWR, #10062880) and 24 h later we transfected the cells with 7.2 μg of our transfer plasmid as well as 5.4 μg of psPAX2 (Addgene, #12260) and 2.16 μg of pMD2.G (Addgene, #12259), two lentiviral co-packaging plasmids. We transfected our HEK293T cells using 1150 μl Opti-MEM (Thermo Fisher Scientific, #31985070) and 35 μl XtremeGene9 (MilliporeSigma, #6365787001) in 10 ml of DMEM with 10% HIFBS media. We combined the transfection reagents and incubated these at room temperature for 15 min, prior to adding the solution dropwise to the cells. After 16 h we exchanged the media for fresh DMEM with 10% HIFBS media. At 24 h post-transfection we harvested viral supernatant for the first time and added fresh DMEM with 10% HIFBS media to the cells. We stored the viral supernatant at 4 °C and harvested viral supernatant again at 48 h post transfection. We combined the harvested virus supernatant for each construct, filtered the supernatant using a vacuum-driven filtration system (MilliporeSigma, #SE1M003M00) and finally concentrated the virus 2X with centrifugal filters (MilliporeSigma #UFC910024). We stored the filtered and concentrated viral supernatant at 4 °C unless the time before use extended two weeks, then we divided the virus into 1 ml aliquots and snap froze at −80 °C.

We transduced HCT-116 cells in 24-well plates with a cell density of 250,000 cells per well and media supplemented with 10 μg/ml polybrene (MilliporeSigma #TR1003G). After adding the virus, we infected the cells by centrifuging at 1200 g and 32 °C for 45 min. 24 h post- transduction we removed the media containing the virus from the cells and exchanged it for fresh media supplemented with 7.5 μg/ml Blasticidin S HCI (Thermo Fisher Scientific, #A1113903) to start the 5-day selection.

### CRISPRi line generation

We generated a CRISPRi inducible HCT-116 RAD21-mAID-mClover cell line by transducing these cells with a plasmid expressing rtTA coupled to a neomycin resistance cassette (Takara, #631363). The cells were then put under selection by supplementing the media with 200 μg/ml G418 Sulfate (Thermo Fisher Scientific, #10131035). Positive cells were subsequently transduced with a construct expressing KRAB-dCas9 linked to BFP under a TRE3G promoter (Addgene, #85449). The cells were selected by fluorescent cell sorting (FACS) for BFP by activating the BFP expression with 1 μg/ml doxycycline (Thermo Fisher Scientific, #BP26531). Untransduced HCT-116 RAD21-mAID-mClover cells also treated with 1 μg/ml doxycycline were used to control for negative BFP expression during cell sorting (Supplementary Fig. 3b).

### Single-guide qPCR validation of promoter targeting guides

We cloned two non-overlapping single guides against the TSSs of the five genes we profiled by CRISPRi-FlowFISH into the CROP-opti-Blast vector (Supplementary Fig. 16, Supplementary Data 3). We then infected the single guides into CRISPRi HCT-116 RAD21-mAID-mClover cells at low MOI and selected with Blasticidin as described above. We plated gRNA-expressing stable cell lines at 500,000 cells/ml in two separate plates, both receiving 1 μg/ml doxycycline and one receiving 500 μM auxin after 18 h. 24 h after doxycycline addition, we extracted RNA from 20,000 cells per experiment in Buffer RLT (Qiagen) using Dynabeads MyOne Silane beads (Thermo Fisher), treated samples with TURBO DNase (Thermo Fisher), and cleaned again with Dynabeads MyOne Silane beads. We used AffinityScript reverse transcriptase (Agilent Technologies, Lexington, MA) and random nonamer primers to convert RNA to cDNA. We performed qPCR using SYBR Green I Master Mix (Roche) with primers for each of the genes and housekeeping control genes (Supplementary Data 6) and calculated differences using the ΔΔCT method.

### Simulation framework for CRUDO assay development

To simulate the impact of an enhancer perturbation on gene expression in the absence or presence of cohesin, we employed a computational model that considers various factors influencing gene regulation. The key parameters of the model include: i) Effect of cohesin removal on overall gene expression, ii) enhancer effect size, iii) effect of cohesin on enhancer function, iv) mRNA half-life, v) CRISPRi induction time point, vi) CRISPRi induction rate, vii) cohesin degradation induction time point, viii) cohesin degradation induction rate, and ix) the measurement timepoint. To assess whether we can distinguish enhancers that rely on cohesin for enhancer function from those that do not, we conducted simulations for distinct cohesin effects on enhancer function with varying values for CRISPRi induction time point, cohesin degradation induction time point, and rate while keeping other parameters constant. Based on these simulations, we determined that 24 h of CRISPRi treatment and degradation of cohesin for the last 6 h is sufficient to distinguish differential enhancer effects between cohesin present and absent (Supplementary Fig. 4). Code for simulations is available at (https://github.com/philinegu/cohesin_CRUDO_workflow^79^).

### CRUDO screens

We transduced doxycycline inducible CRISPRi HCT-116 RAD21-mAID-mClover cells with the gRNA library at a multiplicity of infection (MOI) of about 0.3 and a coverage of 500 transduced cells for each gRNA. After selection, transduced cells were pooled into four T-75 flasks (Thermo Fisher Scientific, #156499) for two biological replicates per condition and maintained in 2.5 μg/ml Blasticidin at a minimum coverage of 500 cells per gRNA throughout the experiment. For each target gene, the pooled cells were expanded to 12.5 million cells to ensure robust library representation across replicates. In each flask, CRISPRi expression was induced by adding 1 μg/ml doxycycline (Thermo Fisher Scientific, #BP26531). After 18 h, 500 μM auxin (Indole-3-acetic acid, MilliporeSigma, #I37505GA) was added to two of the flasks to initiate targeted degradation of the cohesin complex. After a total of 24 h, cells were harvested by aspirating the media, washing with PBS (Thermo Fisher Scientific, #10010023), treating with trypsin (Thermo Fisher Scientific, #25200114) for 2 min, resuspending in fresh media, and centrifuging at 400 g for 5 min. CRUDO screen timing was simulation-guided and optimized to detect cohesin-dependent enhancer activity (as described above).

### CRISPRi-FlowFISH screens

We performed CRISPRi-FlowFISH screens as previously described^19^. In brief, we screened two replicates of 10 M cells per gene and stained each sample with the PrimeFlow RNA Assay Kit (Thermo Fisher Scientific, #88-18005) for the respective gene with an Alexa Fluor 647 (AF647, ‘Type 1’, Supplementary Data 5) probe set and for *RPL13A* to have a positive control housekeeping gene with Alexa Fluor 488 (AF488, ‘Type 4’, Supplementary Fig. 18a, Supplementary Data 5). Using the Astrios EQ Sorter (Beckman Coulter, #B25982) we sorted the cells into six bins based on the fluorescence intensity of target genes, normalizing for staining efficiency (Supplementary Fig. 18b). We then performed reverse-crosslinking and gDNA extraction on the collected cells and sequenced the guide RNA integrations as previously described.

### CRISPRi FlowFISH processing

We processed CRISPRi-FlowFISH screens as previously described^19^. Briefly, we counted gRNAs in each bin using Bowtie^82^ to map reads to a custom index, normalized gRNA counts in each bin by library size, and then used a maximum-likelihood estimation approach to compute the effect size for each gRNA. We used the limited-memory Broyden-Fletcher-Goldfarb-Shanno algorithm (implemented in the R stats4 package) to estimate the most likely log-normal distribution that would have produced the observed guide counts, and the effect size for each gRNA is the mean of its log-normal fit divided by the average of the means from all negative-control gRNAs. As previously described, we scaled the effect size of each gRNA in a screen linearly to account for non-specific target and label probe binding. For each (FlowFISH gene, auxin treatment) pair, we performed single-guide RT-qPCR (Supplementary Fig. 16) to determine the absolute effect of knocking these genes down, and used this value to scale our FlowFISH data. Code for processing CRISPRi FlowFISH data is available at (https://github.com/EngreitzLab/crispri-flowfish).

### DC-TAP-seq

We performed a modified version of the TAP-seq protocol^47,48^. We designed a library of 347 gRNAs (gRNA sequences are deposited in the IGVF Data Portal under the accession numbers in Supplementary Data 3a) targeting a subset of the screen elements and cloned them as a pool into sgOpti-Blast-cs1 as described above. We designed DC-TAP-seq primers against 74 genes using the pipeline provided, targeting all expressed genes within 1 Mb of any of the genes tested by CRUDO-FlowFISH, as well as control genes. We prepared lentivirus with the pooled gRNA library, infected it at low MOI into CRISPRi HCT-116 RAD21-mAID-mClover cells, and treated them with doxycycline and auxin as described above. We loaded 16,000 cells per lane in two technical replicate lanes for no auxin and plus auxin conditions and performed 10x encapsulation and barcoding following the manufacturer’s instructions (10x Chromium Next GEM Single Cell 3’ GEM Kit v3.1). We then amplified our target gene panel and gRNA libraries using a mix of primers. We amplified the cDNA through sequential PCRs using a mix of DC_TAP-seq primers for dialing out target genes, and with primers from the 10x Genomics feature barcoding kit to dial out the gRNAs. We confirmed library size using an Agilent TapeStation and sequenced on a Next-seq targeting 20,000 reads per cell for the mRNA and 2000 reads per cell for the gRNA libraries. Code for processing TAP-seq data is available at (https://github.com/argschwind/CRISPRiScreen).

### CRUDO analysis pipeline

To analyze CRUDO screen data, we developed a custom analysis pipeline capable of quantifying regulatory effects and cohesin dependency across experimental conditions. The pipeline used per-guide effect size files generated from either the DC-TAP-seq or CRISPRi-FF processing workflows. Replicates were merged by averaging guide-level effects across experiments, using only gRNAs that were present in all replicates to ensure statistical validity when assessing cohesin dependency.

To compute enhancer-level effects, we averaged the replicate-averaged effect sizes of all gRNAs targeting each individual element (Supplementary Fig. 18c, d). We assessed statistical significance using a two-sided t-test comparing all gRNAs targeting a candidate element to the set of negative-control guides. Multiple testing correction was performed using the Benjamini-Hochberg procedure and used FDR < 0.05 as the threshold for calling significant regulatory effects.

We defined enhancers as elements meeting the following criteria: i) a significant effect on target gene expression (FDR < 0.05) in both replicates, ii) a > 5% reduction in gene expression with <5% difference between replicates, iii) measurement values for at least 10 gRNAs, iv) not intra-target genic and not a TSS element, and v) an H3K27ac level above the median of all tested elements.

To specifically investigate the impact of changes in 3D contact frequency on enhancer function, we filtered for enhancers with a baseline enhancer-promoter baseline Hi-C contact frequency >5 (SCALE normalized units). This ensured that only enhancers with robust measurements of initial contact frequency were analyzed for cohesin-dependent effects. To compute differences in 3D contact frequency between conditions, we first normalized contact values by scaling the auxin-treated sample (1,527,291,641 total contacts) to match the total number of contacts in the untreated condition (1,093,601,688 total contacts). The scaling factor (0.72) was calculated as the ratio of untreated to treated total contacts (1,093,601,688 / 1,527,291,641). A pseudocount of 1 was added to all contact values to avoid division by zero and stabilize fold-change calculations.

We evaluate differential enhancer effects in the presence and absence of cohesin. We performed a two-sided paired t-test for each element using replicate-averaged gRNA effect sizes of each condition. To determine cohesin dependence, we first determined the effect of each enhancer on its target gene and capped these values at zero for instances where they were less than zero (based on the assumption that an enhancer can either reduce gene expression or not have an effect). We calculated the ‘cohesin dependence’ metric on a per-element basis as the average difference in effect size between gRNAs at baseline and in the absence of cohesin, normalized by the average effect size of gRNAs in the presence of cohesin. The 95% confidence interval for the cohesin dependence was also computed on a per-element basis by using ±1.96 times the standard error of the mean, divided by the average effect size without cohesin presence.

To evaluate differential enhancer activity in the presence versus absence of cohesin, we performed a two-sided paired t-test comparing replicate-averaged guide effect sizes across conditions for each element.

To quantify “cohesin dependence,” we calculated a per-element metric as the average difference in effect size across individual gRNAs between baseline and cohesin absent conditions, normalized by the average baseline (cohesin present) effect size across those same gRNAs. Negative baseline effect sizes were capped at zero, based on the assumption that enhancer perturbation should not increase gene expression. A 95% confidence interval for cohesin dependence was computed as ±1.96 times the standard error of the delta effect (across gRNAs), normalized by the average baseline effect size.

Elements were classified as cohesin-dependent if they met three criteria: a Benjamini-Hochberg adjusted *p* value < 0.05, a replicate effect size difference (delta) <0.25, and sufficient statistical power (power >0.9) to detect 50% changes in cohesin dependence. Elements were labeled as underpowered if they failed to meet the power threshold (power <0.9) or showed inconsistent replicate measurements (delta >0.25), indicating insufficient data to confidently assess cohesin dependence; these were excluded from the cohesin-dependent category to minimize false negatives. Elements classified as not cohesin-dependent either had a non-significant adjusted *p*-value (>0.05) or did not exhibit stronger enhancer activity in the cohesin present compared to the cohesin absent condition.

To contextualize the magnitude of enhancer effects, we performed a correlation analysis between measured CRISPRi effect sizes and predicted ABC scores (Supplementary Fig. 14). Based on curve fitting, we estimated that an enhancer effect size of 15% corresponds to an ABC score of ~0.042.

Code for analyzing and visualizing cohesin CRUDO-FlowFISH or -DC-TAP-seq data is available at (https://github.com/philinegu/cohesin_CRUDO_workflow^79^).

### Linear modeling of cohesin dependence

To quantify the contribution of genomic and epigenomic features to cohesin-dependent enhancer activity, we constructed multiple linear regression models correlating cohesin dependence with genomic distance, enhancer activity, promoter activity, and changes in 3D contact frequency. Predictor variables were transformed as appropriate, including log10-transformation of the linear genomic distance to the TSS.

Initial models showed that a change in 3D contact frequency alone significantly predicted cohesin dependence (adjusted R² = 0.576), outperforming a model based solely on log10 genomic distance (adjusted R² = 0.353). All models were fit using ordinary least squares regression, and to formally compare models, we performed ANOVA F-tests for nested models.

Adding change in 3D contact frequency to the distance-only model significantly improved the fit (F(1, 27) = 17.08, *p* = 0.00031), where the numerator degrees of freedom (1) corresponds to the number of predictors added, and the denominator degrees of freedom (27) represents the amount of data left to estimate the model’s residual error after accounting for all predictors. This increased the adjusted R² from 0.35 to 0.589. In contrast, adding genomic distance to the contact-only model did not yield a significant improvement (F(1, 27) = 1.92, *p* = 0.177), with adjusted R² only increasing marginally from 0.575 to 0.589. These results highlight that changes in 3D contact frequency are a stronger and more significant predictor of cohesin dependence than genomic distance alone.

Model parameters and statistics are reported as follows:

- Model 1 (contact-only): Intercept = −0.29; contact_change = −0.795
- Model 2 (distance-only): Intercept = −0.23; distance_log10 = 0.226
- Model 3 (contact + distance): Intercept = −0.238; contact_change = −1.136; distance_log10 = −0.136

Code for CRUDO modeling is available at (https://github.com/philinegu/cohesin_CRUDO_workflow^79^).

### Reporting summary

Further information on research design is available in the Nature Portfolio Reporting Summary linked to this article.

## Supplementary information

Supplementary Information

Description of Additonal Supplementary Files

Supplementary Dataset 1

Supplementary Dataset 2

Supplementary Dataset 3

Supplementary Dataset 4

Supplementary Dataset 5

Supplementary Dataset 6

Reporting Summary

Transparent Peer Review file

## Source data

Source Data

## Data Availability

The data generated in this study have been deposited in the following databases under the specified accession codes (see Supplementary Data 4 for a detailed data summary). CRISPRi-FlowFISH data on the IGVF Data Portal: Pilot CCND1 analysis set: IGVFDS2982SJGW. Full MYC analysis set: IGVFDS4544YEQM. Full SSFA2 analysis set: IGVFDS2031CTSP. Full CCND1 analysis set: IGVFDS1569RNPD. Full FAM3C analysis set: IGVFDS5239VQTN. Full KITLG analysis set: IGVFDS6482AXWW. Accession numbers for the underlying individual data file listed in Supplementary Data 2a. CRISPRi-DC-TAP-seq data on the IGVF Data Portal: IGVFDS5308EMCG. Accession numbers for the underlying individual data file listed in Supplementary Data 2a. ENCODE Hi-C (An atlas of nuclear architecture resolves element-to-element chromatin loops in over 60 human tissues, in preparation) from the ENCODE Portal: ENCSR958BEA, ENCSR087JOM. DNase-seq from the ENCODE Portal: ENCFF166PCV, ENCFF619LVU. H3K27ac ChIP-seq data from Gene Expression Omnibus: GSM2809617, GSM2809618. RAD21 ChIP-seq data from Gene Expression Omnibus: GSM2809609, GSM2809610. CTCF ChIP-seq data from ENCODE: ENCFF072BUT. PRO-seq data from Gene Expression Omnibus: GSE106886. ENCODE-rE2G CRISPRi benchmarking set: https://github.com/EngreitzLab/CRISPR_comparison/blob/main/resources/crispr_data/EPCrisprBenchmark_combined_data.training_K562.GRCh38.tsv.gz. Source data for each figure are provided in a combined Source Data file, and the associated code and resources are available on GitHub: https://github.com/philinegu/cohesin_CRUDO_workflow^79^. Source data are provided with this paper.

## Code availability

Enhancer-gene pair predictions (computes both ABC and ENCODE rE2G scores): https://github.com/EngreitzLab/ENCODE_rE2G/tree/v1.0.0. FlowFISH processing pipeline: https://github.com/EngreitzLab/crispri-flowfish. TAP-seq primer design pipeline: https://bioconductor.org/packages/release/bioc/html/TAPseq.html. TAP-seq processing pipeline: https://github.com/argschwind/TAPseq_workflow. CRUDO analysis pipeline: https://github.com/philinegu/cohesin_CRUDO_workflow

## References

[1] Popay, T. M. & Dixon, J. R. Coming full circle: On the origin and evolution of the looping model for enhancer-promoter communication. *J. Biol. Chem.* **298**, 102117 (2022). 10.1016/j.jbc.2022.102117 PMC9283939 35691341

[2] Carter, D., Chakalova, L., Osborne, C. S., Dai, Y.-F. & Fraser, P. Long-range chromatin regulatory interactions in vivo. *Nat. Genet.* **32**, 623–626 (2002). 10.1038/ng1051 12426570

[3] Bonev, B. et al. Multiscale 3D genome rewiring during mouse neural development. *Cell* **171**, 557–572.e24 (2017). 10.1016/j.cell.2017.09.043 PMC5651218 29053968

[4] Rubin, A. J. et al. Lineage-specific dynamic and pre-established enhancer-promoter contacts cooperate in terminal differentiation. *Nat. Genet.* **49**, 1522–1528 (2017). 10.1038/ng.3935 PMC5715812 28805829

[5] Chen, H. et al. Dynamic interplay between enhancer-promoter topology and gene activity. *Nat. Genet.* **50**, 1296–1303 (2018). 10.1038/s41588-018-0175-z PMC6119122 30038397

[6] Dekker, J., Rippe, K., Dekker, M. & Kleckner, N. Capturing chromosome conformation. *Science* **295**, 1306–1311 (2002). 10.1126/science.1067799 11847345

[7] Rao, S. S. P. et al. A 3D map of the human genome at kilobase resolution reveals principles of chromatin looping. *Cell* **159**, 1665–1680 (2014). 10.1016/j.cell.2014.11.021 PMC5635824 25497547

[8] Lupiáñez, D. G. et al. Disruptions of topological chromatin domains cause pathogenic rewiring of gene-enhancer interactions. *Cell* **161**, 1012–1025 (2015). 10.1016/j.cell.2015.04.004 PMC4791538 25959774

[9] Kragesteen, B. K. et al. Dynamic 3D chromatin architecture contributes to enhancer specificity and limb morphogenesis. *Nat. Genet.* **50**, 1463–1473 (2018). 10.1038/s41588-018-0221-x PMC10154999 30262816

[10] Laugsch, M. et al. Modeling the pathological long-range regulatory effects of human structural variation with patient-specific hiPSCs. *Cell Stem Cell* **24**, 736–752.e12 (2019). 10.1016/j.stem.2019.03.004 30982769

[11] Zuin, J. et al. Nonlinear control of transcription through enhancer-promoter interactions. *Nature* **604**, 571–577 (2022). 10.1038/s41586-022-04570-y PMC9021019 35418676

[12] Rinzema, N. J. et al. Building regulatory landscapes reveals that an enhancer can recruit cohesin to create contact domains, engage CTCF sites and activate distant genes. *Nat. Struct. Mol. Biol.* **29**, 563–574 (2022). 10.1038/s41594-022-00787-7 PMC9205769 35710842

[13] van Bemmel, J. G. et al. The bipartite TAD organization of the X-inactivation center ensures opposing developmental regulation of Tsix and Xist. *Nat. Genet.* **51**, 1024–1034 (2019). 10.1038/s41588-019-0412-0 PMC6551226 31133748

[14] Wu, Y., Li, J., Bartley-Dier, E. L., Pitts, C. & Cohen, B. A. Long-range massively parallel reporter assay reveals rules of distal enhancer-promoter interactions. Preprint at *bioRxiv* 10.1101/2025.04.21.649048 (2025).

[15] Deng, W. et al. Controlling long-range genomic interactions at a native locus by targeted tethering of a looping factor. *Cell* **149**, 1233–1244 (2012). 10.1016/j.cell.2012.03.051 PMC3372860 22682246

[16] Bartman, C. R., Hsu, S. C., Hsiung, C. C.-S., Raj, A. & Blobel, G. A. Enhancer regulation of transcriptional bursting parameters revealed by forced chromatin looping. *Mol. Cell* **62**, 237–247 (2016). 10.1016/j.molcel.2016.03.007 PMC4842148 27067601

[17] Kubo, N. et al. Promoter-proximal CTCF binding promotes distal enhancer-dependent gene activation. *Nat. Struct. Mol. Biol.* **28**, 152–161 (2021). 10.1038/s41594-020-00539-5 PMC7913465 33398174

[18] Kane, L. et al. Cohesin is required for long-range enhancer action at the Shh locus. *Nat. Struct. Mol. Biol.* **29**, 891–897 (2022). 10.1038/s41594-022-00821-8 PMC7613721 36097291

[19] Fulco, C. P. et al. Activity-by-contact model of enhancer-promoter regulation from thousands of CRISPR perturbations. *Nat. Genet.* **51**, 1664–1669 (2019). 10.1038/s41588-019-0538-0 PMC6886585 31784727

[20] Fudenberg, G. et al. Formation of chromosomal domains by loop extrusion. *Cell Rep.* **15**, 2038–2049 (2016). 10.1016/j.celrep.2016.04.085 PMC4889513 27210764

[21] Sanborn, A. L. et al. Chromatin extrusion explains key features of loop and domain formation in wild-type and engineered genomes. *Proc. Natl. Acad. Sci. USA* **112**, E6456–E6465 (2015). 10.1073/pnas.1518552112 PMC4664323 26499245

[22] Busslinger, G. A. et al. Cohesin is positioned in mammalian genomes by transcription, CTCF and Wapl. *Nature* **544**, 503–507 (2017). 10.1038/nature22063 PMC6080695 28424523

[23] Haarhuis, J. H. I. et al. The cohesin release factor WAPL restricts chromatin loop extension. *Cell* **169**, 693–707.e14 (2017). 10.1016/j.cell.2017.04.013 PMC5422210 28475897

[24] Nora, E. P. et al. Targeted degradation of CTCF decouples local insulation of chromosome domains from genomic compartmentalization. *Cell* **169**, 930–944.e22 (2017). 10.1016/j.cell.2017.05.004 PMC5538188 28525758

[25] Rao, S. S. P. et al. Cohesin loss eliminates all loop domains. *Cell* **171**, 305–320.e24 (2017). 10.1016/j.cell.2017.09.026 PMC5846482 28985562

[26] Schwarzer, W. et al. Two independent modes of chromatin organization revealed by cohesin removal. *Nature* **551**, 51–56 (2017). 10.1038/nature24281 PMC5687303 29094699

[27] Wutz, G. et al. Topologically associating domains and chromatin loops depend on cohesin and are regulated by CTCF, WAPL, and PDS5 proteins. *EMBO J.* **36**, 3573–3599 (2017). 10.15252/embj.201798004 PMC5730888 29217591

[28] Benabdallah, N. S. et al. Decreased enhancer-promoter proximity accompanying enhancer activation. *Mol. Cell* **76**, 473–484.e7 (2019). 10.1016/j.molcel.2019.07.038 PMC6838673 31494034

[29] Despang, A. et al. Functional dissection of the Sox9-Kcnj2 locus identifies nonessential and instructive roles of TAD architecture. *Nat. Genet.* **51**, 1263–1271 (2019). 10.1038/s41588-019-0466-z 31358994

[30] Aljahani, A. et al. Analysis of sub-kilobase chromatin topology reveals nano-scale regulatory interactions with variable dependence on cohesin and CTCF. *Nat. Commun.* **13**, 2139 (2022). 10.1038/s41467-022-29696-5 PMC9019034 35440598

[31] Goel, V. Y., Huseyin, M. K. & Hansen, A. S. Region Capture Micro-C reveals coalescence of enhancers and promoters into nested microcompartments. *Nat. Genet.* **55**, 1048–1056 (2023). 10.1038/s41588-023-01391-1 PMC10424778 37157000

[32] Aboreden, N. G. et al. LDB1 establishes multi-enhancer networks to regulate gene expression. *Mol. Cell* **85**, 376–393.e9 (2025). 10.1016/j.molcel.2024.11.037 PMC11741933 39721581

[33] Hsieh, T.-H. S. et al. Enhancer-promoter interactions and transcription are largely maintained upon acute loss of CTCF, cohesin, WAPL or YY1. *Nat. Genet.* **54**, 1919–1932 (2022). 10.1038/s41588-022-01223-8 PMC9729117 36471071

[34] Calderon, L. et al. Cohesin-dependence of neuronal gene expression relates to chromatin loop length. *Elife* **11**, e76539 10.7554/eLife.76539 (2022). PMC9106336 35471149

[35] Cuartero, S. et al. Control of inducible gene expression links cohesin to hematopoietic progenitor self-renewal and differentiation. *Nat. Immunol.* **19**, 932–941 (2018). 10.1038/s41590-018-0184-1 PMC6195188 30127433

[36] Hansen, K. L. et al. Synergy between cis-regulatory elements can render cohesin dispensable for distal enhancer function. *Science*, **391**, eadt4221 10.1126/science.adt4221 (2026). PMC13446962 41308125

[37] Tjalsma, S. J. D. et al. Long-range enhancer-controlled genes are hypersensitive to regulatory factor perturbations. *Cell Genom.* **5**, 100778 (2025). 10.1016/j.xgen.2025.100778 PMC11960515 40010352

[38] Bohrer, C. H., Fursova, N. A. & Larson, D. R. Enhancers: a focus on synthetic biology and correlated gene expression. *ACS Synth. Biol.* **13**, 3093–3108 (2024). 10.1021/acssynbio.4c00244 39276360

[39] de Wit, E. & Nora, E. P. New insights into genome folding by loop extrusion from inducible degron technologies. *Nat. Rev. Genet.* **24**, 73–85 (2023). 10.1038/s41576-022-00530-4 36180596

[40] Razin, S. V., Ulianov, S. V. & Iarovaia, O. V. Enhancer Function in the 3D Genome. *Genes (Basel)* **14**, (2023). 10.3390/genes14061277 PMC10298269 37372457

[41] Horsfield, J. A. Full circle: a brief history of cohesin and the regulation of gene expression. *FEBS J.* **290**, 1670–1687 (2023). 10.1111/febs.16362 35048511

[42] Rittenhouse, N. L. & Dowen, J. M. Cohesin regulation and roles in chromosome structure and function. *Curr. Opin. Genet Dev.* **85**, 102159 (2024). 10.1016/j.gde.2024.102159 PMC10947815 38382406

[43] Natsume, T., Kiyomitsu, T., Saga, Y. & Kanemaki, M. T. Rapid protein depletion in human cells by auxin-inducible degron tagging with short homology donors. *Cell Rep.* **15**, 210–218 (2016). 10.1016/j.celrep.2016.03.001 27052166

[44] Lieberman-Aiden, E. et al. Comprehensive mapping of long-range interactions reveals folding principles of the human genome. *Science* **326**, 289–293 (2009). 10.1126/science.1181369 PMC2858594 19815776

[45] Chiariello, A. M., Annunziatella, C., Bianco, S., Esposito, A. & Nicodemi, M. Polymer physics of chromosome large-scale 3D organisation. *Sci. Rep.* **6**, 29775 (2016). 10.1038/srep29775 PMC4942835 27405443

[46] Gschwind, A. R. et al. An encyclopedia of human enhancer–gene regulatory interactions. *Nature*10.1038/s41586-026-10781-4. (2026). PMC13471189 42457959

[47] Ray, J. et al. An unbiased survey of distal element-gene regulatory interactions with direct-capture targeted Perturb-seq. *bioRxivorg* 10.1101/2025.09.16.676677 (2025).

[48] Schraivogel, D. et al. Targeted Perturb-seq enables genome-scale genetic screens in single cells. *Nat. Methods* **17**, 629–635 (2020). 10.1038/s41592-020-0837-5 PMC7610614 32483332

[49] Bergman, D. T. et al. Compatibility rules of human enhancer and promoter sequences. *Nature* **607**, 176–184 (2022). 10.1038/s41586-022-04877-w PMC9262863 35594906

[50] Martinez-Ara, M., Comoglio, F., van Arensbergen, J. & van Steensel, B. Systematic analysis of intrinsic enhancer-promoter compatibility in the mouse genome. *Mol. Cell* **82**, 2519–2531.e6 (2022). 10.1016/j.molcel.2022.04.009 PMC9278412 35594855

[51] Thakore, P. I. et al. Highly specific epigenome editing by CRISPR-Cas9 repressors for silencing of distal regulatory elements. *Nat. Methods* **12**, 1143–1149 (2015). 10.1038/nmeth.3630 PMC4666778 26501517

[52] Fulco, C. P. et al. Systematic mapping of functional enhancer-promoter connections with CRISPR interference. *Science* **354**, 769–773 (2016). 10.1126/science.aag2445 PMC5438575 27708057

[53] Carleton, J. B., Berrett, K. C. & Gertz, J. Multiplex enhancer interference reveals collaborative control of gene regulation by estrogen receptor α-bound enhancers. *Cell Syst.* **5**, 333–344.e5 (2017). 10.1016/j.cels.2017.08.011 PMC5679353 28964699

[54] Gasperini, M. et al. A genome-wide framework for mapping gene regulation via cellular genetic screens. *Cell* **176**, 1516 (2019). 10.1016/j.cell.2019.02.027 30849375

[55] Ulirsch, J. C. et al. Systematic functional dissection of common genetic variation affecting red blood cell traits. *Cell* **165**, 1530–1545 (2016). 10.1016/j.cell.2016.04.048 PMC4893171 27259154

[56] Wakabayashi, A. et al. Insight into GATA1 transcriptional activity through interrogation of cis elements disrupted in human erythroid disorders. *Proc. Natl. Acad. Sci. USA* **113**, 4434–4439 (2016). 10.1073/pnas.1521754113 PMC4843446 27044088

[57] Klann, T. S. et al. CRISPR–Cas9 epigenome editing enables high-throughput screening for functional regulatory elements in the human genome. *Nat. Biotechnol.* **35**, 561–568 (2017). 10.1038/nbt.3853 PMC5462860 28369033

[58] Xie, S., Duan, J., Li, B., Zhou, P. & Hon, G. C. Multiplexed engineering and analysis of combinatorial enhancer activity in single cells. *Mol. Cell* **66**, 285–299.e5 (2017). 10.1016/j.molcel.2017.03.007 28416141

[59] Qi, Z. et al. Tissue-specific gene expression prediction associates vitiligo with SUOX through an active enhancer. *bioRxiv* 10.1101/337196 (2018).

[60] Huang, J. et al. Dissecting super-enhancer hierarchy based on chromatin interactions. *Nat. Commun.* **9**, 943 (2018). 10.1038/s41467-018-03279-9 PMC5838163 29507293

[61] Xu, J. et al. Developmental control of polycomb subunit composition by GATA factors mediates a switch to non-canonical functions. *Mol. Cell* **57**, 304–316 (2015). 10.1016/j.molcel.2014.12.009 PMC4305004 25578878

[62] Oudelaar, A. M. & Higgs, D. R. Publisher Correction: The relationship between genome structure and function. *Nat. Rev. Genet.* **22**, 808 (2021). 10.1038/s41576-021-00425-w 34650258

[63] Robson, M. I., Ringel, A. R. & Mundlos, S. Regulatory landscaping: how enhancer-promoter communication is sculpted in 3D. *Mol. Cell* **74**, 1110–1122 (2019). 10.1016/j.molcel.2019.05.032 31226276

[64] Beagan, J. A. & Phillips-Cremins, J. E. On the existence and functionality of topologically associating domains. *Nat. Genet.* **52**, 8–16 (2020). 10.1038/s41588-019-0561-1 PMC7567612 31925403

[65] Gabriele, M. et al. Dynamics of CTCF- and cohesin-mediated chromatin looping revealed by live-cell imaging. *Science* **376**, 496–501 (2022). 10.1126/science.abn6583 PMC9069445 35420890

[66] Narendra, V. et al. CTCF establishes discrete functional chromatin domains at the Hox clusters during differentiation. *Science* **347**, 1017–1021 (2015). 10.1126/science.1262088 PMC4428148 25722416

[67] Paliou, C. et al. Preformed chromatin topology assists transcriptional robustness of during limb development. *Proc. Natl. Acad. Sci. Usa.* **116**, 12390–12399 (2019). 10.1073/pnas.1900672116 PMC6589666 31147463

[68] Eder, M., Moene, C. J. I., Dauban, L., Leemans, C. & van Steensel, B. Functional maps of a genomic locus reveal confinement of an enhancer by its target gene. *bioRxiv* 10.1101/2024.08.26.609360 (2024). 2024.08.26.609360. PMC7618358 40966339

[69] Thiecke, M. J. et al. Cohesin-dependent and -independent mechanisms mediate chromosomal contacts between promoters and enhancers. *Cell Rep.* **32**, 107929 (2020). 10.1016/j.celrep.2020.107929 PMC7383238 32698000

[70] Stik, G. et al. CTCF is dispensable for immune cell transdifferentiation but facilitates an acute inflammatory response. *Nat. Genet.* **52**, 655–661 (2020). 10.1038/s41588-020-0643-0 32514124

[71] Sherr, C. J. et al. D-type cyclins. *Trends Biochem. Sci.* **20**, 187–190 (1995). 10.1016/s0968-0004(00)89005-2 7610482

[72] Magiera, K. et al. Lithocholic acid hydroxyamide destabilizes cyclin D1 and induces G/G arrest by inhibiting deubiquitinase USP2a. *Cell Chem. Biol.* **24**, 458–470.e18 (2017). 10.1016/j.chembiol.2017.03.002 PMC5404848 28343940

[73] Gennaro, V. J. et al. Control of CCND1 ubiquitylation by the catalytic SAGA subunit USP22 is essential for cell cycle progression through G1 in cancer cells. *Proc. Natl. Acad. Sci. USA* **115**, E9298–E9307 (2018). 10.1073/pnas.1807704115 PMC6176615 30224477

[74] Kim, K. L. et al. Dissection of a CTCF topological boundary uncovers principles of enhancer-oncogene regulation. *Mol. Cell*10.1016/j.molcel.2024.02.007 (2024). PMC10997458 38452764

[75] Brandão, H. B., Gabriele, M. & Hansen, A. S. Tracking and interpreting long-range chromatin interactions with super-resolution live-cell imaging. *Curr. Opin. Cell Biol.* **70**, 18–26 (2021). 10.1016/j.ceb.2020.11.002 PMC8364313 33310227

[76] Zhou, H. Y. et al. A Sox2 distal enhancer cluster regulates embryonic stem cell differentiation potential. *Genes Dev.* **28**, 2699–2711 (2014). 10.1101/gad.248526.114 PMC4265674 25512558

[77] Alexander, J. M. et al. Live-cell imaging reveals enhancer-dependent transcription in the absence of enhancer proximity. *Elife* **8**, e41769 10.7554/eLife.41769 (2019). PMC6534382 31124784

[78] Nasser, J. et al. Genome-wide enhancer maps link risk variants to disease genes. *Nature* **593**, 238–243 (2021). 10.1038/s41586-021-03446-x PMC9153265 33828297

[79] Guckelberger, P. et al. *Philinegu/cohesin_CRUDO_workflow: CRUDO Workflow v0.0*. *Zenodo*, 10.5281/ZENODO.21269007 (2026).

[80] Hsu, P. D. et al. DNA targeting specificity of RNA-guided Cas9 nucleases. *Nat. Biotechnol.* **31**, 827–832 (2013). 10.1038/nbt.2647 PMC3969858 23873081

[81] Replogle, J. M. et al. Combinatorial single-cell CRISPR screens by direct guide RNA capture and targeted sequencing. *Nat. Biotechnol.* **38**, 954–961 (2020). 10.1038/s41587-020-0470-y PMC7416462 32231336

[82] Langmead, B., Trapnell, C., Pop, M. & Salzberg, S. L. Ultrafast and memory-efficient alignment of short DNA sequences to the human genome. *Genome Biol.* **10**, R25 (2009). 10.1186/gb-2009-10-3-r25 PMC2690996 19261174

