# Supplementary material for: Quantitative changes in 3D contact predict changes in enhancer-promoter regulation upon cohesin depletion
Source: Nat Commun. 2026 Sep 29;17:11218. doi: 10.1038/s41467-026-77582-1 (PMC13720136; doi:10.1038/s41467-026-77582-1)
Supplement: Supplementary file 2 [file 41467_2026_77582_MOESM1_ESM.pdf]

# Quantitative changes in 3D contact predict changes in enhancer-promoter regulation upon cohesin depletion

## Supplementary Information File

## Supplementary Note 1: Alternative interpretations and model considerations

Our CRUDO data across 30 enhancers in 5 genomic loci support a relationship between 3D contacts and enhancer function. However, this does not exclude contributions by other factors or alternative mechanisms, which could tune this relationship and also be responsive to cohesin depletion. Specific possibilities to consider include (i) possible changes in promoter responsiveness, (ii) possible interactions among nearby enhancers, and (iii) changes in intrinsic enhancer activity.

(i) One possibility to consider is whether the overall responsiveness of a promoter to all of its distal enhancers might change upon cohesin depletion. If promoter responsiveness changed upon cohesin removal, the promoter's sensitivity to enhancer signals would be altered. This could produce gene expression changes that do not directly correspond to changes in enhancer-promoter contacts or enhancer activity. For example, an increase in promoter responsiveness could amplify residual enhancer signals or other regulatory inputs, potentially buffering the effects of reduced enhancer contacts. Conversely, a decrease in promoter responsiveness might further diminish gene expression, independent of changes in enhancer function.

Instead, our data show a strong correlation between reductions in enhancer-promoter 3D contacts and decreases in enhancer effect on gene expression, supporting a model in which promoter responsiveness remains largely stable. We note that we do observe that three gene promoters have significantly positive cohesin dependence, as defined by the relative contribution of that element to overall gene expression, but this does not necessarily indicate a change in responsiveness. Rather, this is consistent with promoters having similar autonomous output on gene expression across the two conditions, and thus contributing to a relatively larger proportion of gene expression in the cohesin absent condition (where enhancer inputs are decreased and total expression is lower).

However, (ii) Another possibility to consider is how CRUDO results should be interpreted if there were functional interactions between nearby enhancers, either to create redundancy or super-additivity. Because each cell in our CRISPRi screen receives only a single gRNA, we cannot directly assess functional redundancy between enhancers. However, if two enhancers were completely redundant, targeting one would yield no change in baseline expression and thus it would not be classified as a functional enhancer, and consequently not be evaluated for cohesin dependence. For instance, if two hypothetical enhancers, E1 and E2, were considered, where E1 is not cohesin-dependent but E2 is, and the two act redundantly, targeting E1 alone would not affect expression at baseline, and E1 would not be identified as a functional enhancer in our screen (Supplementary Fig. 17).

It was recently shown that enhancers are more likely to act synergistically with super-additive contributions to gene expression, than to buffer one another[1]. Some of this synergy may arise from direct 3D contacts between enhancers (Supplementary Fig. 17). In certain cases, enhancer-enhancer communication could buffer the effects of disrupted enhancer-promoter looping[2], thereby contributing to the observed variation in cohesin dependence.

(iii) Finally, intrinsic enhancer activity could change in response to cohesin depletion. To investigate this, we analyzed H3K27ac ChIP-seq data before and after cohesin depletion, and found that most elements do not change substantially in their quantitative H3K27ac signals, albeit with some exceptions. However, because H3K27ac is an imperfect proxy for enhancer activity and can reflect signals from nearby regions, we cannot exclude that changes in activity also contribute in some cases. For example, at certain distal enhancers, we noticed that H3K27ac increased upon depletion of cohesin (Supplementary Figure 12e,f), consistent with strengthening of some compartmentalization effects previously observed[3]. Moreover, recent studies indicate that H3K27ac can reflect not only intrinsic enhancer activity but also signals from nearby regulatory elements (e.g., within ~10Kb[1]). For example, in the CCND1 locus, enhancers E1 and E2 are located ~5Kb from the TSS, and all three elements exhibit decreased H3K27ac after cohesin loss. This may reflect reduced transcription and acetylation at the TSS, potentially driven by activity loss at a more distal enhancer such as E1. Future experiments using reporter assays or targeted perturbations will be necessary to disentangle such higher-order enhancer-enhancer effects.

## Supplementary Figures

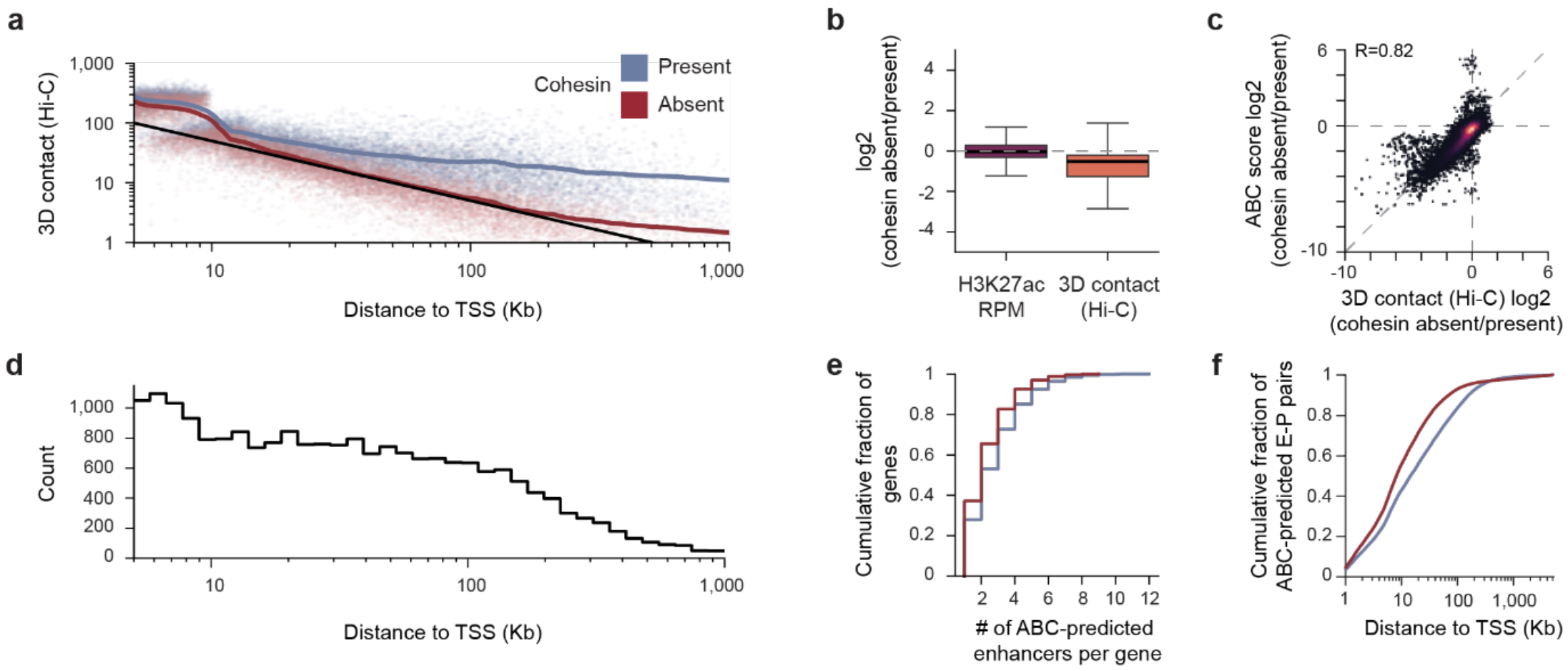


**Supplementary Fig. 1 Intrinsic enhancer activity and contact frequency in the ABC model**

**a,** Enhancer-promoter 3D contact frequency (Hi-C SCALE-normalized counts, 5Kb resolution[4]) in the presence (blue, no auxin, Hi-C depth = 1.1 billion contacts) or absence (red, plus 6 hours auxin, Hi-C depth = 1.5 billion contacts) of cohesin, as a function of distance from enhancer to target promoter. Scatter plot represents all enhancer-gene regulatory interactions predicted by ABC in the cohesin present condition. Lines: Rolling average for all enhancer-gene regulatory interactions predicted by ABC smoothed over 1,000 data point windows. Black line: Enhancer-promoter contact frequency predicted by the inverse power law model. **b,** Changes in H3K27ac levels and 3D contact frequency (Hi-C SCALE-normalized counts, 5Kb resolution) between cohesin absent and present for all enhancer-promoter regulatory interactions predicted by ABC (n=28,575). Boxes: median and interquartile range. Whiskers: full distribution, except for points determined "outliers" using the interquartile range. **c,** Change in ABC score between cohesin present and absent in $log_2$ as a function of the change in Hi-C contact frequency (Hi-C counts at a 5Kb resolution) between cohesin present and absent in $log_2$ for all enhancer-promoter regulatory interactions predicted by ABC (n=28,575). **d,** Considering genes that have at least one predicted ABC enhancer, count of ABC-predicted enhancer-promoter pairs in HCT116 cells in the presence of cohesin (n=28,575) as a function of distance from enhancer to target promoter. **e,** Considering genes that have at least one predicted ABC enhancer, cumulative distribution plot of the number of ABC enhancers per gene in the presence of cohesin (blue, n=28,575) and absence of cohesin (red, n=21,523). **f,** Considering genes that have at least one predicted ABC enhancer, cumulative distribution plot of the distance from enhancer to target gene TSS for each ABC-predicted enhancer-promoter pair in HCT116 cells in the presence of cohesin (blue, n=28,575) and the absence of cohesin (red, n=21,523).

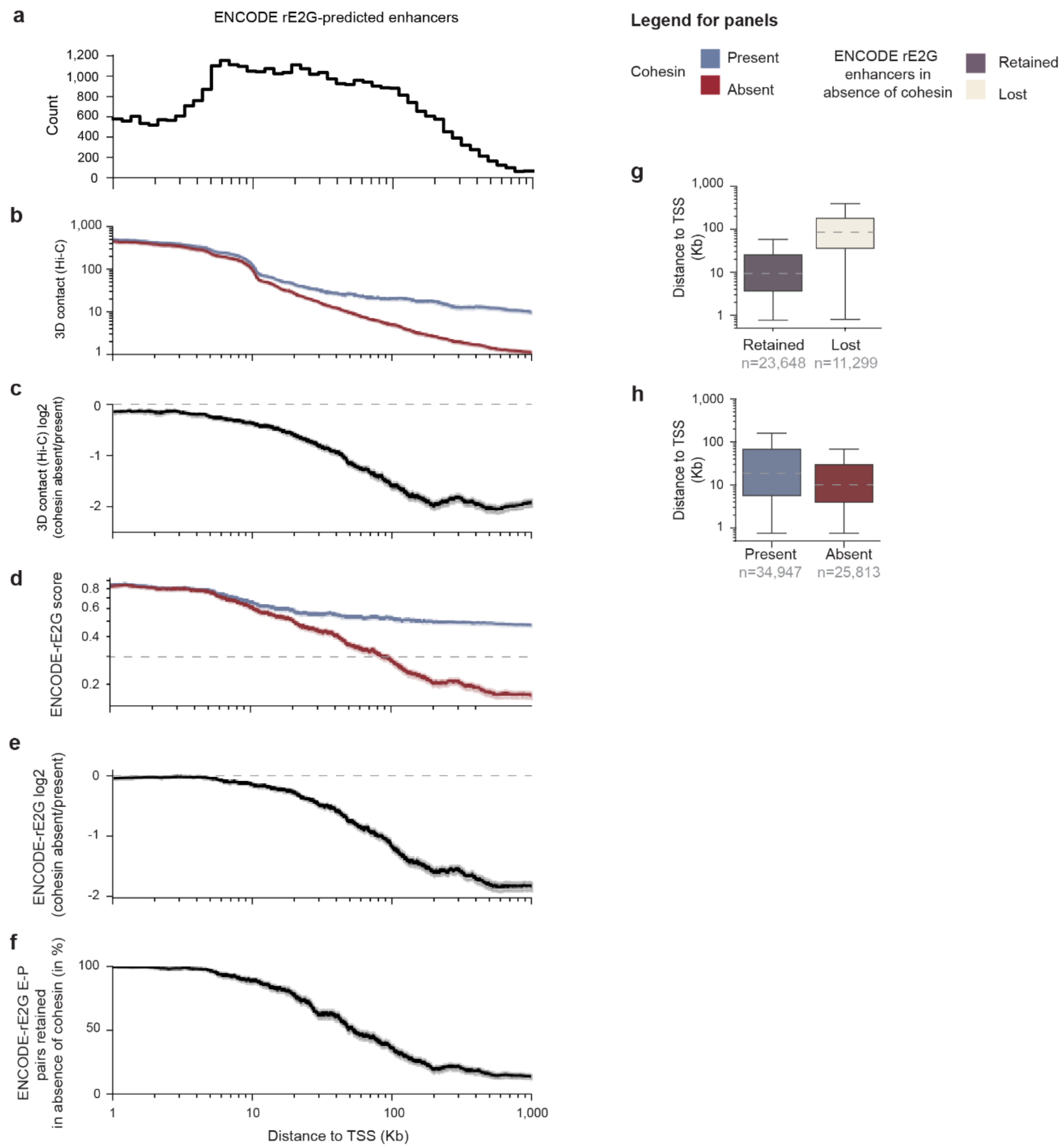


**Supplementary Fig. 2 Summary of ENCODE-rE2G enhancer predictions for the HCT116 cell line in the presence and absence of cohesin.**

**a**, Considering genes that have at least one predicted ENCODE-rE2G enhancer, count of ENCODE-rE2G-predicted enhancer-promoter pairs in HCT116 cells in the presence of cohesin (n=34,947), as a function of distance from enhancer to target promoter. **b,** Similar to **a**, showing ENCODE-rE2G score in the presence (blue, no auxin) or absence (red, plus 6 hours auxin) of cohesin, as a function of distance from enhancer to target promoter. Lines: Rolling average for all enhancer-gene regulatory interactions predicted by ENCODE-rE2G in the cohesin present condition (n=34,947) smoothed over 1,000 data point windows. Shading: 95% c.i. of the rolling average. **c,** Similar to **a**, showing enhancer-promoter 3D contact frequency (Hi-C SCALE-normalized counts, 5Kb resolution[4]) in the presence (blue, no auxin , Hi-C depth = 1.1 billion contacts) or absence (red, plus 6 hours auxin, Hi-C depth = 1.5 billion contacts) of cohesin, as a function of distance from enhancer to target promoter. Lines: Rolling average for all enhancer-gene regulatory interactions predicted by ENCODE-rE2G in the cohesin present condition (n=34,947) smoothed over 1,000 data point windows. Shading: 95% confidence interval (c.i.) of the rolling average. **d,** Similar to **a**, showing a $\log_2$ fold-change in Hi-C contact frequency between absence and presence of cohesin as a function of distance. Black line: Rolling average. Gray shading: 95% c.i. of the rolling average. **e,** Similar to **a**, showing a $\log_2$ fold-change in ENCODE-rE2G score between the absence and presence of cohesin as a function of distance. **f,** Similar to **a**, showing percentage of

enhancer-promoter regulatory interactions predicted by ENCODE-rE2G in the cohesin present condition that also pass the ENCODE-rE2G score threshold (0.298) in the absence of cohesin (retained enhancers, n=23,648), as a function of distance. **g,** Distribution of distances from enhancer to target promoter for all enhancer-promoter ENCODE-rE2G-predicted regulatory interactions retained (dark gray, n=23,648) or lost (cream, n=11,299) in the absence of cohesin. **h,** Distribution of distances from enhancer to target promoter for all enhancer-promoter regulatory interactions predicted by ENCODE-rE2G in the presence (blue, n=34,947) or absence of cohesin (red, n=25,813).

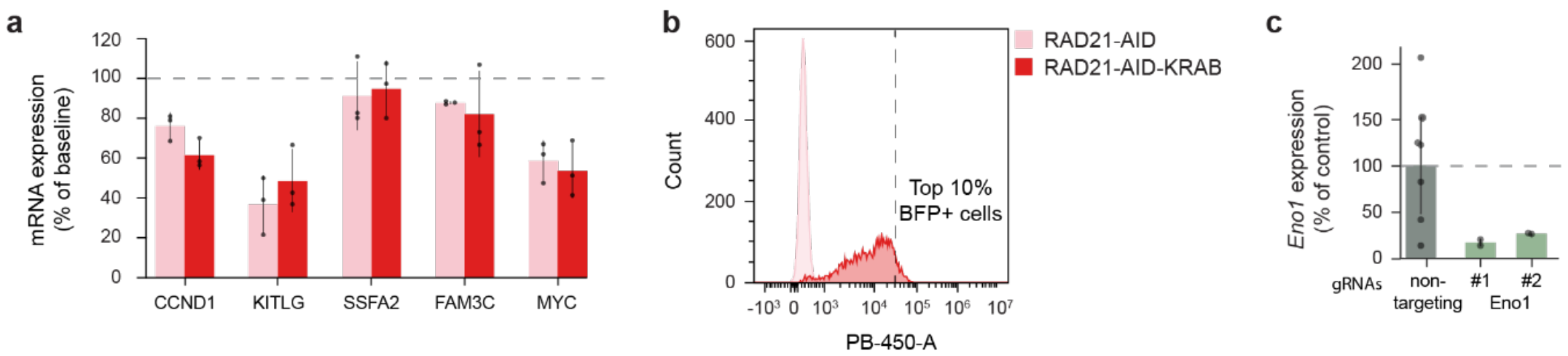


**Supplementary Fig. 3 Validating the cohesin depletion cell line additionally expressing the CRISPRi machinery.**

**a,** mRNA expression levels in the absence of cohesin (6h of auxin treatment) as measured by qPCR in the HCT116 cohesin depletion line (rose) and the further engineered cohesin depletion with CRISPRi (red) for the CRUDO-FF screen genes. Error bars represent the 95% c.i.s. **b**, FACS showing dox inducibility of HCT-116 RAD21-mAiD-mClover-KRAB-dCAS9 after sorting but before the screen. HCT-116 RAD21-mAiD-mClover control cells (rose) and HCT-116 RAD21-mAiD-mClover-KRAB-dCAS9 (red) cells were treated with Doxycycline for 24h and the analyzed. Live cells were selected using FSC-A/SSC-A, doublets were similarly removed with FSC-H/FSC-W and SSC-H/SSC-W, and the top 10% of the HCT-116 RAD21-mAiD-mClover-KRAB-dCAS9 BFP-positive peak (PB450-A) were sorted. **c,** qPCR for *ENO1* in the HCT116 cohesin depletion CRISPRi line for negative control guides (non-targeting) and two ENO1-targeting guides (ENO1-1, ENO1-2). Error bars represent the 95% c.i..

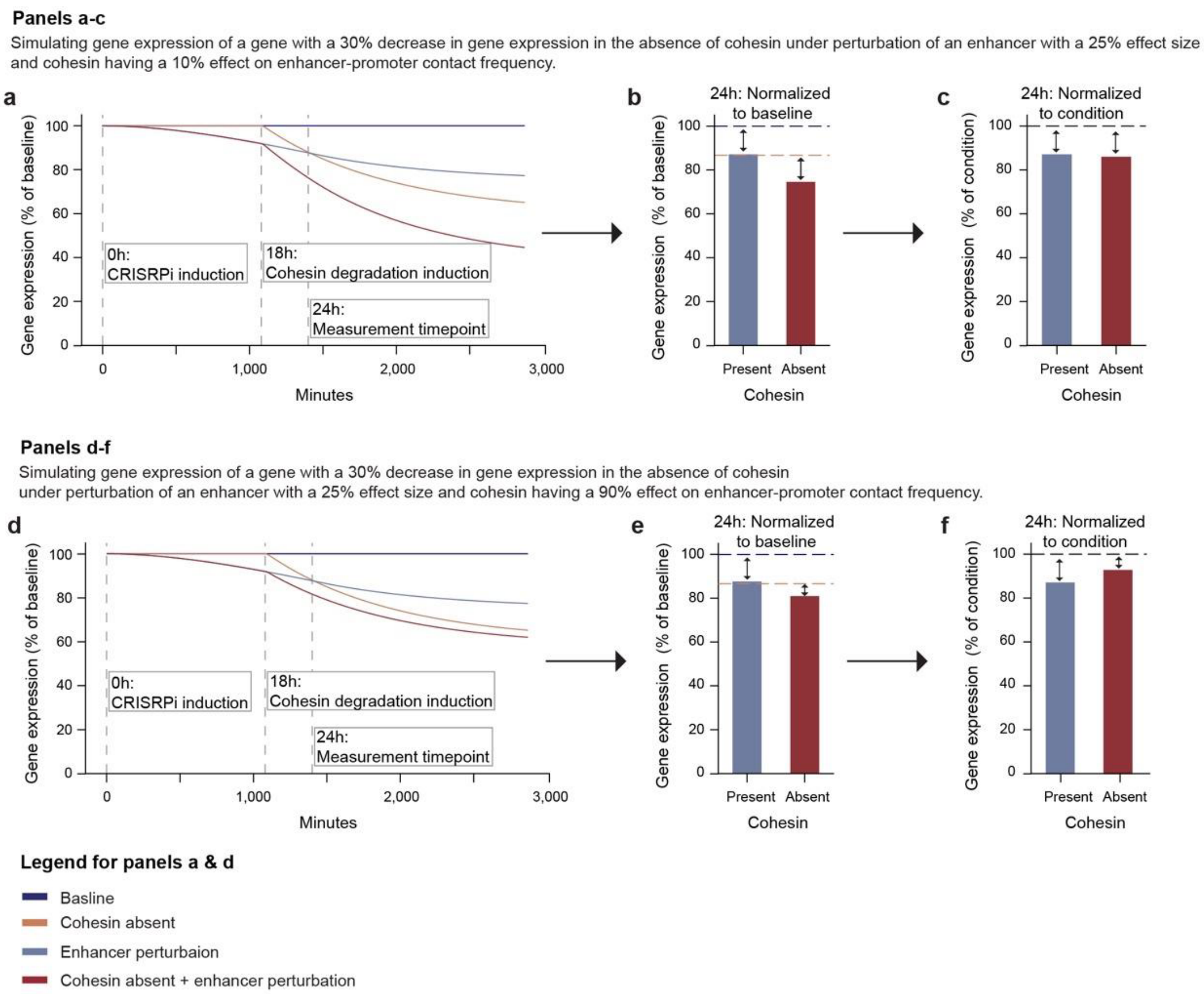


**Supplementary Fig. 4 Simulation of cohesin depletion and enhancer perturbation treatment**

**a**, 48h gene expression simulation for four conditions - Baseline (dark blue), Cohesin absent (peach), Enhancer perturbation (light blue), and Cohesin absent plus enhancer perturbation (red) - under perturbation of an enhancer induced at 0h with a 25% effect on its target gene that has an mRNA half-life of 6 hours and loses 30% of gene expression in the absence of cohesin after 6h with a 10% effect of cohesin on enhancer-promoter contact frequency. 24h zoom in of the perturbation as percent of the baseline (**b**) and the respective cohesin condition (**c**). **d,** Similar to **a**, but with a 90% effect of cohesin on enhancer-promoter contact. frequency. **e**, Similar to **b**, with a 90% effect of cohesin on enhancer-promoter contact frequency. **f,** Similar to **c**, with a 90% effect of cohesin on enhancer-promoter contact frequency.

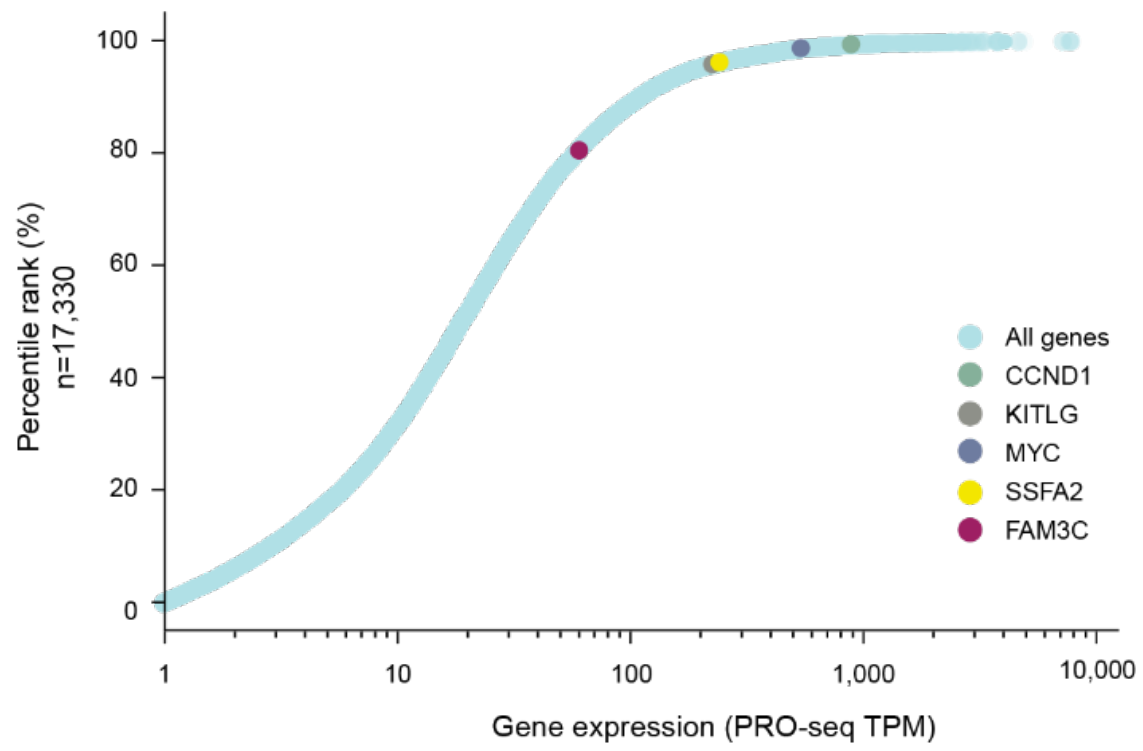


**Supplementary Fig. 5 Gene ranking by PRO-seq expression level.**

Genes (n =17,330 TPM > 1) are ranked from highest to lowest based on PRO-seq expression levels (TPM) in control conditions. Colors represent individual genes: CCND1 (sage), KITLG (gray), SSFA2 (yellow), FAM3C (purple), and MYC (blue). Light blue indicates all other genes.

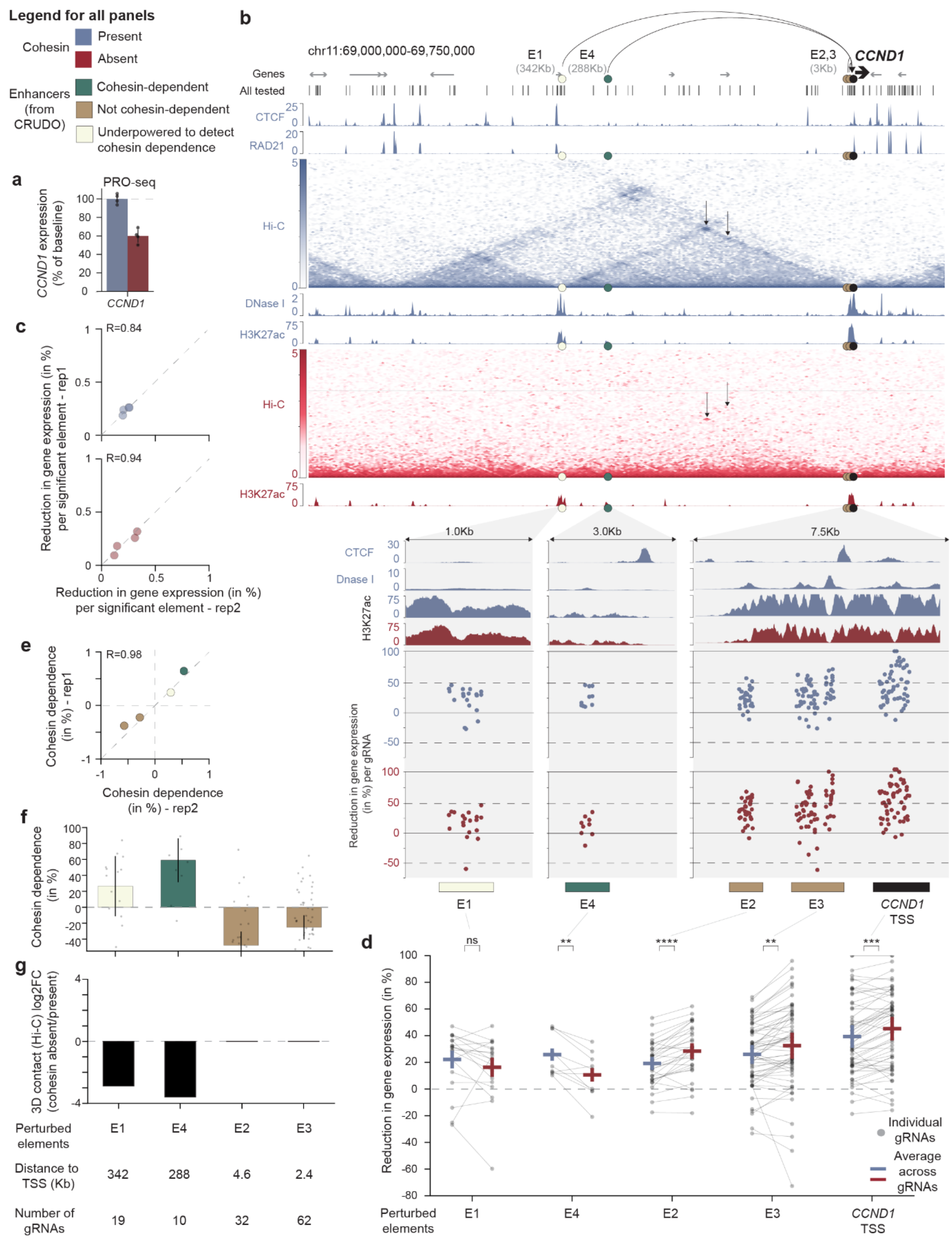


**Supplementary Fig. 6 Browser snapshot of the CCND1 locus with CRUDO-FF screen effects**

**a,** *CCND1* transcript levels in the presence (blue, 6h of DMSO treatment) or absence (red, 6h of auxin treatment) of cohesin, as measured by PRO-seq. Error bars: 95% c.i. across 4 biological replicates. **b,** *CCND1* locus showing DNase I hypersensitivity, CTCF ChIP-seq, RAD21 ChIP-seq, SCALE-normalized Hi-C at 5Kb resolution (Hi-C depth = 1.1 (no auxin) and 1.5 (plus 6 hours auxin) billion contacts)[4], and H3K27ac ChIP-seq in the presence (blue) and/or absence (red) of cohesin. Bars at the top shows locations of all elements tested in the CRUDO-FF experiment. Zoom-ins show all elements that had a significant effect on *CCND1* expression in cohesin present condition. Bars at bottom show genomic locations of elements identified in the CRUDO-FF screen (teal: cohesin-dependent enhancers; taupe: not

cohesin-dependent enhancers; ivory: underpowered to detect cohesin dependence enhancers; black: TSS). Dots show the measured effects on *CCND1* expression for individual gRNAs targeting each element (% reduction in gene expression, normalized to the mean of all non-targeting gRNAs within each condition). **c**, Scatterplot shows correlation of effects on gene expression between two biological replicate experiments in the cohesin present (blue, top panel) and cohesin absent (red, bottom panel) conditions. Dots: all 4 elements that had significant effects on gene expression in the cohesin present condition. **d,** CRUDO-FF measured effect sizes on *CCND1* expression for individual gRNAs in the presence (blue) or absence of cohesin (red). Horizontal lines and error bars: mean (+/- 95% c.i.) of effect sizes for each element. ns: $P_{BenjaminiHochberg} > 0.05$, **: $P_{BenjaminiHochberg} < 0.01$, ***: $P_{BenjaminiHochberg} < 0.001$, ****: $P_{BenjaminiHochberg} < 0.0001$ (gRNA-based paired 2-sample t-test). **e,** Similar to panel **c,** showing correlation of cohesin dependence between two biological replicate experiments. **f**, The level of cohesin dependence for each element, describing the change in the effect of an element on gene expression between the cohesin present versus absent conditions. Bar: mean across gRNAs for each element. Error bars: 95% c.i.. **g**, $Log_2$ fold-change in 3D contact frequencies (Hi-C SCALE-normalized counts, 5Kb resolution, Hi-C depth = 1.1 (no auxin) and 1.5 (plus 6 hours auxin) billion contacts) between the absence and presence of cohesin.

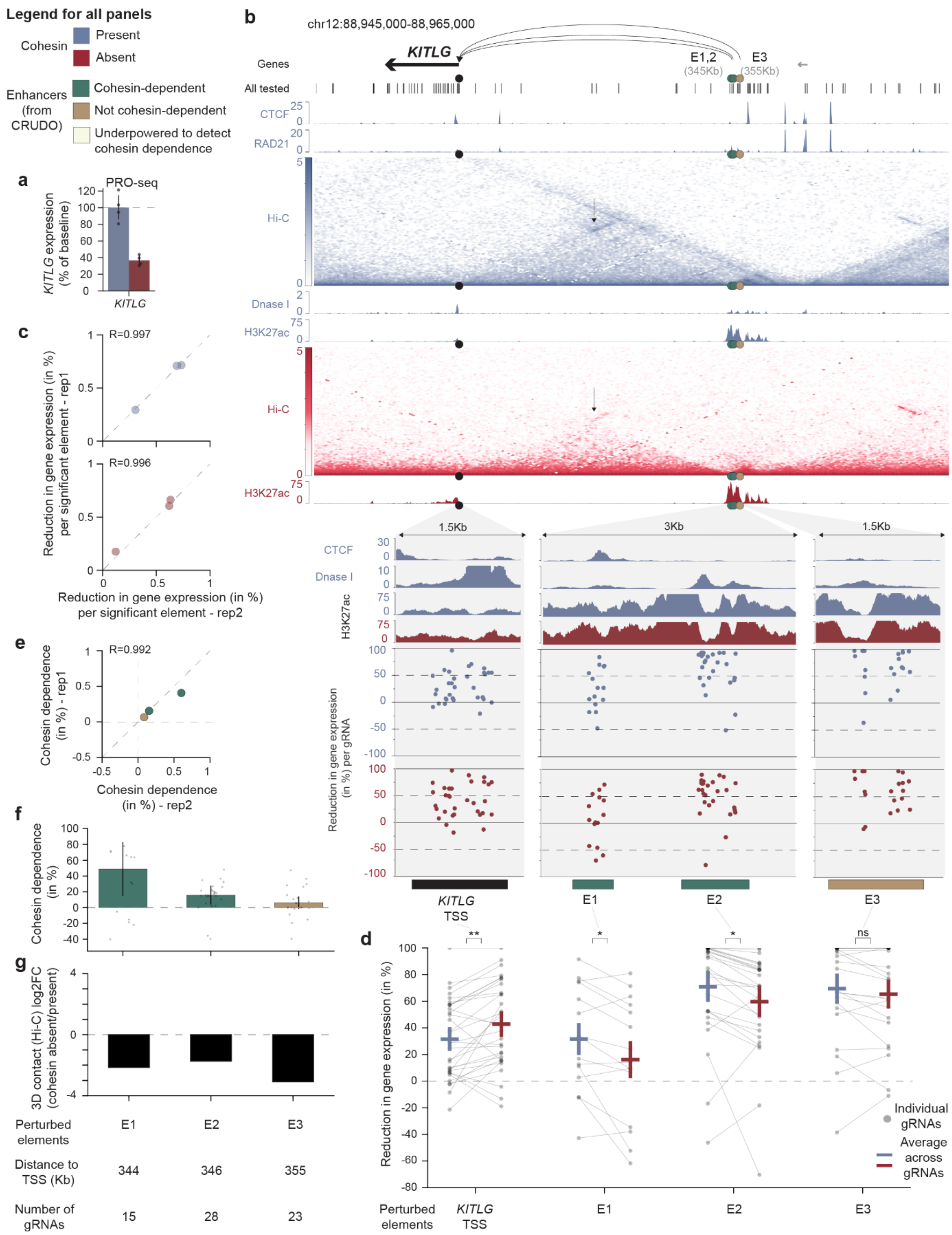


**Supplementary Fig. 7 Browser snapshot of the KITLG locus with CRUDO-FF screen effects**

**a,** *KITLG* transcript levels in the presence (blue, 6h of DMSO treatment) or absence (red, 6h of auxin treatment) of cohesin, as measured by PRO-seq. Error bars: 95% c.i. across 4 biological replicates. **b,** *KITLG* locus showing DNase I hypersensitivity, CTCF ChIP-seq, RAD21 ChIP-seq, SCALE-normalized Hi-C at 5Kb resolution (Hi-C depth = 1.1 (no auxin) and 1.5 (plus 6 hours auxin) billion contacts)[4], and H3K27ac ChIP-seq in the presence (blue) and/or absence (red) of cohesin. Bars at the top shows locations of all elements tested in the CRUDO-FF experiment. Zoom-ins show all elements that had a significant effect on *KITLG* expression in cohesin present condition. Bars at bottom show genomic locations of elements identified in the CRUDO-FF screen (teal: cohesin-dependent enhancers; taupe: not

cohesin-dependent enhancers; ivory: underpowered to detect cohesin dependence enhancers; black: TSS). Dots show the measured effects on *KITLG* expression for individual gRNAs targeting each element (% reduction in gene expression, normalized to the mean of all non-targeting gRNAs within each condition). **c**, Scatterplot shows correlation of effects on gene expression between two biological replicate experiments in the cohesin present (blue, top panel) and cohesin absent (red, bottom panel) conditions. Dots: all 3 elements that had significant effects on gene expression in the cohesin present condition. **d,** CRUDO-FF measured effect sizes on *KITLG* expression for individual gRNAs in the presence (blue) or absence of cohesin (red). Horizontal lines and error bars: mean (+/- 95% c.i.) of effect sizes for each element. ns: $P_{BenjaminiHochberg} > 0.05$, $*P_{BenjaminiHochberg} < 0.05$,(gRNA-based paired 2-sample t-test). **e,** Similar to panel **c,** showing correlation of cohesin dependence between two biological replicate experiments. **f**, The level of cohesin dependence for each element, describing the change in the effect of an element on gene expression between the cohesin present versus absent conditions. Bar: mean across gRNAs for each element. Error bars: 95% c.i.. **g**, $Log_2$ fold-change in 3D contact frequencies (Hi-C SCALE-normalized counts, 5Kb resolution, Hi-C depth = 1.1 (no auxin) and 1.5 (plus 6 hours auxin) billion contacts) between the absence and presence of cohesin.

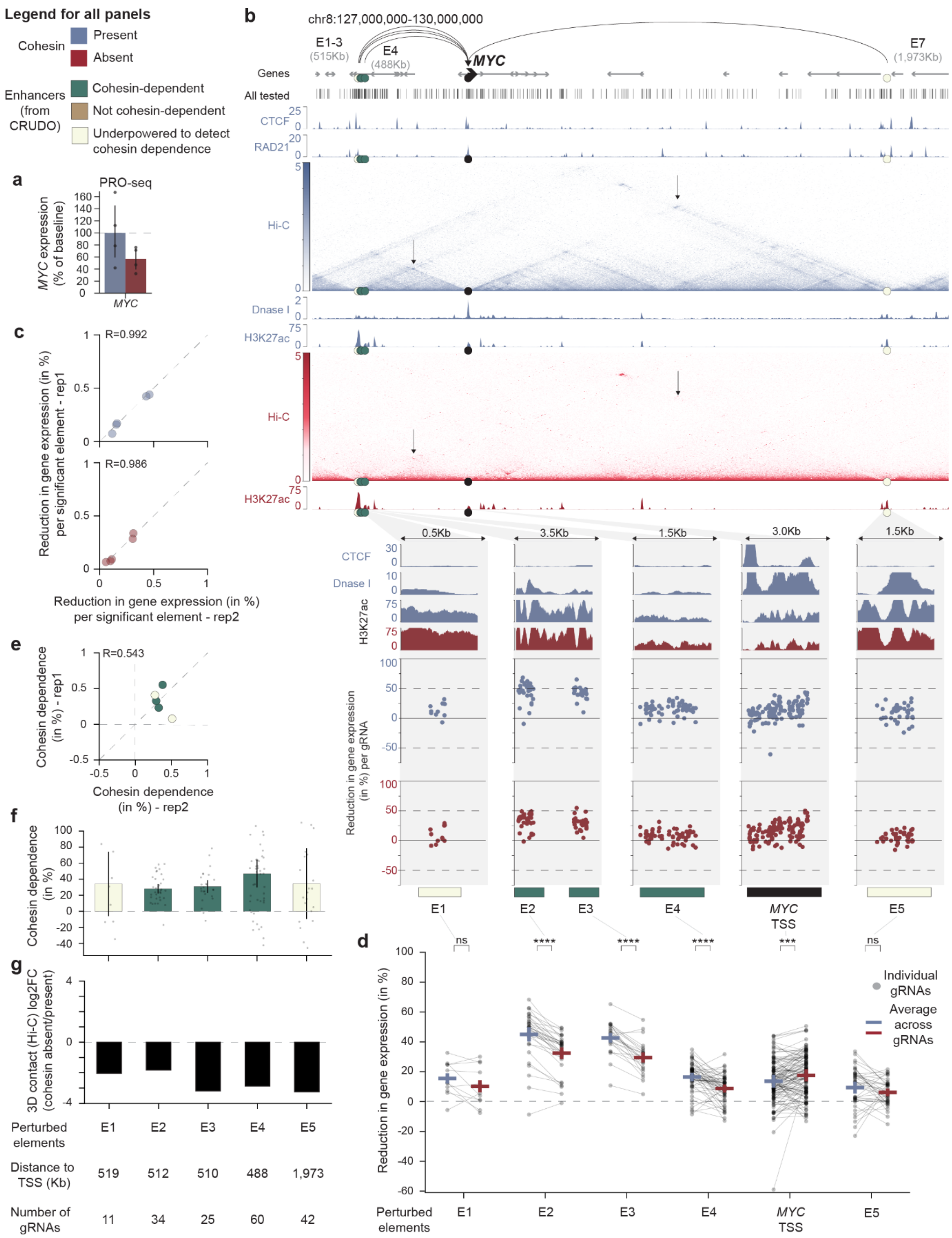


**Supplementary Fig. 8 Browser snapshot of the MYC locus with CRUDO-FF screen effects**

**a,** *MYC* transcript levels in the presence (blue, 6h of DMSO treatment) or absence (red, 6h of auxin treatment) of cohesin, as measured by PRO-seq. Error bars: 95% c.i. across 4 biological replicates. **b,** *MYC* locus showing DNase I hypersensitivity, CTCF ChIP-seq, RAD21 ChIP-seq, SCALE-normalized Hi-C at 5Kb resolution (Hi-C depth = 1.1 (no auxin) and 1.5 (plus 6 hours auxin) billion contacts)[4], and H3K27ac ChIP-seq in the presence (blue) and/or absence (red) of cohesin. Bars at the top shows locations of all elements tested in the CRUDO-FF experiment. Zoom-ins show all elements that had a significant effect on *MYC* expression in cohesin present condition. Bars at bottom show genomic locations of elements identified in the CRUDO-FF screen (teal: cohesin-dependent enhancers; taupe: not

cohesin-dependent enhancers; ivory: underpowered to detect cohesin dependence enhancers; black: TSS). Dots show the measured effects on *MYC* expression for individual gRNAs targeting each element (% reduction in gene expression, normalized to the mean of all non-targeting gRNAs within each condition). **c**, Scatterplot shows correlation of effects on gene expression between two biological replicate experiments in the cohesin present (blue, top panel) and cohesin absent (red, bottom panel) conditions. Dots: all 5 elements that had significant effects on gene expression in the cohesin present condition. **d,** CRUDO-FF measured effect sizes on *MYC* expression for individual gRNAs in the presence (blue) or absence of cohesin (red). Horizontal lines and error bars: mean (+/- 95% c.i.) of effect sizes for each element. ns: $P_{BenjaminiHochberg} > 0.05$, *: $P_{BenjaminiHochberg} < 0.05$, ***: $P_{BenjaminiHochberg} < 0.001$, ****: $P_{BenjaminiHochberg} < 0.0001$ (gRNA-based paired 2-sample t-test). **e,** Similar to panel **c,** showing correlation of cohesin dependence between two biological replicate experiments. **f**, The level of cohesin dependence for each element, describing the change in the effect of an element on gene expression between the cohesin present versus absent conditions. Bar: mean across gRNAs for each element. Error bars: 95% c.i.. **g**, $Log_2$ fold-change in 3D contact frequencies (Hi-C SCALE-normalized counts, 5Kb resolution, Hi-C depth = 1.1 (no auxin) and 1.5 (plus 6 hours auxin) billion contacts) between the absence and presence of cohesin.

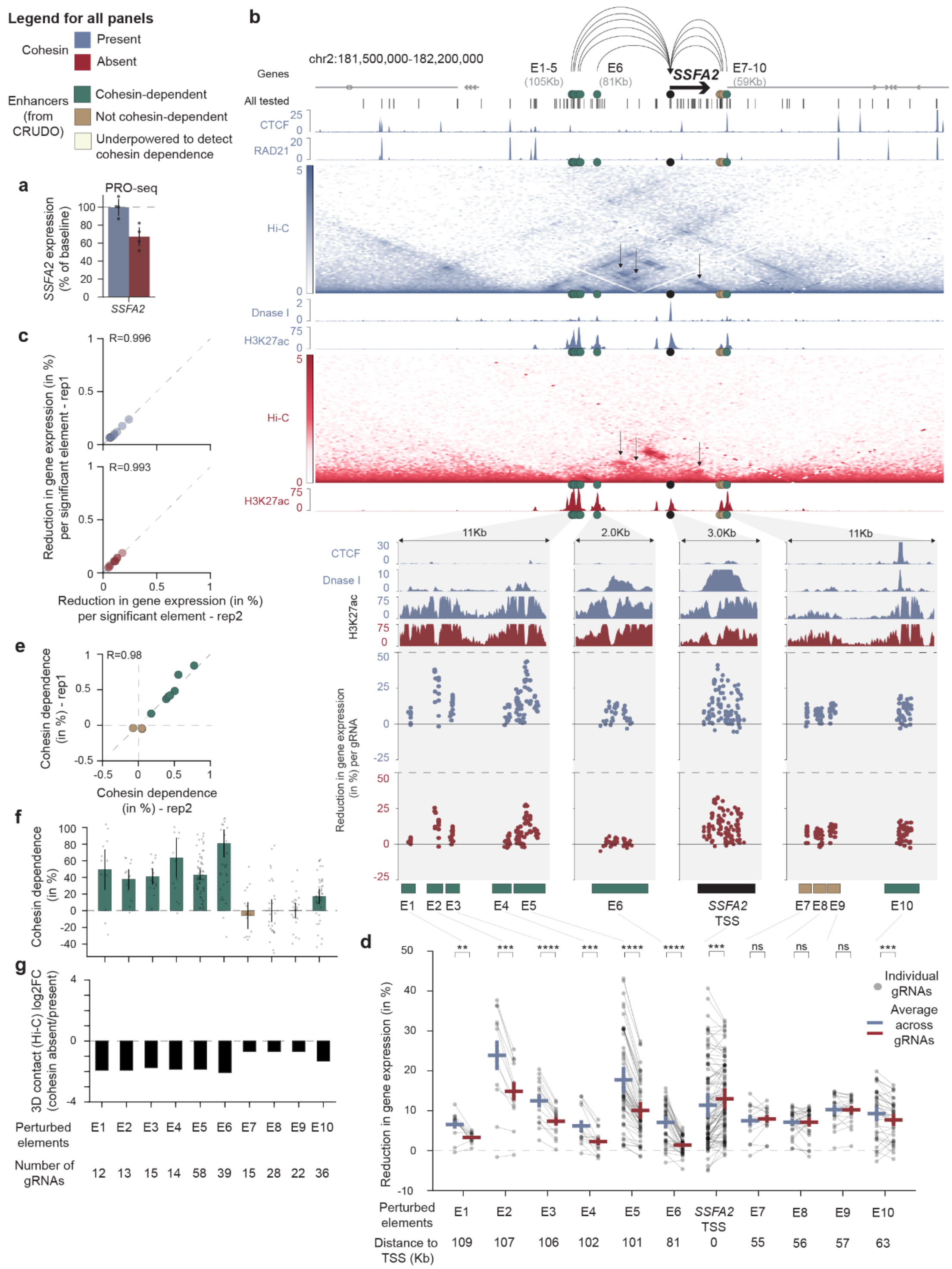


**Supplementary Fig. 9 Browser snapshot of the SSFA2 locus with CRUDO-FF screen effects**

**a,** *SSFA2* transcript levels in the presence (blue, 6h of DMSO treatment) or absence (red, 6h of auxin treatment) of cohesin, as measured by PRO-seq. Error bars: 95% c.i. across 4 biological replicates. **b,** *SSFA2* locus showing DNase I hypersensitivity, CTCF ChIP-seq, RAD21 ChIP-seq, SCALE-normalized Hi-C at 5Kb resolution (Hi-C depth = 1.1 (no auxin) and 1.5 (plus 6 hours auxin) billion contacts)[4], and H3K27ac ChIP-seq in the presence (blue) and/or absence (red) of cohesin. Bars at the top shows locations of all elements tested in the CRUDO-FF experiment. Zoom-ins show all elements that had a significant effect on *SSFA2* expression in cohesin present condition. Bars at bottom show

genomic locations of elements identified in the CRUDO-FF screen (teal: cohesin-dependent enhancers; taupe: not cohesin-dependent enhancers; ivory: underpowered to detect cohesin dependence enhancers; black: TSS). Dots show the measured effects on *SSFA2* expression for individual gRNAs targeting each element (% reduction in gene expression, normalized to the mean of all non-targeting gRNAs within each condition). **c**, Scatterplot shows correlation of effects on gene expression between two biological replicate experiments in the cohesin present (blue, top panel) and cohesin absent (red, bottom panel) conditions. Dots: all 10 elements that had significant effects on gene expression in the cohesin present condition. **d,** CRUDO-FF measured effect sizes on *SSFA2* expression for individual gRNAs in the presence (blue) or absence of cohesin (red). Horizontal lines and error bars: mean (+/- 95% c.i.) of effect sizes for each element. ns: $P_{BenjaminiHochberg} > 0.05$, **: $P_{BenjaminiHochberg} < 0.01$, ***: $P_{BenjaminiHochberg} < 0.001$, ****: $P_{BenjaminiHochberg} < 0.0001$ (gRNA-based paired 2-sample t-test). **e,** Similar to panel **c,** showing correlation of cohesin dependence between two biological replicate experiments. **f**, The level of cohesin dependence for each element, describing the change in the effect of an element on gene expression between the cohesin present versus absent conditions. Bar: mean across gRNAs for each element. Error bars: 95% c.i.. **g**, $Log_2$ fold-change in 3D contact frequencies (Hi-C SCALE-normalized counts, 5Kb resolution, Hi-C depth = 1.1 (no auxin) and 1.5 (plus 6 hours auxin) billion contacts) between the absence and presence of cohesin.

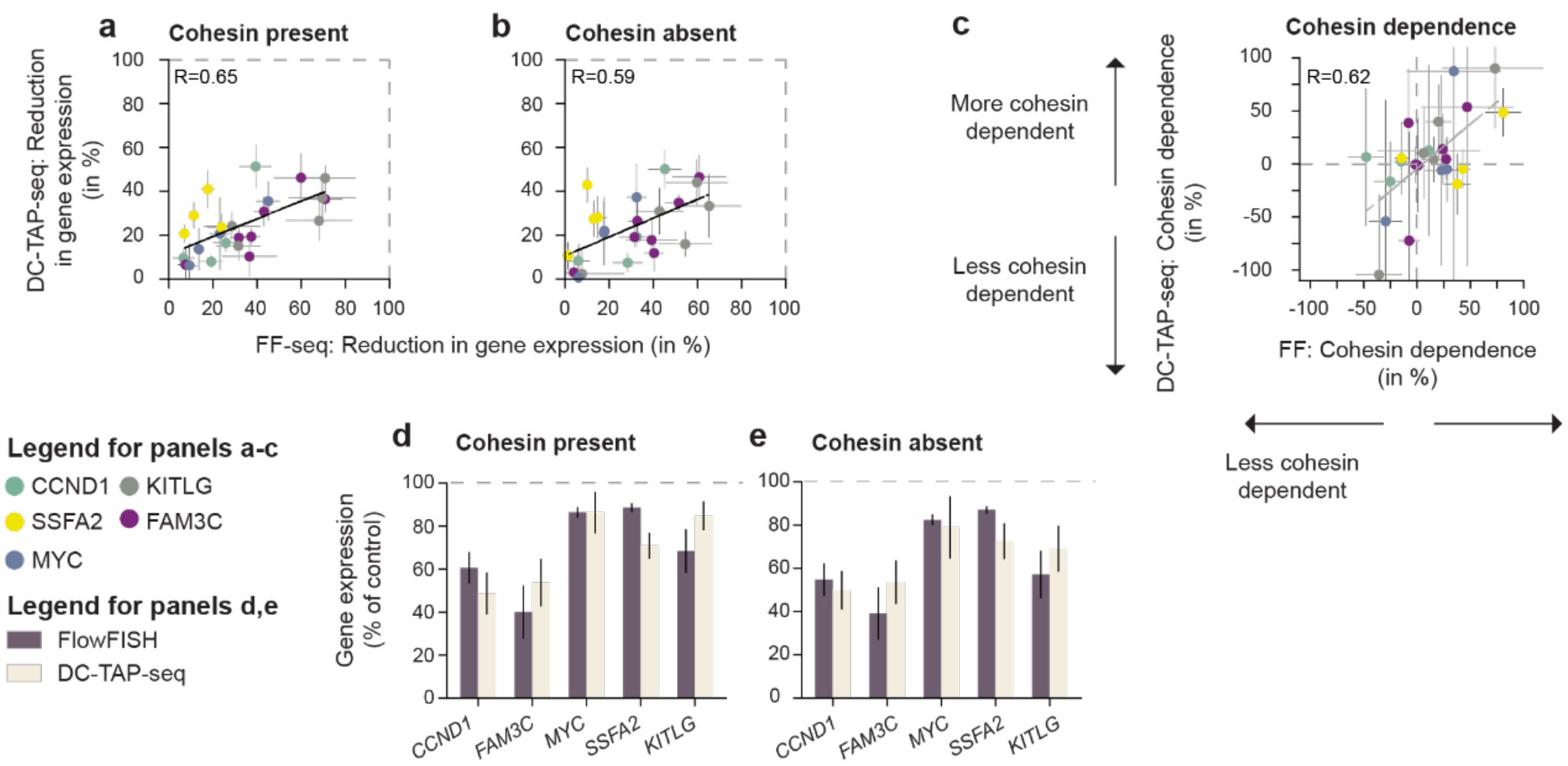


**Supplementary Fig. 10 Gene expression measurement correlation between FlowFISH and DC-TAP-seq**

**a,** Scatterplot shows correlation of effect size estimates of perturbing a subset of regulatory elements tested in the CRUDO-FF screen in the presence of cohesin as measured by FlowFISH and DC-TAP-seq. Colors represent target genes, Sage: CCND1, Gray: KITLG, Yellow: SSFA2, Black: FAM3C. Blue: Myc. **b,** Similar to **a**, but in the absence of cohesin. **c,** Similar to **a**, but of cohesin dependence as determined by FlowFISH and DCTAP-seq. **d,** Effect size estimates of perturbing the TSS of the 5 CRUDO-FF screen genes in the presence of cohesin as measured by FlowFISH (dark grey) and DC-TAP-seq (cream). **e,** Similar to **d**, but in the absence of cohesin.

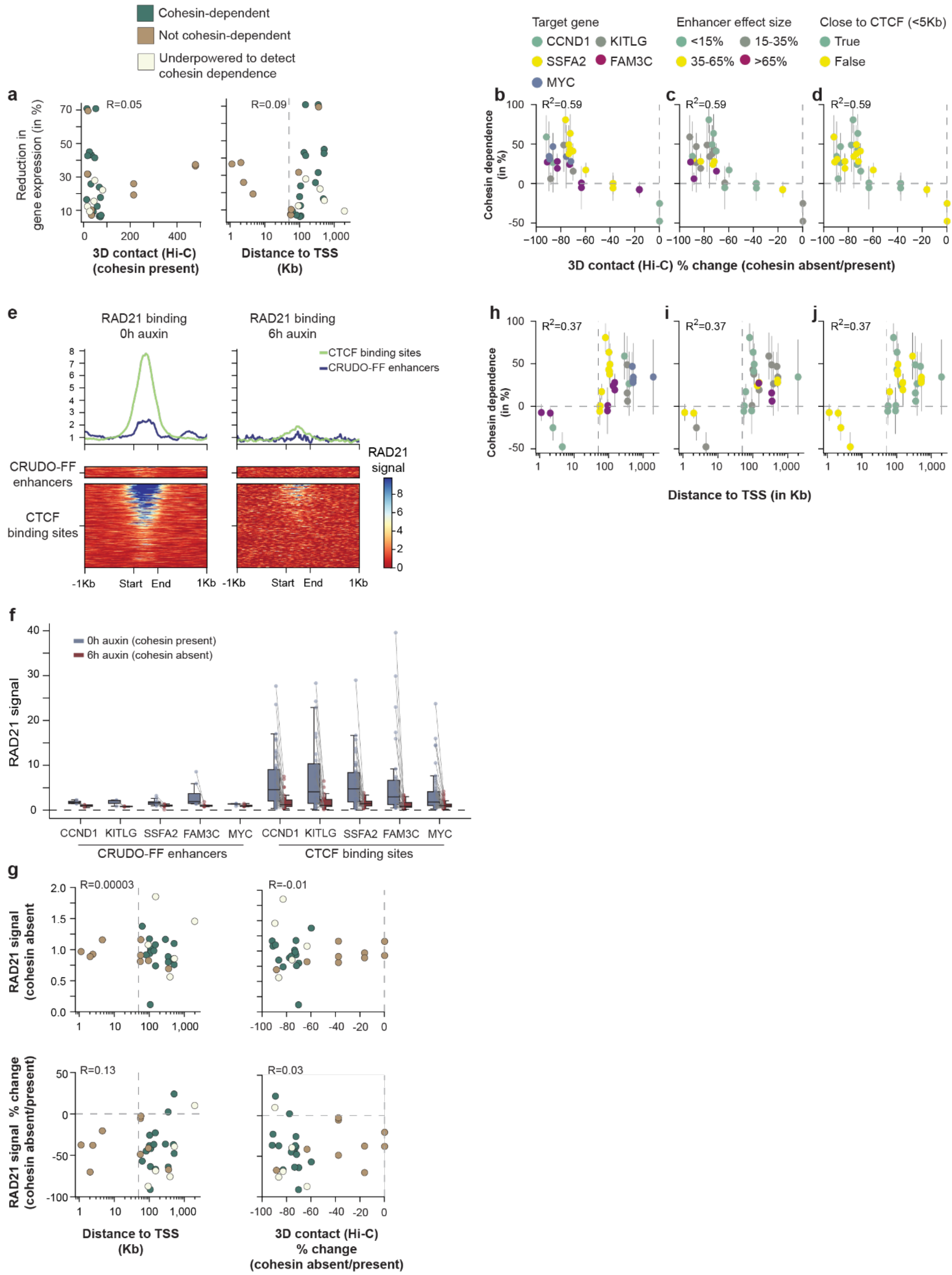


**Supplementary Fig. 11 CRUDO-FF screen results feature analyses**

**a,** Comparison of reduction in gene expression in the cohesin present condition with 3D contact (SCALE-normalized counts, 5Kb resolution, Hi-C depth = 1.1 billion contacts[4]) in the cohesin present condition (left panel) and genomic distance between the enhancer and target gene TSS in Kb ($\log_{10}$ scale, right panel) across all enhancers (n=30). **b,** Comparison of cohesin dependence with percent change in 3D contacts (Hi-C SCALE-normalized counts, 5Kb resolution, Hi-C depth = 1.1 (no auxin) and 1.5 (plus 6 hours auxin) billion contacts) between the cohesin absent versus present conditions across all enhancers (n=30). Colors represent target genes, Sage: CCND1, Gray: KITLG,

Yellow: SSFA2, Purple: FAM3C. Blue: MYC. **c,** Similar to **b**, but colors represent baseline enhancer effect sizes, Sage: <15%, Gray: 15-35%, Yellow: 35-65%, Black: >65%. **d,** Similar to **b**, but colors represent proximity to a CTCF binding site, Sage: <5Kb, Yellow: >5Kb. **e,** Heatmap and average signal profiles showing RAD21 ChIP-seq signal across CRUDO-FF enhancers and CTCF binding sites located within ±1 Mb of the TSS of each target gene, under control conditions (0h auxin, left panel) and following cohesin depletion (6h auxin, right panel). Each region is centered and scaled to 500bp plus 1Kb flanks. **f,** Boxplots show RAD21 ChIP-seq signal in cohesin present condition (0h auxin, blue) and cohesin absent condition (6h auxin, red) at CRUDO-FF enhancers and CTCF binding sites located within ±1 Mb of each target gene TSS. Each box represents the distribution of RAD21 signal across elements for a given gene and category (enhancer or CTCF site). Individual points represent signal at each element, with lines connecting matched values in the presence and absence of cohesin. **g,** Comparison of RAD21 signal in the absence of cohesin (top panel) and percent change in RAD21 signal (bottom panel) with genomic distance between the enhancer and target gene TSS in Kb (log10 scale) and percent change in 3D contact frequency as measured by Hi-C across all enhancers (n=30). Error bars: 95% c.i. across individual gRNAs (n=10-69). **h,** Comparison between cohesin dependence and genomic distance ($\log_{10}$ scale) from enhancer to target gene TSS across all enhancers (n=30). Colors represent target genes, Sage: CCND1, Gray: KITLG, Yellow: SSFA2, Purple: FAM3C. Blue: MYC. **i,** Similar to **h**, but colors represent baseline enhancer effect sizes, Sage: <15%, Gray: 15-35%, Yellow: 35-65%, Black: >65%. **j,** Similar to **h**, but colors represent proximity to a CTCF binding site, Sage: <5Kb, Yellow: >5Kb.

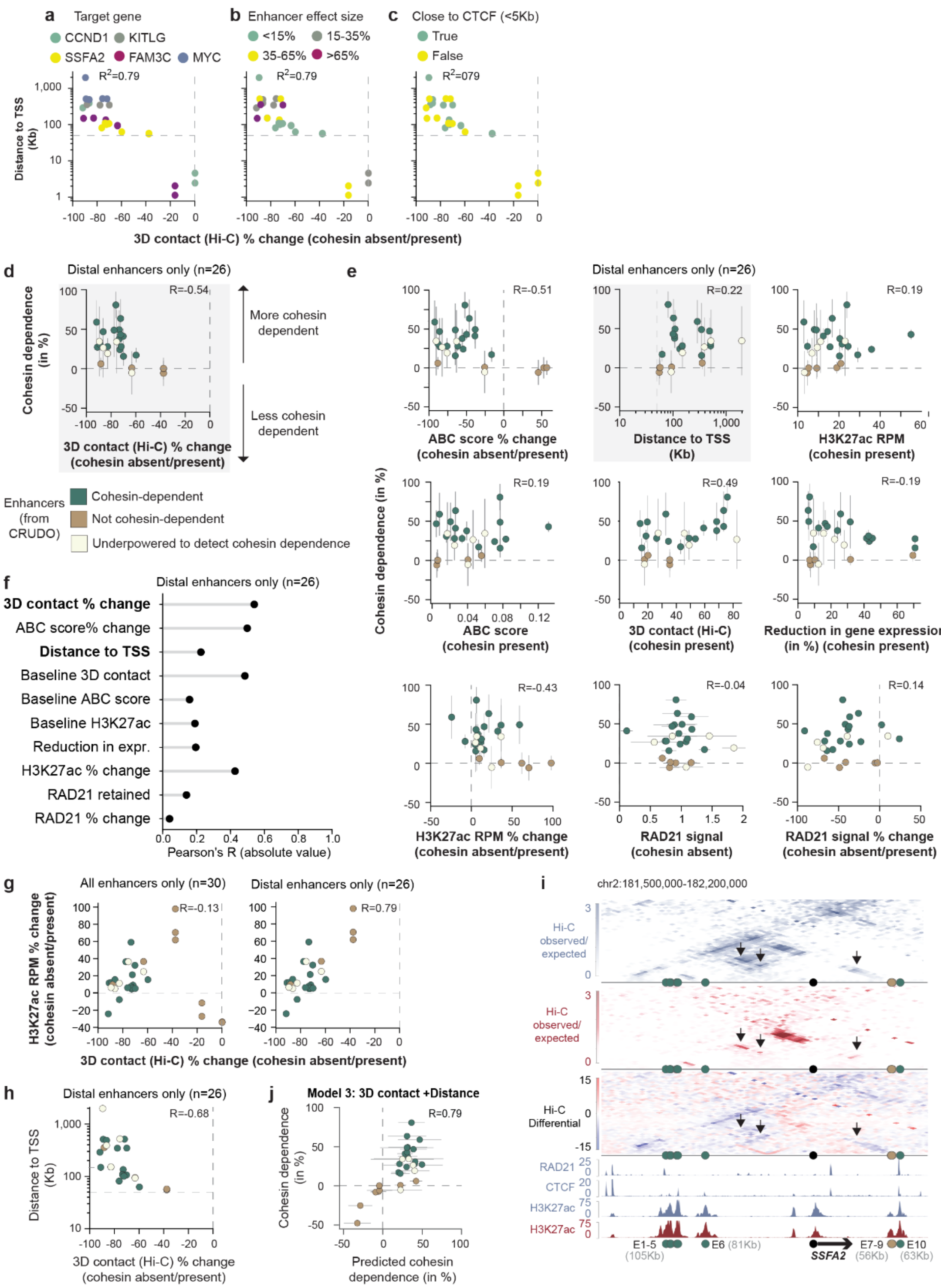


**Supplementary Fig. 12 CRUDO-FF screen results feature analyses for distal elements**

**a,** Genomic distance ($\log_{10}$ scale) from enhancer to target gene TSS versus the percent change in 3D contacts (Hi-C SCALE-normalized counts, 5Kb resolution, Hi-C depth = 1.1 (no auxin) and 1.5 (plus 6 hours auxin) billion contacts[4]) between the absence and presence of cohesin across all enhancers (n=30). Colors represent target genes, Sage: CCND1, Gray: KITLG, Yellow: SSFA2, Purple: FAM3C. Blue: MYC. **b,** Similar to **a**, but colors represent baseline enhancer effect sizes, Sage: <15%, Gray: 15-35%, Yellow: 35-65%, Black: >65%. **c,** Similar to **a**, but colors represent proximity

to a CTCF binding site, Sage: <5Kb, Yellow: >5Kb. **d,** Comparison of cohesin dependence with percent change in 3D contacts between the cohesin absent versus present conditions across distal enhancers (>50Kb, n=26). Error bars: 95% c.i. across individual gRNAs (n=10-69). **e,** Comparison of cohesin dependence with other genomic features across all distal enhancers (>50Kb, n=26). Top from left to right: Percent change in ABC score between the cohesin absent versus present conditions; Genomic distance between the enhancer and target gene TSS in Kb ($log_{10}$ scale); 3D contact (SCALE-normalized counts, 5Kb resolution, Hi-C depth = 1.1 billion contacts) in the cohesin present condition. Middle row from left to right: ABC-score in the cohesin present condition; H3K27ac ChIP-seq signal at the enhancer in the cohesin present condition; Reduction in gene expression in the cohesin present condition. Bottom row from left to right: Percent change in H3K27ac ChIP-seq signal at the enhancer between the cohesin absent versus present conditions; RAD21 signal at the enhancer in the absence of cohesin; Percent change in RAD21 signal at the enhancer between the cohesin absent versus present conditions. Error bars: 95% c.i. across individual gRNAs (n=10-69). **f**, A graph illustrating absolute Pearson's R values indicating the correlation between cohesin dependence and genomic features: percent change in H3K27ac ChIP-seq signal between cohesin absent versus present conditions, H3K27ac ChIP-seq signal, reduction in gene expression, ABC score, 3D contact (Hi-C SCALE-normalized counts, 5Kb resolution, Hi-C depth = 1.1 billion contacts), genomic distance between the enhancer and target gene TSS in Kb ($log_{10}$ scale), percent change in ABC score between cohesin absent versus present conditions, percent change in 3D contacts between cohesin absent versus present conditions, RAD21 signal at the enhancer in the absence of cohesin and percent change in RAD21 signal at the enhancer between the cohesin absent versus present conditions across distal enhancers only(>50Kb, n=26). **g,** Comparison of percent change in H3K27ac ChIP-seq signal at the enhancer between the cohesin absent versus present conditions with percent change in 3D contacts between the cohesin absent versus present conditions across all enhancers (n=30, left panel) and only distal enhancers (n=26, right panel). **h,** Genomic distance ($log_{10}$ scale) from enhancer to target gene TSS versus the percent change in 3D contacts (Hi-C SCALE-normalized counts, 5Kb resolution, Hi-C depth = 1.1 (no auxin) and 1.5 (plus 6 hours auxin) billion contacts) between the absence and presence of cohesin for distal enhancers only (n=26). **i,** *SSFA2* locus showing SCALE-normalized observed over expected Hi-C maps and the differential Hi-C map between observed cohesin absent and present at 5Kb resolution in the presence and absence of cohesin (Hi-C depth: 1.1 billion contacts in the absence of auxin and 1.5 billion contacts after 6 hours of auxin treatment). Also shown are CTCF ChIP-seq, RAD21 ChIP-seq (both in the presence of cohesin), and H3K27ac ChIP-seq in the presence (blue) and/or absence (red) of cohesin. CRUDO-enhancers are indicated at their genomic positions, Teal: Cohesin-dependent enhancers (n=7). Taupe: Not cohesin-dependent enhancers (n=3). Black: TSS. **j,** Comparison of cohesin dependence with OLS model 3 (Change in 3D contact + Distance to TSS) across all enhancers (n=30). Error bars: 95% c.i. across individual gRNAs (n=10-69).

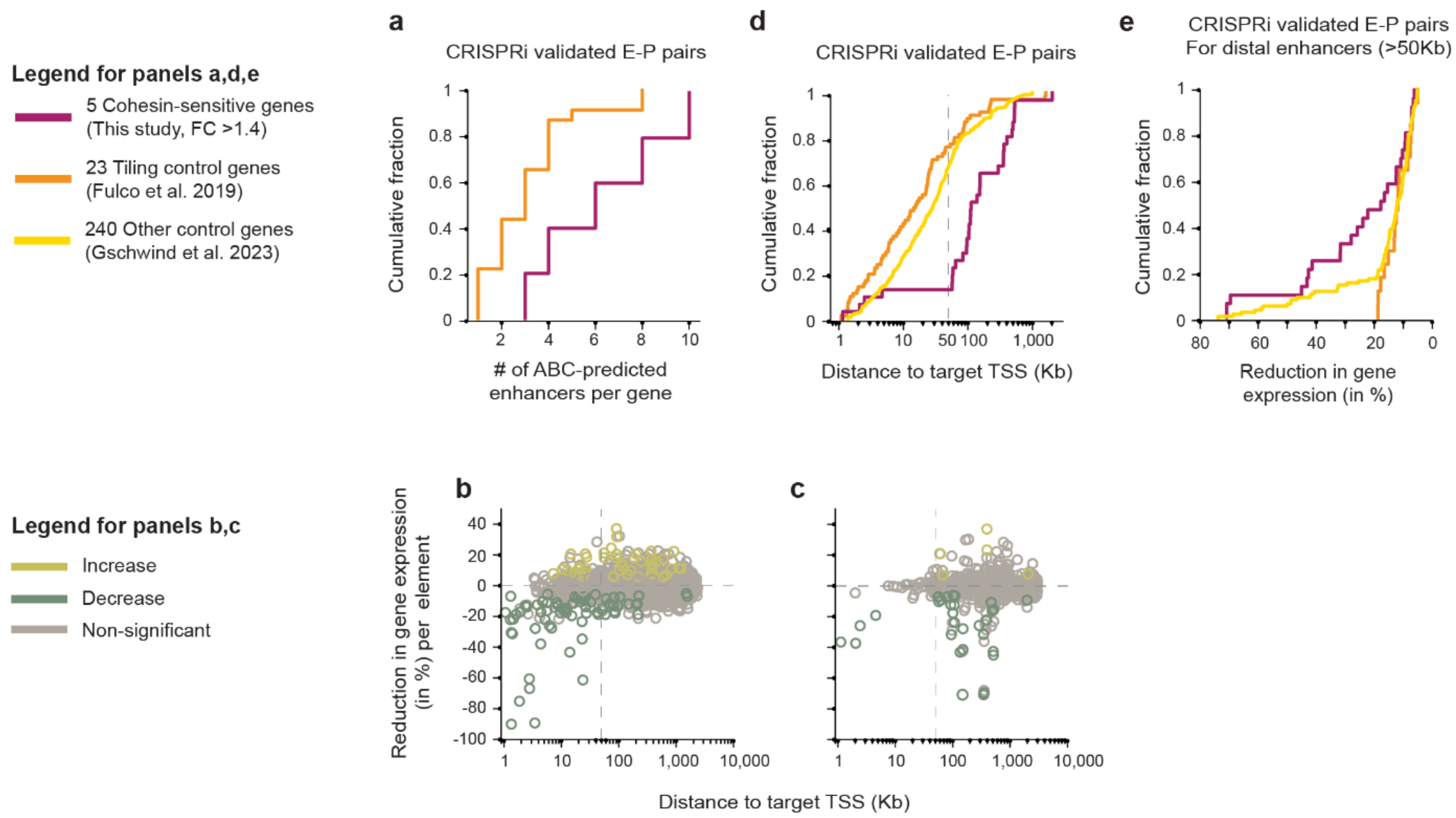


**Supplementary Fig. 13 Enhancer property comparison between cohesin-dependent and other genes**

**a,** Cumulative distribution plot of the number of enhancers per gene from comprehensive CRISPRi tiling data for the 5 cohesin-dependent genes in HCT116 cells (purple, n=30; this study) and for 23 "Tiling control" genes we previously studied in K562 cells, which were selected without respect to their cohesin dependence (orange, n=71;[5]). **b,** Enhancer effect sizes (as estimated by its perturbation) as a function of distance from element to target promoter, for all CRUDO-FF screen tested element-promoter pairs (n=1,039)**. c,** Enhancer effect sizes (as estimated by its perturbation) as a function of distance from element to target promoter, for all tested element-promoter pairs in the K562 "Tiling control" data set (n=4,914,). **d,** Cumulative distribution plot of the genomic distance from enhancer to target gene TSS for each significant pair from comprehensive CRISPRi tiling data for the 5 cohesin-dependent genes in HCT116 cells (purple, n=30; this study), for the 23 "Tiling control" genes (orange, n=71;[5]), and for the 240 "Other control" genes compiled previously (Gschwind et al.[1], which were selected without respect to their cohesin dependence and effects were measured using techniques other than CRISPRi-FF (yellow, n=347;[6–15]). **e,** Cumulave distribution plot of the effect size for each distal enhancer (>50Kb distance to TSS) in the CRISPRi validated enhancer-promoter pairs from our CRUDO-FF dataset (purple, n=30; this study), the "Tiling control" dataset, (orange, n=71;[5])), and the "Other control" datset[1,6–15] (yellow, n=347;[6–15]).

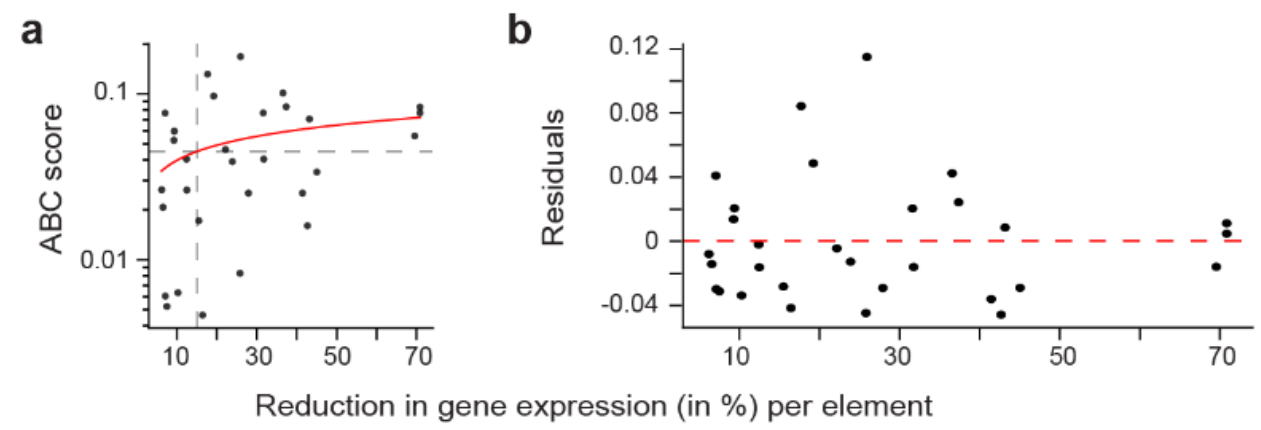


**Supplementary Fig. 14 Enhancer effect correlation**

**a,** ABC score as a function of FlowFISH-measured enhancer effect sizes for all CRUDO-FF screen entified enhancer-promoter pairs. The red line shows a power law fit to the data. Gray dashed lines indicate a 15% enhancer effect (x-axis) and the corresponding ABC score (0.045, y-axis) inferred from the power-law fit.. **b,** ABC score residuals (power law based predicted ABC score subtracted from the actual ABC score) as a function of FlowFISH-measured enhancer effect sizes for all CRUDO-FF screen identified enhancer-promoter pairs.

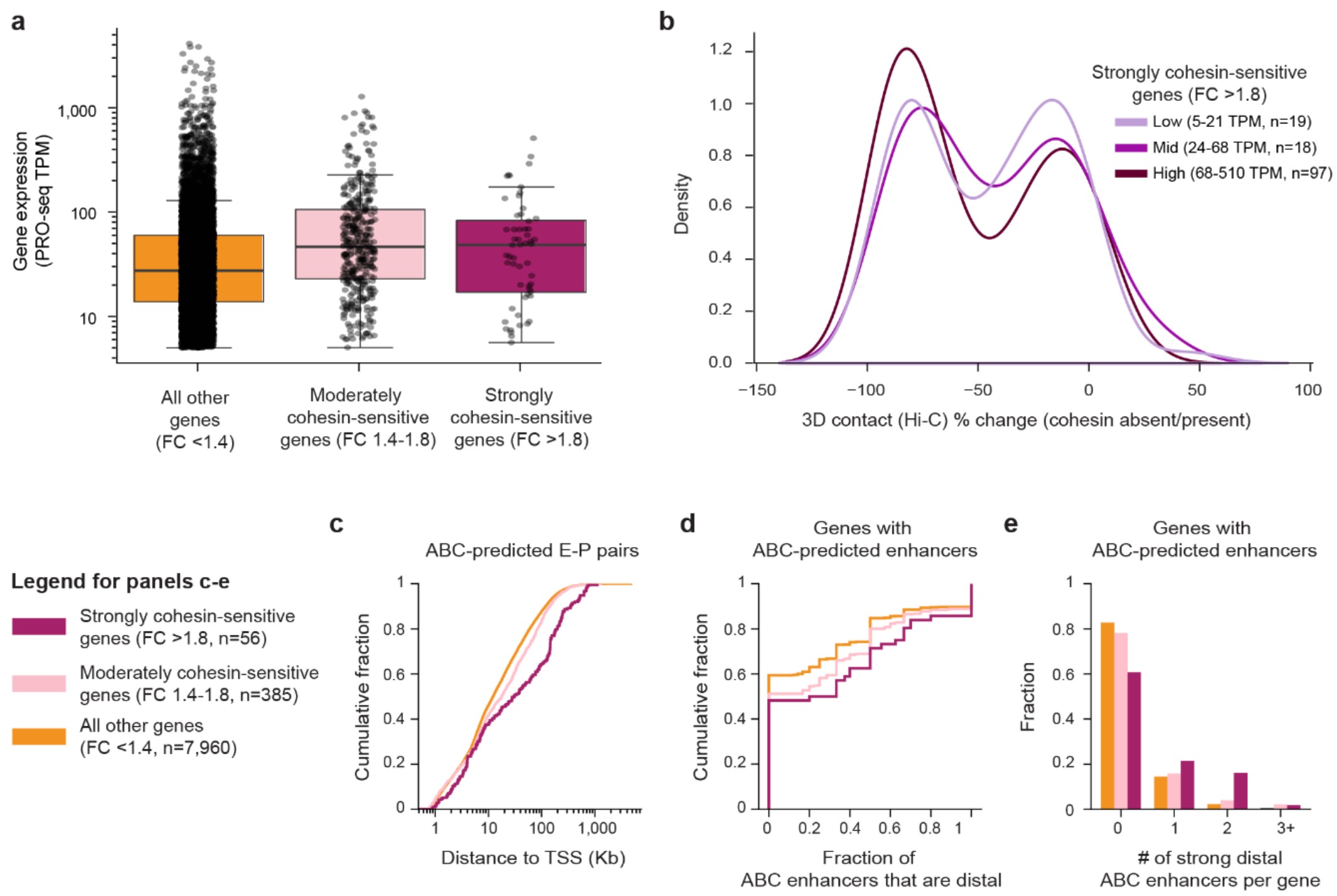


**Supplementary Fig. 15 ABC-predicted enhancer property comparison between strongly cohesin-dependent and all other genes**

**a,** Boxplot showing gene expression levels as measured by PRO-seq TPM for strongly cohesin-dependent genes (purple, n=56; defined as a >1.8-fold reduction in PRO-seq data in cohesin absent versus present conditions, TPM > 5), moderately cohesin-dependent genes (rose, n=385; defined as a 1.4-1.8-fold reduction in PRO-seq data in cohesin absent versus present conditions, TPM > 5), and all other genes (peach, n=7,960, TPM > 5). **b,** Density estimate (KDE) of the distribution of percent change in 3D contacts (Hi-C SCALE-normalized counts, 5Kb resolution, Hi-C depth = 1.1 (no auxin) and 1.5 (plus 6 hours auxin) billion contacts[4]) between the cohesin absent versus present conditions for strongly cohesin-dependent genes (n=56; defined as a >1.8-fold reduction in PRO-seq data in cohesin absent versus present conditions, TPM > 5) enhancer-gene pairs stratified by PRO-seq TPM into three equal-sized bins: low (violet, 5-20 TPM, n=19), mid (magenta, 20-68 TPM, n=18), and high (maroon, 68-510 TPM, n=19). **c,** Cumulative distribution plot of the distance from enhancer to target gene TSS for each ABC-predicted enhancer-promoter pair in HCT116 cells for strongly cohesin-dependent genes (purple, n=213; defined as a >1.8-fold reduction in PRO-seq data in cohesin absent versus present conditions, TPM > 5), moderately cohesin-dependent genes (rose, n=1,103; defined as a 1.4-1.8-fold reduction in PRO-seq data in cohesin absent versus present conditions, TPM > 5), and all other genes (peach, n=20,893, TPM > 5). **d,** Considering genes that have at least one predicted ABC enhancer, cumulative distribution plot of the fraction of predicted enhancers that are distal (>50Kb distance to TSS) for strongly cohesin-dependent genes (purple, n=56; defined as a >1.8-fold reduction in PRO-seq data in cohesin absent versus present conditions, TPM > 5), moderately cohesin-dependent genes (rose, n=385; defined as a 1.4-1.8-fold reduction in PRO-seq data in cohesin absent versus present conditions, TPM > 5), and all other genes (peach, n=7,960, TPM > 5). **e,** Considering genes that have at least one predicted ABC enhancer,
the fraction of genes that have 0, 1,2, or more than 3 ABC-predicted strong, distal enhancers (>15% effect size, >50Kb distance to TSS) for strongly cohesin-dependent genes (purple, n=56; defined as a >1.8-fold reduction in PRO-seq data in cohesin absent versus present conditions, TPM > 5), moderately cohesin-dependent genes (rose, n=385; defined as a 1.4-1.8-fold reduction in PRO-seq data in cohesin absent versus present conditions, TPM > 5), and all other genes (peach, n=7,960, TPM > 5).

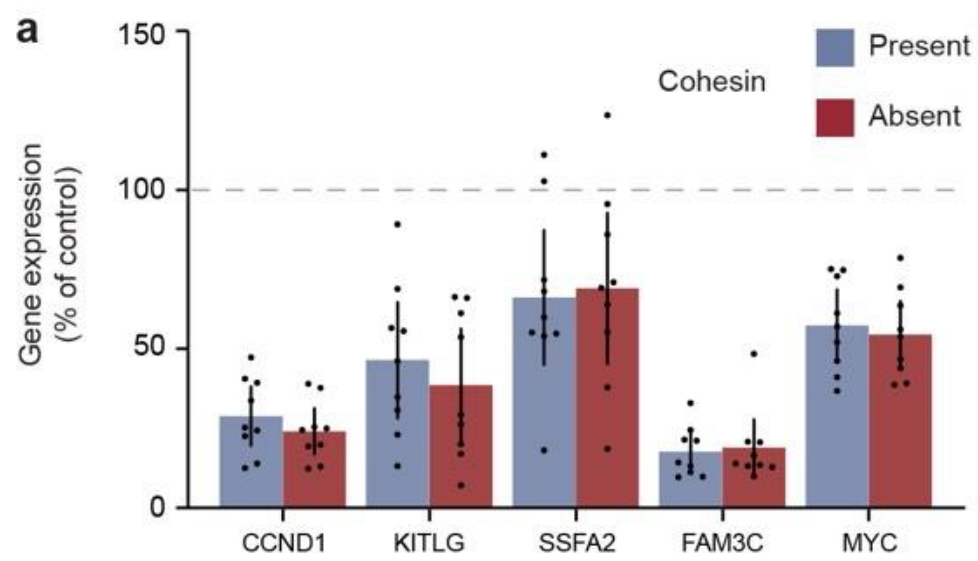


**Supplementary Fig. 16 Individual guide qPCR knockdown for CRUDO-FF scaling**

**a,** mRNA expression levels in the presence and absence of cohesin (6h of auxin treatment) as measured by qPCR for each of the CRUDO-FF screen genes in cells expressing individual gRNAs targeting the respective TSS for scaling of the CRUDO-FF effect size estimation. Error bars represent the 95% c.i.s.

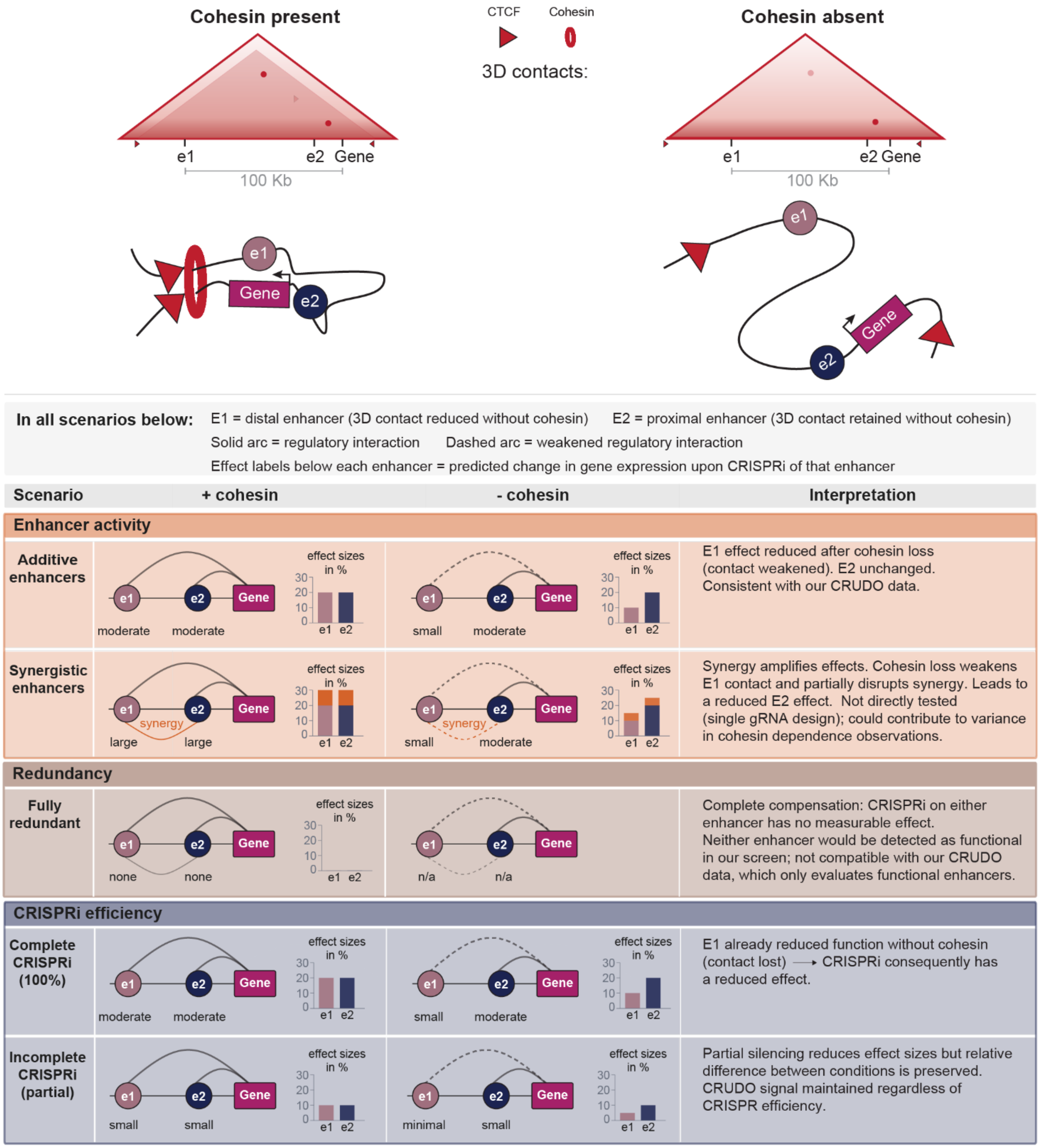


**Supplementary Fig. 17 Predicted CRUDO effects under alternative regulatory and experimental scenarios**

Top, schematic representation of a hypothetical genomic locus with two enhancers (E1, E2) and a target gene, shown as Hi-C contact maps and 3D chromatin models in the presence (left) and absence (right) of cohesin. E1 is a distal enhancer whose 3D contact with the promoter is reduced upon cohesin depletion; E2 is a proximal enhancer whose contact is retained. Below, predicted CRISPRi effect sizes for each enhancer under five scenarios: additive enhancers, synergistic enhancers, fully redundant enhancers, complete CRISPRi, and incomplete CRISPRi. Arc diagrams show regulatory interactions (solid = intact, dashed = weakened); bar charts show illustrative effect sizes (in %). The additive scenario is consistent with our CRUDO data. Full redundancy would render enhancers undetectable in our screen, and is thus not compatible with our data. CRISPRi efficiency does not confound cohesin-

dependence calls, as the relative difference between conditions is preserved. See **Supplementary Note 1** for full discussion.

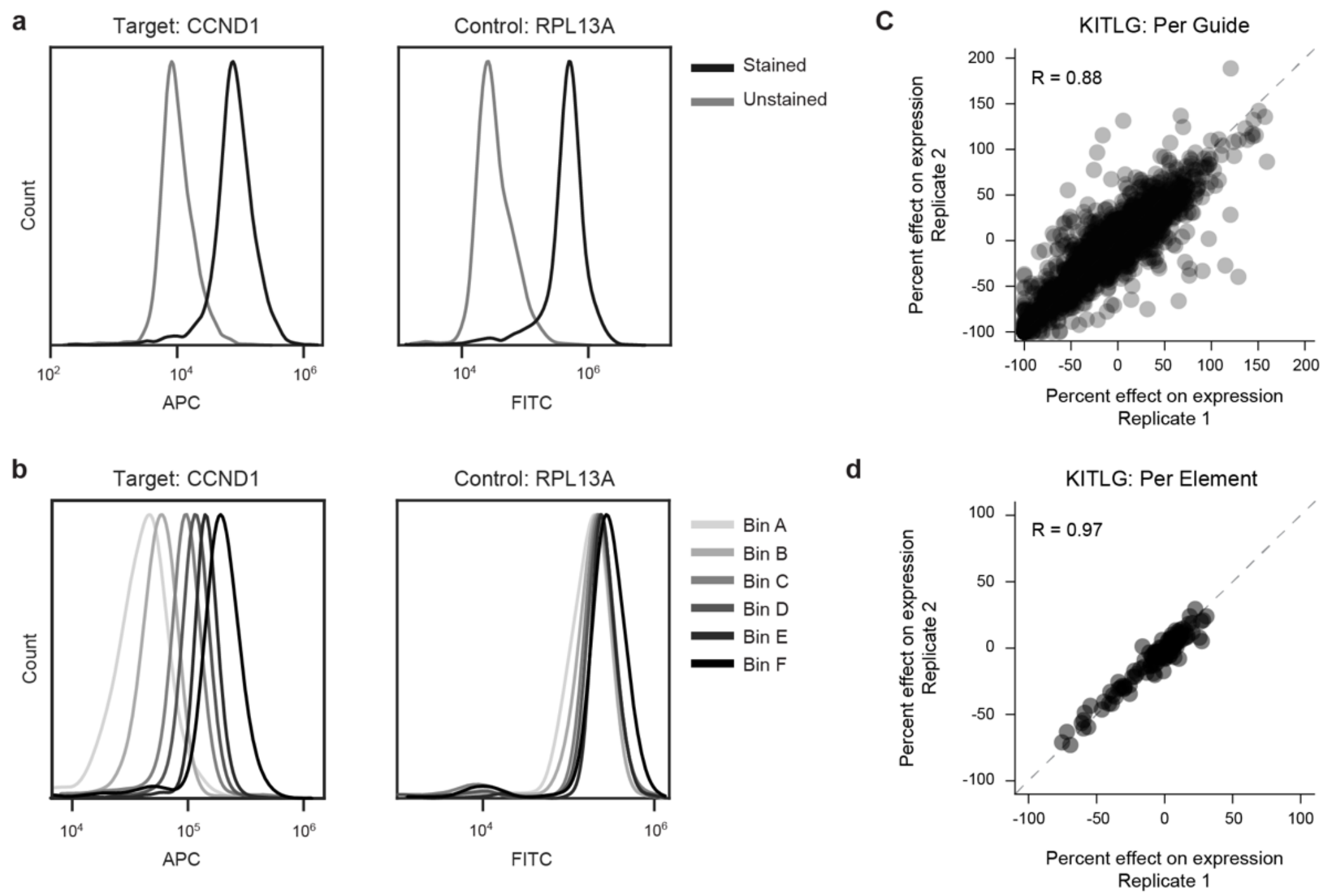


**Supplementary Fig. 18 FlowFISH quality controls**

**a,** Flow cytometry signal for cells with or without label probe for both target and control genes. Density plots have been normalized to the mode. **b,** Flow cytometry signal for cells after sorting by expression on CCND1 levels (with bin A having the cells with the lowest CCND1 expression and bin F the cells with the highest). Density plots have been normalized to the mode. **c,** Scatterplot of correlation of guide-level effects as estimated from two separate biological replicate experiments. **d,** Scatterplot of correlation of element-level effects as estimated from two separate biological replicate experiments.

## References

1. Gschwind, A. R. *et al.* An encyclopedia of enhancer-gene regulatory interactions in the human genome. *bioRxivorg* (2023) doi:10.1101/2023.11.09.563812.

2. Hansen, K. L. *et al.* Synergy between cis-regulatory elements can render cohesin dispensable for distal enhancer function. *bioRxiv* 2024.10.04.615095 (2024) doi:10.1101/2024.10.04.615095.

3. Rao, S. S. P. *et al.* Cohesin Loss Eliminates All Loop Domains. *Cell* **171**, 305-320.e24 (2017).

4. Mapping chromatin loops at base-pair resolution across over 100 human tissues. *In preparation.*

5. Fulco, C. P. *et al.* Activity-by-contact model of enhancer-promoter regulation from thousands of CRISPR perturbations. *Nat. Genet.* **51**, 1664–1669 (2019).

6. Schraivogel, D. *et al.* Targeted Perturb-seq enables genome-scale genetic screens in single cells. *Nat. Methods* **17**, 629–635 (2020).

7. Ulirsch, J. C. *et al.* Systematic functional dissection of common genetic variation affecting red blood cell traits. *Cell* **165**, 1530–1545 (2016).

8. Gasperini, M. *et al.* A genome-wide framework for mapping gene regulation via cellular genetic screens. *Cell* **176**, 1516 (2019).

9. Wakabayashi, A. *et al.* Insight into GATA1 transcriptional activity through interrogation of cis elements disrupted in human erythroid disorders. *Proc. Natl. Acad. Sci. U. S. A.* **113**, 4434–4439 (2016).

10. Klann, T. S. *et al.* CRISPR–Cas9 epigenome editing enables high-throughput screening for functional regulatory elements in the human genome. *Nat. Biotechnol.* **35**, 561–568 (2017).

11. Xie, S., Duan, J., Li, B., Zhou, P. & Hon, G. C. Multiplexed engineering and analysis of combinatorial enhancer activity in single cells. *Mol. Cell* **66**, 285-299.e5 (2017).

12. Thakore, P. I. *et al.* Highly specific epigenome editing by CRISPR-Cas9 repressors for silencing of distal regulatory elements. *Nat. Methods* **12**, 1143–1149 (2015).

13. Qi, Z. *et al.* Tissue-specific gene expression prediction associates vitiligo with SUOX through an active enhancer. *bioRxiv* (2018) doi:10.1101/337196.

14.Huang, J. *et al.* Dissecting super-enhancer hierarchy based on chromatin interactions. *Nat. Commun.* **9**, 943 (2018).

15.Xu, J. *et al.* Developmental control of polycomb subunit composition by GATA factors mediates a switch to non-canonical functions. *Mol. Cell* **57**, 304–316 (2015).
