# Supplementary material for: Quantitative changes in 3D contact predict changes in enhancer-promoter regulation upon cohesin depletion
Source: Nat Commun. 2026 Sep 29;17:11218. doi: 10.1038/s41467-026-77582-1 (PMC13720136; doi:10.1038/s41467-026-77582-1)
Supplement: Supplementary file 3 [file 41467_2026_77582_MOESM2_ESM.pdf]

Description of Additional Supplementary Files

Supplementary Data 1: Enhancer-gene predictions for HCT116 cells. Genome-wide enhancer-gene regulatory interactions predicted by the Activity-by-Contact (ABC) and ENCODE-rE2G models in HCT116 cells, in the presence (noAux) and absence (Aux) of cohesin (parts 1a-d).

Supplementary Data 2: Datasets of experimentally tested noncoding element-gene pairs. IGVF accession keys (2a); element-level results from the CCND1 pilot CRUDO-FlowFISH screen (2b), the full CRUDO-FlowFISH screens (2c), and the CRUDO-DC-TAP-seq validation screen (2d).

Supplementary Data 3: gRNA designs for validation experiments. gRNA target names and protospacer sequences (3a); mapping of each screen to its IGVF data accession (3b).

Supplementary Data 4: Dataset summary. All datasets used in this study, with data type, experiment and file accessions, use, and processing notes.

Supplementary Data 5: FlowFISH probe sets. RNA FlowFISH probe sets, listing target, assay ID, probe type, and detection label.

Supplementary Data 6: qPCR primer sequences. Forward and reverse primer sequences for each qPCR target.
