# Supplementary material for: Quantitative changes in 3D contact predict changes in enhancer-promoter regulation upon cohesin depletion
Source: Nat Commun. 2026 Sep 29;17:11218. doi: 10.1038/s41467-026-77582-1 (PMC13720136; doi:10.1038/s41467-026-77582-1)
Supplement: Supplementary file 4 [file 41467_2026_77582_MOESM9_ESM.pdf]

nature portfolio

Corresponding author(s): Jesse Engreitz

Last updated by author(s): Jul 7, 2026

Reporting Summary

Nature Portfolio wishes to improve the reproducibility of the work that we publish. This form provides structure for consistency and transparency in reporting. For further information on Nature Portfolio policies, see our Editorial Policies and the Editorial Policy Checklist.

Statistics

For all statistical analyses, confirm that the following items are present in the figure legend, table legend, main text, or Methods section.

n/a	Confirmed	
☐	☒	The exact sample size (*n*) for each experimental group/condition, given as a discrete number and unit of measurement
☐	☒	A statement on whether measurements were taken from distinct samples or whether the same sample was measured repeatedly
☐	☒	The statistical test(s) used AND whether they are one- or two-sided<br>*Only common tests should be described solely by name; describe more complex techniques in the Methods section.*
☐	☒	A description of all covariates tested
☐	☒	A description of any assumptions or corrections, such as tests of normality and adjustment for multiple comparisons
☐	☒	A full description of the statistical parameters including central tendency (e.g. means) or other basic estimates (e.g. regression coefficient) AND variation (e.g. standard deviation) or associated estimates of uncertainty (e.g. confidence intervals)
☐	☒	For null hypothesis testing, the test statistic (e.g. *F*, *t*, *r*) with confidence intervals, effect sizes, degrees of freedom and *P* value noted<br>*Give P values as exact values whenever suitable.*
☒	☐	For Bayesian analysis, information on the choice of priors and Markov chain Monte Carlo settings
☐	☒	For hierarchical and complex designs, identification of the appropriate level for tests and full reporting of outcomes
☐	☒	Estimates of effect sizes (e.g. Cohen's *d*, Pearson's *r*), indicating how they were calculated

Our web collection on statistics for biologists contains articles on many of the points above.

Software and code

Policy information about availability of computer code

Data collection	The Sony MA900FP cell sorter (used for FlowFISH) was run using “Cell Sorter Software” v3.2. Other instruments were used with the manufacturer-supplied intrinsic software, including Applied Biosystems QuantStudio 5 (for qRT-PCR).
Data analysis	gRNAs were designed using CRISPRDesigner, https://github.com/EngreitzLab/CRISPRDesigner. TAP-seq primers were designed using all packages required for running TAPseq 1.16.0 primer design pipeline, https://bioconductor.org/packages/release/bioc/html/TAPseq.html. CRUDO-FlowFISH data was processed using all packages required for running the CRISPRi-FlowFISH pipeline, https://github.com/EngreitzLab/crispri-flowfish). CRUDO-TAP-seq data was processed using all packages required for running the TAP-seq analysis pipeline (https://github.com/argschwind/TAPseq_workflow) and CellRanger 6.0.0. We additionally used Python 3.9.16, pybedtools 0.12.0, pysam 0.23.0, pandas 2.2.3, NumPy 2.0.2, Matplotlib 3.9.4, seaborn 0.13.2, SciPy 1.13.1, statsmodels 0.14.4, hic-straw 1.3.1, CoolBox 0.10.3, and FlowJo v10.8.1. for custom code developed for this study (https://github.com/philinegu/cohesin_CRUDO_workflow).

For manuscripts utilizing custom algorithms or software that are central to the research but not yet described in published literature, software must be made available to editors and reviewers. We strongly encourage code deposition in a community repository (e.g. GitHub). See the Nature Portfolio guidelines for submitting code & software for further information.

Data

Policy information about availability of data

All manuscripts must include a data availability statement. This statement should provide the following information, where applicable:

- Accession codes, unique identifiers, or web links for publicly available datasets
- A description of any restrictions on data availability
- For clinical datasets or third party data, please ensure that the statement adheres to our policy

The data generated in this study have been deposited in the following databases under the specified accession codes (see Supplementary Data File 4 for detailed data summary).

CRISPRi-FlowFISH data on the IGVF Data Portal:
Accession numbers in Supplementary Data File 2a (https://data.igvf.org/)

CRISPRi-DC-TAP-seq data on the IGVF Data Portal:
Accession numbers in Supplementary Data File 2a (https://data.igvf.org/)

ENCODE Hi-C from the ENCODE Portal:
ENCSR958BEA (https://www.encodeproject.org/experiments/ENCSR958BEA/),
ENCSR087JOM (https://www.encodeproject.org/experiments/ENCSR087JOM/)

DNase-seq from the ENCODE Portal:
ENCFF166PCV (https://www.encodeproject.org/files/ENCFF166PCV/),
ENCFF619LVU (https://www.encodeproject.org/files/ENCFF619LVU/)

H3K27ac ChIP-seq data from Gene Expression Omnibus:
GSM2809617 (https://www.ncbi.nlm.nih.gov/geo/query/acc.cgi?acc=GSM2809617),
GSM2809618 (https://www.ncbi.nlm.nih.gov/geo/query/acc.cgi?acc=GSM2809618)
RAD21 ChIP-seq data from Gene Expression Omnibus:
GSM2809609 (https://www.ncbi.nlm.nih.gov/geo/query/acc.cgi?acc=GSM2809609),
GSM2809610 (https://www.ncbi.nlm.nih.gov/geo/query/acc.cgi?acc=GSM2809610)

CTCF ChIP-seq data from ENCODE:
ENCFF072BUT (https://www.encodeproject.org/files/ENCFF072BUT/)

PRO-seq data from Gene Expression Omnibus:
GSE106886 (https://www.ncbi.nlm.nih.gov/geo/query/acc.cgi?acc=GSE106886)

ENCODE-rE2G CRISPRi benchmarking set:
https://github.com/EngreitzLab/CRISPR_comparison/blob/main/resources/crispr_data/EPCrisprBenchmark_combined_data.training_K562.GRCh38.tsv.gz

Source data for each figure are provided in a combined Source Data file, and the associated code and resources are available on GitHub: https://github.com/philinegu/cohesin_CRUDO_workflow.

Research involving human participants, their data, or biological material

Policy information about studies with human participants or human data. See also policy information about sex, gender (identity/presentation), and sexual orientation and race, ethnicity and racism.

Reporting on sex and gender	not applicable
Reporting on race, ethnicity, or other socially relevant groupings	not applicable
Population characteristics	not applicable
Recruitment	not applicable
Ethics oversight	not applicable

Note that full information on the approval of the study protocol must also be provided in the manuscript.

Field-specific reporting

Please select the one below that is the best fit for your research. If you are not sure, read the appropriate sections before making your selection.

☒ Life sciences ☐ Behavioural & social sciences ☐ Ecological, evolutionary & environmental sciences

For a reference copy of the document with all sections, see nature.com/documents/nr-reporting-summary-flat.pdf

Life sciences study design

All studies must disclose on these points even when the disclosure is negative.

Sample size	Sample size for FlowFISH (2-4 replicates, >10 gRNAs per element, 463 non-targeting control guides, etc.) were calculated based on Fulco, C. P., Nasser, J., et al., Activity-by-contact model of enhancer–promoter regulation from thousands of CRISPR perturbations., Nature Genetics 51, 1664–1669., 2019. Sample size for TAP-seq (2 replicates, 3 gRNAs per element, 50 non-targeting control guides, etc.) were calculated based on Schraivogel, D., Gschwind, A.R., et al., Targeted Perturb-seq enables genome-scale genetic screens in single cells., Nature Methods 17, 629–635., 2020.
Data exclusions	No data that passed uniformly-applied quality control filters was excluded from this study.
Replication	The pilot CRUDO-FlowFISH screen was performed as a single experiment and the large-scale CRUDO-FlowFISH screens were performed in 2-4 replicates (with measurements of multiple gRNAs for each target and additional validation of a subset of TSS targeting gRNAs validated for knock down efficacy by RT-qPCR.).
Randomization	Randomization was not necessary for these studies because they were performed on homogeneous cell lines (e.g. HCT-116).
Blinding	Blinding was not necessary for these studies because quantitation was performed using unbiased approaches (established methods for FlowFISH, EdgeR for bulk RNAseq, qRT-PCR ddCT, etc.).

Reporting for specific materials, systems and methods

We require information from authors about some types of materials, experimental systems and methods used in many studies. Here, indicate whether each material, system or method listed is relevant to your study. If you are not sure if a list item applies to your research, read the appropriate section before selecting a response.

Materials & experimental systems

n/a	Involved in the study	
☒	☐	Antibodies
☐	☒	Eukaryotic cell lines
☒	☐	Palaeontology and archaeology
☒	☐	Animals and other organisms
☒	☐	Clinical data
☒	☐	Dual use research of concern
☒	☐	Plants

Methods

n/a	Involved in the study	
☒	☐	ChIP-seq
☐	☒	Flow cytometry
☒	☐	MRI-based neuroimaging

Eukaryotic cell lines

Policy information about cell lines and Sex and Gender in Research

Cell line source(s)	HCT-116 RAD21-mAiD-mClover cell line was generated in the Masato Kanemaki lab (Natsume et al., Rapid Protein Depletion in Human Cells by Auxin-Inducible Degron Tagging with Short Homology Donors, Cell Reports, 15, 210-218, 2016) and was gifted to Erez Lieberman-Aiden (Rao et al., Cohesin Loss Eliminates All Loop Domains., Cell, 171, 259-496, 2017).
Authentication	Cell lines were authenticated in Rao et al., Cohesin Loss Eliminates All Loop Domains., Cell, 171, 259-496, 2017. We further authenticated each line by analysis of functionality (high transfectability and protein expression for HEK293T) and mRNA expression levels.
Mycoplasma contamination	Confirmed negative
Commonly misidentified lines (See ICLAC register)	None

Plants

Seed stocks

not applicable

Novel plant genotypes

not applicable

Authentication

not applicable

Flow Cytometry

Plots

Confirm that:

☒ The axis labels state the marker and fluorochrome used (e.g. CD4-FITC).

☒ The axis scales are clearly visible. Include numbers along axes only for bottom left plot of group (a 'group' is an analysis of identical markers).

☒ All plots are contour plots with outliers or pseudocolor plots.

☒ A numerical value for number of cells or percentage (with statistics) is provided.

Methodology

Sample preparation

For scoring and sorting BFP+ CRISPRi cells after doxycycline treatement, cells were harvested and resuspended in HCT-116 medium at a density of ~1-3e6 cells/ml. FlowFISH was performed according to our established methods (Fulco, C. P., Nasser, J., et al., Activity-by-contact model of enhancer–promoter regulation from thousands of CRISPR perturbations., Nature Genetics 51, 1664–1669., 2019). Briefly, we used the PrimeFlow RNA Assay Kit (Thermo Fisher; Catalog number: 88-18005) according to the manufacturer's instructions, using ThermoFisher probesets listed in Supplementary Table 4. We diluted the stained and washed cells in PBS with 0.5% BSA to a concentration of 2x10^7 cells/ml and filtered using a 30µm filter (CellTrics, Catalog number 04-004-2326).

Instrument

Diagnostic FACS for BFP positive cells was performed on a Beckman CytoFLEX S Flow Cytometer. The sort for BFP+ cells was performed on a MoFlo Astrios EQ Cell Sorter. For FlowFISH, we used a SONY MA900 sorter.

Software

FACS runs were performed using the manufacturer software package (e.g. CytExpert for diagonstic FACS). Basic analysis was performed using FloJo or FCSalyzer 0.9.22-alpha. FlowFISH analysis was performed using a Maximum Likelihood Estimator approach, according to our established methods (Fulco et al. Nature Genetics 51, 1664–1669 (2019). In brief, we counted gRNAs in each bin using Bowtie to map reads to a custom index, normalized gRNA counts in each bin by library size, then used a maximum-likelihood estimation approach to compute the effect size for each gRNA. We used the limited-memory Broyden–Fletcher–Goldfarb–Shanno algorithm (implemented in the R stats4 package) to estimate the most likely log-normal distribution that would have produced the observed guide counts, and the effect size for each gRNA is the mean of its log-normal fit divided by the average of the means from all negative-control gRNAs. As previously described, we scaled the effect size of each gRNA in a screen linearly, based on knockdown measurements by qPCR of the three stongest gRNAs). We averaged the effect sizes of each gRNA across replicates and computed the effect size of an element as the average of all gRNAs targeting that element. We assessed significance using a two-sided t-test comparing the mean effect size of all gRNAs in a candidate element to all negative-control guides. We computed the false-discovery rate (FDR) for elements using the Benjamini–Hochberg procedure and used an FDR threshold of 0.05 to call significant regulatory effects.

Cell population abundance

Cells were not a mix of cell types, and were all HCT-116 RAD21-mAiD-mClover-KRAB-dCAS9. For BFP+ cell sorting, almost all cells had a stronger PB450-A signal than non-dox treated controls, and we sorted for cells with the top 15% PB450-A signal. For FlowFISH cells in the with different CRISPRi perturbation guides were sorted into bins by percentage of normalized target gene to control signal, as described below

Gating strategy

For sorting BFP+ cells, live cells were gated by SSC-A and FSC-A. BFP-positive cells were identified by comparing Doxycycline-treated HCT-116 RAD21-mAiD-mClover-KRAB-dCAS9 to Doxycycline-treated HCT-116 RAD21-mAiD-mClover control cells, and the top 10% of the BFP-positive peak (PB450-A) were sorted. The gating strategy is shown in Extended Data Fig. 3b. FlowFISH parameters were essentially as following Fulco, C. P., Nasser, J., et al., Activity-by-contact model of enhancer–promoter regulation from thousands of CRISPR perturbations., Nature Genetics 51, 1664–1669., 2019, with the gating strategy illustrated in that publication and Extended Data Fig. 14. Briefly, to control for differences in staining efficiency for each cell, we normalized the fluorescence associated with the gene of interest to that of the control gene. Specifically, we used the color compensation tool to subtract a portion of each cell's AF647 signal based on the intensity of its AF488 signal such that the mean AF488 signal in the top and bottom 25% of cells based on AF647 was within 10%. If necessary, we then reduced the level of compensation until the fraction of cells with AF647 signal equal to 0 was no more than 5%. We set the gates for each bin on the compensated signal with bins numbered according to the percentiles A: 0-10%, B:10-20%, C:35-45%, D:55-65%, E:80-90%, F:90-100%.

☒ Tick this box to confirm that a figure exemplifying the gating strategy is provided in the Supplementary Information.
